# Flow-Based Fmoc-SPPS Preparation and SAR Study of Cathelicidin-PY Reveals Selective Antimicrobial Activity

**DOI:** 10.3390/molecules28041993

**Published:** 2023-02-20

**Authors:** Shama Dissanayake, Junming He, Sung H. Yang, Margaret A. Brimble, Paul W. R. Harris, Alan J. Cameron

**Affiliations:** 1 School of Chemical Sciences and School of Biological Sciences, The University of Auckland, 23 Symonds St., Auckland 1142, New Zealand; 2 Maurice Wilkins Centre for Molecular Biodiscovery, The University of Auckland, Auckland 1142, New Zealand

**Keywords:** cathelicidin, flow-based SPPS, disulfide, lactam, AMP, *E. coli*, microbiome

## Abstract

Antimicrobial peptides (AMPs) hold promise as novel therapeutics in the fight against multi-drug-resistant pathogens. Cathelicidin-PY (NH_2_-RKCNFLCKLKEKLRTVITSHIDKVLRPQG-COOH) is a 29-residue disulfide-cyclised antimicrobial peptide secreted as an innate host defence mechanism by the frog *Paa yunnanensis* (PY) and reported to possess broad-spectrum antibacterial and antifungal properties, exhibiting low cytotoxic and low hemolytic activity. Herein, we detail the total synthesis of cathelicidin-PY using an entirely on-resin synthesis, including assembly of the linear sequence by rapid flow Fmoc-SPPS and iodine-mediated disulfide bridge formation. By optimising a synthetic strategy to prepare cathelicidin-PY, this strategy was subsequently adapted to prepare a bicyclic head-to-tail cyclised derivative of cathelicidin-PY. The structure-activity relationship (SAR) of cathelicidin-PY with respect to the *N*-terminally positioned disulfide was further probed by preparing an alanine-substituted linear analogue and a series of lactam-bridged peptidomimetics implementing side chain to side chain cyclisation. The analogues were investigated for antimicrobial activity, secondary structure by circular dichroism (CD), and stability in human serum. Surprisingly, the disulfide bridge emerged as non-essential to antimicrobial activity and secondary structure but was amenable to synthetic modification. Furthermore, the synthetic AMP and multiple analogues demonstrated selective activity towards Gram-negative pathogen *E. coli* in physiologically relevant concentrations of divalent cations.

## 1. Introduction

The discovery of penicillin in 1928 began the golden age of natural product development and antibiotic discovery, peaking in the mid-1950s [1]. Since then, the average life expectancy of humans has been extended by 23 years, which is deemed one of the greatest discoveries in modern medicinal chemistry [1]. However, antibiotic misuse has resulted in the rapid development of antimicrobial resistance (AMR), giving rise to superbugs that are currently untreatable [2]. Therefore, medicinal chemists have broadened their horizons in recent decades. Antimicrobial peptides (AMPs) have gained popularity as an attractive alternative to conventional small-molecule antibiotics to target multidrug resistant (MDR) pathogens. AMPs are an integral part of the innate immune system produced by all living organisms, also known as host defence peptides (HDP) [3]. The HDP arsenal of vertebrates contains two main families of AMPs: the defensins and cathelicidins, characterised by the number of disulfide bridges present in their sequences. Defensin peptides possess multiple disulfide bridges, whereas cathelicidins are either linear or limited to a single disulfide bridge [4,5]. Cathelicidins are stored in an inactive precursor form within the secretory granules of neutrophils, where they readily undergo maturation in response to a foreign bacterial invasion [3,6]. Phagocytes initiate proteolytic cleavage of the inactive pro-sequence via serine proteases and elastase to readily form and release the mature cathelicidin peptide as an innate protective mechanism [7].

**Figure 1:**
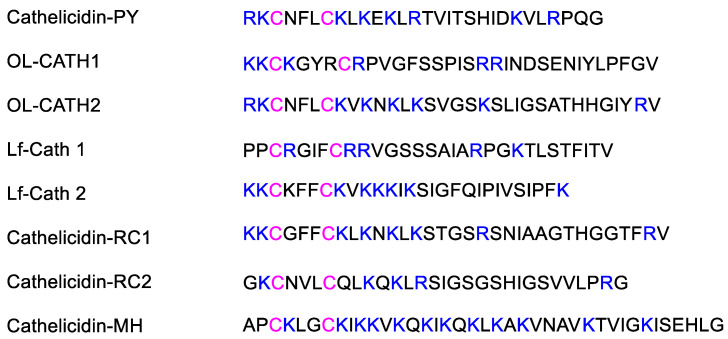
Sequence alignment of recently discovered amphibian cathelicidins, mature peptides shown. Blue denotes cationic residues and pink denotes the cysteine residues forming a disulfide bond.

Cathelicidin peptides typically demonstrate a broad spectrum of antimicrobial activity towards Gram-positive bacteria (e.g., *Staphylococcus aureus* [8], *Enterococcus faecalis* [9], and *Bacillus cereus* [10])*,* Gram-negative bacteria (e.g., *Escherichia coli* [11], *Pseudomonas aeruginosa* [12], and *Salmonella enterica* [13]), and fungi (e.g., *Candida albicans* [14]). Furthermore, these have shown promising anti-inflammatory and specific immune modulatory capabilities in immunocytes [15], as well as antiviral activity against human immunodeficiency virus (HIV) [16], influenza A virus (IAV) [17], respiratory syncytial virus (RSV) [18], human rhinovirus (HRV) [19], herpes simplex virus (HSV) [20], Zika virus (ZIKV), and hepatitis C virus (HCV) [21,22,23]. They are highly cationic (between +4 and +13 charges in physiological conditions) and exhibit membrane-lytic mechanisms of action [11,24]. Cathelicidin-TK, isolated from the salamander *Tylototriton kweichowensis*, is the only known anionic cathelicidin, exhibiting wound healing properties rather than typical antimicrobial activity [25]. Since the first reported cathelicidin peptide in 1989 by Gennaro et al. [26], more than 150 cathelicidin peptides have been isolated from over 30 different vertebrate species and are found in almost all species of mammals [27,28]. The occurrence of cathelicidins in amphibians, however, was not reported until 2012 by Hao et al. [29] from the frog skin of *Amolops loloensis* in the Yunnan Province of China. Since then, *ca.* twenty new cathelicidins have been identified from only *ca.* ten amphibian species [25,27,30,31,32,33,34,35,36,37,38,39], despite 8516 amphibian species reported on AmphibiaWeb Database Search, thus highlighting our relatively poor understanding of both the ecological role and medicinal potential of these amphibian-derived AMPs. Existing structure-activity relationship (SAR) studies of mammalian-derived cathelicidins indicate that the highly cationic *N*-terminal region is essential for antimicrobial activity and a minimum of 16–18 residues are required [40,41,42]. Similar observations have been made among the comparatively fewer amphibian-derived cathelicidins studied [29,30,31]. Given amphibian-derived cathelicidins still remain minimally explored, we were interested in leveraging chemical synthesis to probe the SAR of a representative example and examine its potential for human antimicrobial therapy. We settled on Cathelicidin-PY, which was identified and isolated from skin secretions from the frog *Paa yunnanensis* and reported in 2013 by Wei et al. [33]. This cathelicidin shares a highly conserved cleavage site for maturation and a conserved disulfide bridge with a number of other amphibian-derived cathelicidins, such as OL-CATH-2 in particular (Figure 1) [30,31,39,43]. Cathelicidin-PY exhibits potent antibacterial activity towards both Gram-positive (*Staphylococcus aureus*; MIC = 2.74 μM) and Gram-negative (*Escherichia coli*; MIC = 1.37 μM and *Pseudomonas aeruginosa*; MIC = 5.48 μM) bacteria, as well as antifungal properties (*Candida albicans*; MIC = 1.37 μM) [33]. In addition to these potent activities, cathelicidin-PY also exhibits minimal cytotoxicity when tested up to 200 μg/mL towards murine macrophage cell lines (RAW 264.7 cell line) [33]. Cathelicidin-PY is a 29-amino acid residue sequence with a net charge of +6 (pH = 7.0) and a single disulfide bridge between Cys^3^ and Cys^7^. We set out to develop an efficient chemical synthesis of cathelicidin-PY that would enable facile access to analogues probing the disulfide bridge. We hypothesised that this bridge would be requisite for the observed biological activities; nonetheless, the 29-residue peptide bearing only a single disulfide would likely be highly susceptible to proteolytic degradation. We envisioned that chemical manipulation of cyclic bridges may be well tolerated but offer enhanced stability or therapeutic properties.

**Figure 2:**
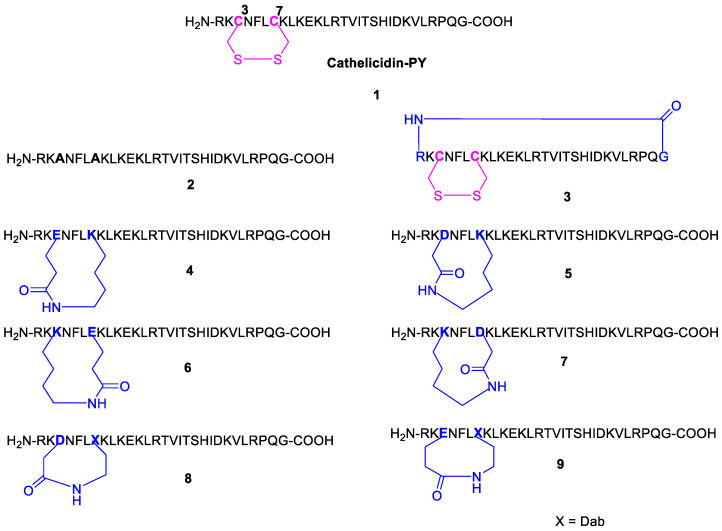
Structure of the cathelicidin-PY peptide, **1**, and a series of analogues, **2–9** of cathelicidin-PY. Dab = 2,4-diaminobutyric acid.

Herein we report an optimised synthetic method to prepare cathelicidin-PY, implementing on-resin assembly of the linear cathelicidin-PY sequence by rapid flow Fmoc-Solid Phase Peptide Synthesis (Fmoc-SPPS) and iodine-mediated disulfide bridge formation. The optimised route was then adapted to prepare a series of analogues probing the importance of the *N*-terminally located disulfide bridge and the potential for modification or inclusion of additional cyclic bridges (Figure 2). The cathelicidin-PY analogues were then subjected to antimicrobial evaluation, with compounds of interest further studied for stability in human plasma and their structural deviation evaluated by circular dichroism (CD).

## 2. Results and Discussion

### 2.1. Total Synthesis of Cathelicidin-PY

**Scheme 1:**
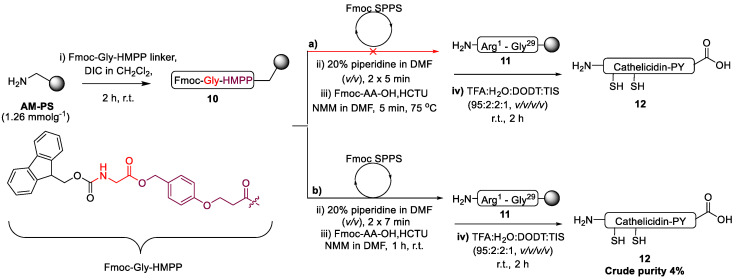
Synthesis of linear cathelicidin-PY peptide using (**a**) Microwave irradiation (**b**) Tribute automated synthesiser; AM-PS-aminomethyl polystyrene resin, DIC-*N,N’*-diisopropylcarbodiimide, HCTU-O-(1H-6-chlorobenzotriazole-1-yl)-1,1,3,3-tetramethyluronium hexafluorophosphate, HMPP linker-3-(4-hydroxymethylphenoxy)propionic acid, NMM-*N*-methylmorpholine, TFA-trifluoroacetic acid, DODT-2,2-(ethylenedioxy)diethanethiol, TIS-triisopropylsilane.

The total synthesis of cathelicidin-PY is yet to be detailed; therefore, a feasible and efficient synthetic route was developed by implementing Fmoc-SPPS. Microwave irradiation is known to be highly efficient in preparing larger or so-called “difficult” peptides [44,45,46,47]. Thus, expecting a problem-free synthesis, the preparation of cathelicidin-PY (**1**) was first attempted by Fmoc-SPPS under microwave irradiation (75 °C temp) with low-cost aminomethyl polystyrene (AM-PS) resin, with a substitution rate of 1.26 mmolg^−1^ and implementing *O*-(1*H*-6-chlorobenzotriazole-1-yl)-1,1,3,3-tetramethyluronium hexafluorophosphate (HCTU) as the coupling reagent (Scheme 1a).

**Figure 3:**
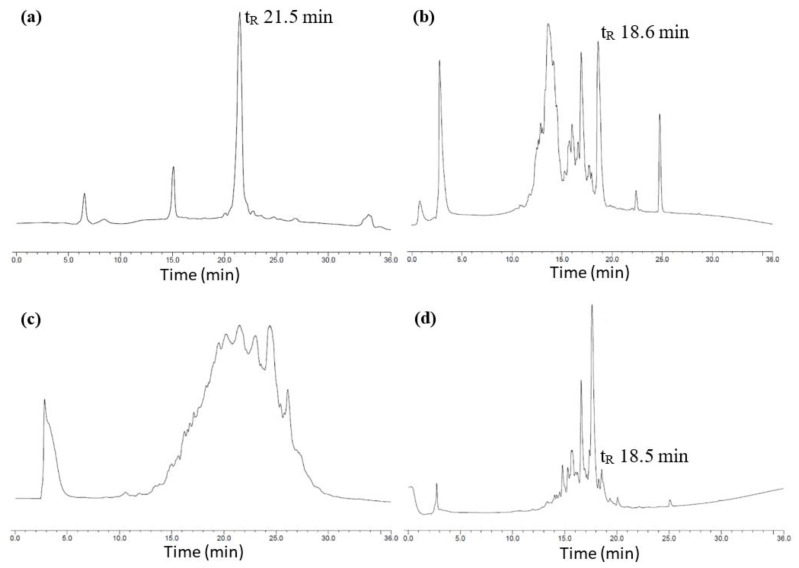
RP-HPLC chromatograms (214 nm) monitoring key intermediates during on-resin preparation of linear cathelicidin-PY (**12**) under microwave irradiation according to Scheme 1a. (**a**) confirms the desired product Fmoc-protected linear cathelicidin-PY^20−29^ (t_R_ = 21.5 min) (**b**) confirms the desired product Fmoc-protected linear cathelicidin-PY^10−29^ (t_R_ = 18.6 min) (**c**) the desired product, linear cathelicidin-PY (**12**), was not present (**d**) confirms the presence of the desired linear cathelicidin-PY (**12**) (t_R_ = 18.5 min) under automated synthetic conditions according to Scheme 1b.

Progression of the synthesis at cathelicidin-PY^20−29^ was monitored by cleavage of a small amount of peptidyl resin and determination of its purity by RP-HPLC (214 nm), which indicated the presence of the desired compound in satisfactory purity (82%; Figure 3a). However, upon continuing the synthesis to cathelicidin-PY^10−29^ and to completion, the RP-HPLC chromatogram became increasingly complex, failing to afford the final 29-mer (Figure 3b,c). The high substitution resin (1.26 mmolg^−1^) may have contributed to steric hinderance and intra- and intermolecular aggregation, ultimately resulting in the failed synthesis [48]. Somewhat surprised by this failure, we attempted automated synthesis at room temperature with increased coupling times from 5 min to 1 h and otherwise identical conditions (Scheme 1b). Pleasingly, analysis by cleavage of a small amount of peptidyl resin and determination of its purity by RP-HPLC (214 nm) revealed the successful assembly of the full linear sequence, albeit with an exceedingly poor overall purity of 4% (Figure 3d and Supplementary Materials Figure S2). The modest improvement, despite the reduced temperature, was possibly afforded by the increased coupling times throughout the synthesis.

**Scheme 2:**
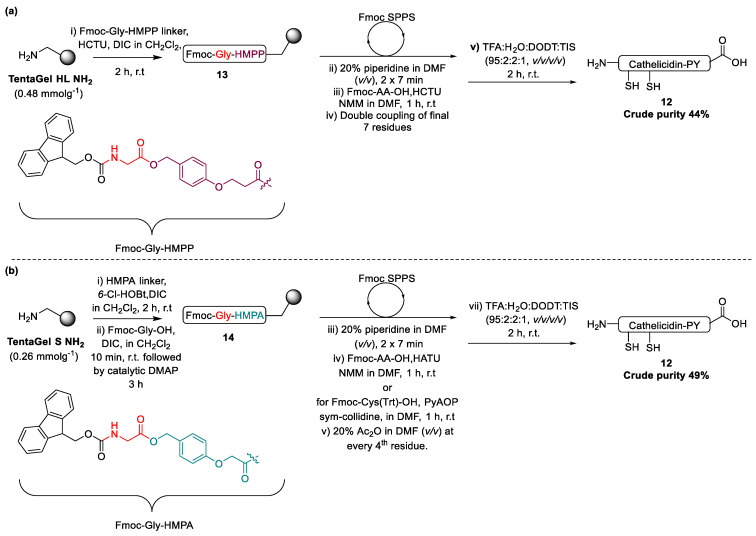
Synthesis of linear cathelicidin-PY on automated synthesiser using different resin substitutions (**a**) TentaGel HL NH_2_ (0.48 mmolg^−1^) and (**b**) TentaGel S NH_2_ (0.26 mmolg^−1^); 6-Cl-HOBt-1-hydroxy-6-chloro-benzotriazole, DIC-*N,N’*-diisopropylcarbodiimide, DMAP-4-methyldiaminopyridine, DODT-2,2-(Ethylenedioxy)diethanethiol NMM-*N*-methylmorpholine, PyAOP-7-azabenzotriazol-1-yloxy)tripyrrolidinophosphonium hexafluorophosphate, TFA-trifluoroacetic acid, TIS-triisopropylsilane.

With the need to perform an additional synthetic step (disulfide cyclisation) and the desire to prepare multiple analogues, we were unsatisfied with this result. We speculated that the poor performance of both microwave and room temperature synthesis could be attributed to the high resin substitution rate of the relatively poor swelling 1% DVB cross-linked PS resin [48]. Thus, AM-PS (1.26 mmolg^−1^) resin was substituted for polyethylene glycol (PEG) grafted resin, TentaGel HL NH_2_ resin, which has greater swelling capacity and is loaded at a lower substitution rate (0.48 mmolg^−1^). Repeating the synthesis at room temperature with this resin and double coupling of the final seven residues (Scheme 2a) afforded a remarkably improved crude purity (44%) for **12** (Supplementary Materials Figure S3). Pleased with this improvement, we sought to further optimise the preparation by implementing TentaGel S NH_2_ resin (Scheme 2b) with a further reduced substitution rate (0.26 mmolg^−1^). Furthermore, both Cys^3^ and Cys^7^ were manually coupled with PyAOP and the sterically hindered weak organic base, *sym*-collidine, to minimise epimerisation [49,50]. To further streamline the synthesis, and assuming the lower resin substitution would minimise aggregation, double couplings were abandoned for the final seven residues. To determine the effectiveness of this newly optimised synthetic protocol, a small sample of the peptidyl resin was cleaved upon completion of the synthesis and analysed by RP-HPLC, which indicated further improvement in the synthesis, affording the full linear peptide **12** in crude purity of 49% (Supplementary Materials Figure S4).

**Scheme 3:**
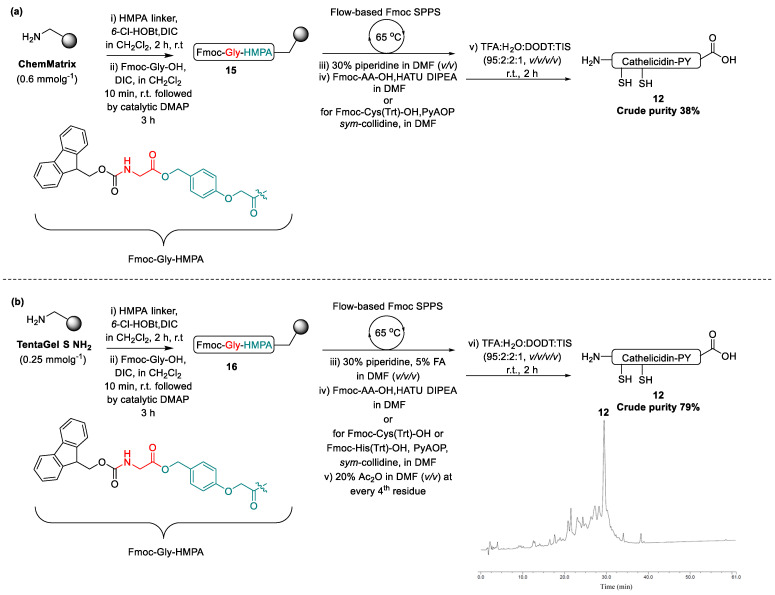
Synthesis of linear cathelicidin-PY on continuous flow synthesis at 65 °C with a flow rate of 15 mL/min and a resonance time of 40 s using different resin substitutions (**a**) ChemMatrix (0.6 mmolg^−1^) and (**b**) TentaGel S NH_2_ (0.25 mmolg^−1^); 6-Cl-HOBt-1-hydroxy-6-chloro-benzotriazole, HATU-hexafluorophosphate azabenzotriazole tetramethyl uronium, DIC-*N,N’*-diisopropylcarbodiimide, DMAP-4-methyldiaminopyridine, DODT-2,2 - (ethylenedioxy)diethanethiol, NMM-*N*-methylmorpholine, PyAOP-7-azabenzotriazol-1-yloxy)tripyrrolidinophosphonium hexafluorophosphate, FA-formic acid, TFA-trifluoroacetic acid, TIS-triisopropylsilane.

**Scheme 4:**
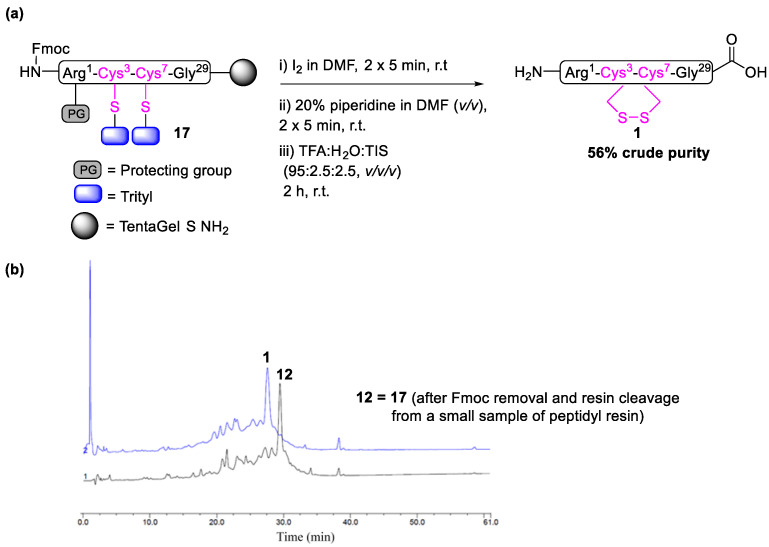
(**a**) On-resin iodine-mediated disulfide bond formation to afford cathelicidin-PY (**1**) prepared by flow-based Fmoc-SPPS in accordance with Scheme 3b; (**b**) RP-HPLC (214 nm) reaction monitoring.

With the recent addition of fast-flow SPPS to our laboratory [49], we wished to compare our optimised room-temperature synthesis protocol to flow conditions at 65 °C. The continuous flow approach to Fmoc-SPPS has offered significant advancement in the field, expediting peptide synthesis, whereby the coupling time is reduced to mere seconds [51,52,53]. However, reports of flow-chemistry preparation of disulfide-bridged peptides remain minimal. We recently demonstrated the applicability of flow chemistry towards the synthesis of capitellacin, a two disulfide containing AMP, finding that using altered cysteine coupling conditions and on-resin iodine-mediated orthogonal disulfide formation could rapidly yield the desired bicyclic peptide in respectable overall yield and purity [49]. Accordingly, we were eager to further explore the generality of this preparation by applying it to cathelicidin-PY [54]. In our first attempt, linear cathelicidin-PY was prepared on ChemMatrix resin, and a small-scale resin cleavage was carried out to analyse the product by RP-HPLC and ESI-MS. As envisioned, linear peptide **12** was successfully afforded, albeit at a crude purity of only 38% (Supplementary Materials Figure S6). Somewhat disappointingly, in comparison to room temperature, batch-wise coupling cycles (Scheme 2b) and flow chemistry synthesis (as per Scheme 3a) failed to provide a superior result. At this point, it was decided to review conditions such as the Fmoc removal solution and the coupling conditions. In accordance with our previously optimised protocol for epimerisation-prone cysteine residues, modified coupling conditions were also applied to His^20^ [50,55]. Lower-substituted TentaGel S NH_2_ resin (0.25 mmolg^−1^) was also implemented in place of ChemMatrix (0.6 mmolg^−1^). In addition, an acetic anhydride capping process was introduced after each fourth residue coupling to mask any unreacted amino groups. These slight adjustments (Scheme 3b), in line with our recent work [49], afforded a marked improvement, with **12** affording a crude purity of 79% (Supplementary Materials Figure S8), after having removed the terminal Fmoc-protection and cleaving a small portion from the resulting resin **17** (Scheme 4).

With resin-bound peptide **17** in hand (Scheme 4), the maximally protected linear peptide was subjected to iodine (3 equiv.) mediated simultaneous deprotection and disulfide formation between trityl-protected Cys^3^ and Cys^7^ residues in DMF for 2 × 5 min. The completion of the disulfide bridge formation was monitored by cleaving a small portion of the peptidyl resin and analysing it with RP-HPLC and ESI-MS, which eluded to the presence of the desired disulfide cyclised peptide **1**, which was clearly characterised by a distinct shift in retention time from the linear precursor **12** to the cyclised product **1** (t_R_ 29.4 min to 27.6 min, Scheme 4 or see Supplementary Materials Figure S11) and further confirmed by high resolution mass spectrometry (HRMS) (Supplementary Materials Figure S13). Final resin cleavage and global deprotection afforded crude peptide **1** with a satisfactory purity of 55.6% (Supplementary Materials Figure S11) and an overall yield of 28.6% (based on initial resin loading) after purification by RP-HPLC.

### 2.2. Design and Synthesis of Cathelicidin-PY Analogues

**Scheme 5:**
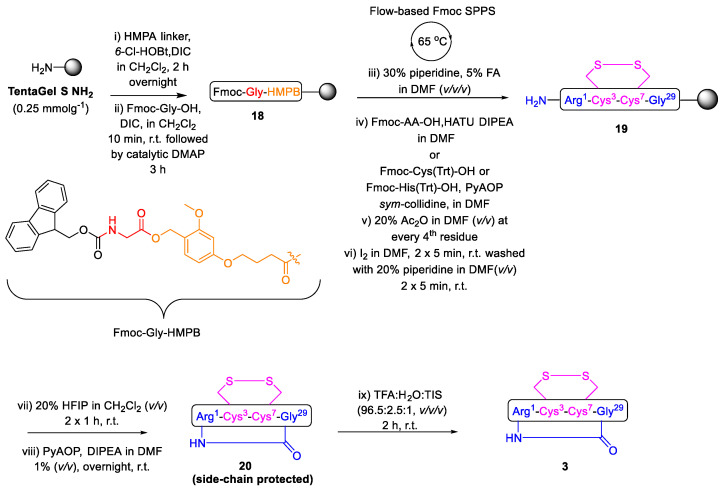
Synthesis of bicyclic cathelicidin-PY analogue **3** on continuous flow synthesis at 65 °C with a flow rate of 15 mL/min and a resonance time of 40 s. 6-Cl-HOBt-1-hydroxy-6-chloro-benzotriazole, DIC-*N,N’*-diisopropylcarbodiimide, DIPEA-*N,N*-diisopropylethylamine, DMAP-4-dimethylaminopyridine, FA-formic acid, HATU-hexafluorophosphate azabenzotriazole tetramethyl uronium, HFIP-1,1,1,3,3,3, hexafluoro-2-propanol, NMM-*N*-methylmorpholine, PyAOP-7-azabenzotriazol-1-yloxy)tripyrrolidinophosphonium, hexafluorophosphate, TFA-trifluoroacetic acid, TIS-triisopropylsilane.

With the successfully optimised protocol in hand, these conditions were adapted to prepare a linearised analogue with alanine substituted for Cys^3^ and Cys^7^ (**2**) and a further constrained head-to-tail bicyclised peptide (**3**). Accordingly, linear crude peptide **2** was afforded a respectable crude purity (51.6%), (Supplementary Materials Figure S15). With the NMR structure calculations of Lai and co-workers in hand [33], we envisioned that head-to-tail cyclisation of cathelicidin-PY could offer a favourable rigidifying effect to its folded structure. While the respective termini of the peptide are in relatively close proximity to one another to facilitate this, the terminal regions also appeared to possess the greatest RMSD, or degree of flexibility compared to the rest of the more well-defined peptide structure. To prepare bicyclic peptide **3**, we adapted our synthesis to incorporate a highly acid sensitive 4-(4-hydroxymethyl-3-methoxyphenoxy) butyric acid (HMPB) linker (Scheme 5). Under otherwise analogous conditions to peptide **1**, the linear peptide was prepared on-resin, and disulfide cyclisation between Cys^3^ and Cys^7^ was effected with iodine (3 equiv.) in DMF prior to liberating the fully protected peptide from the resin with 20% HFIP in CH_2_Cl_2_ (*v*/*v*). The fully side-chain-protected linear precursor was then cyclised under high dilution conditions (0.5 mM) in DMF, effected by PyAOP (5 equiv.) and DIPEA (1% *v*/*v* ) [56].

**Scheme 6:**
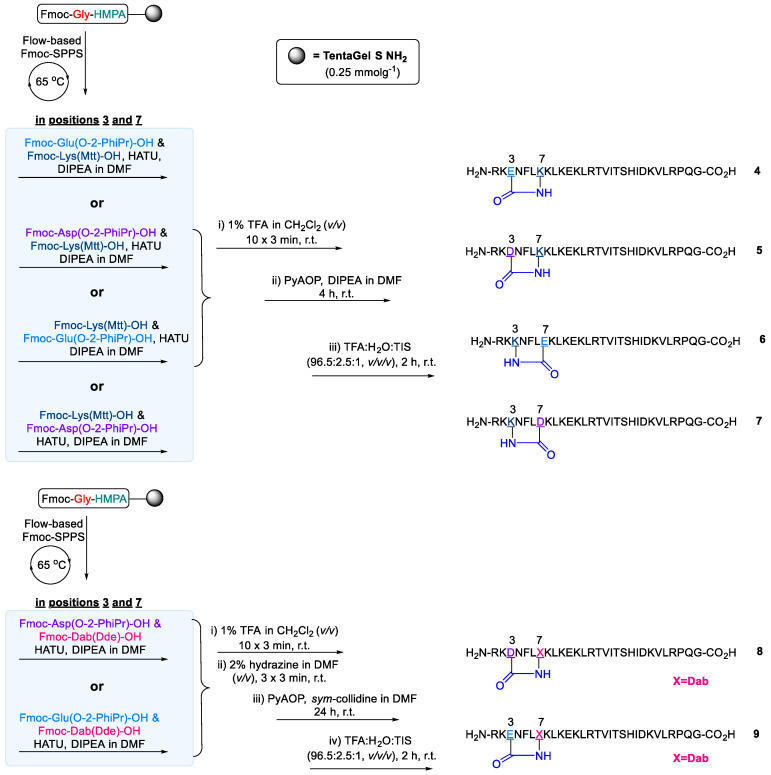
Synthesis of cathelicidin-PY peptidomimetics (**4–9**) at 65 °C with a flow rate of 15 mL/min and a resonance time of 40 s; 6-Cl-HOBt-1-hydroxy-6-chloro-benzotriazole, Dab = 2,4-diaminobutyric acid, Dde-1- (4,4-dimethyl-2,6-dioxocyclohex-1-ylidene)ethyl, DIC-*N,N’*-diisopropylcarbodiimide, DIPEA-*N,N*-diisopropylethylamine, DMAP-4-methyldiaminopyridine, FA-formic acid, HATU-hexafluorophosphate azabenzotriazole tetramethyl uronium, NMM-*N*-methylmorpholine, Mtt-4-methyltrityl, *O*-2-PhiPr-2-phenylisopropyl, PyAOP-7-azabenzotriazol-1-yloxy)tripyrrolidinophosphonium hexafluorophosphate, TFA-trifluoroacetic acid, TIS-triisopropylsilane.

Curious to probe the disulfide further, we envisaged that a more rigid lactam bridge may offer improved antimicrobial activity and plasma stability. We believed this would additionally offer interesting insights into the structure-activity relationship with respect to tolerances of the *N*-terminal cyclic loop of cathelicidin-PY and likely related amphibian AMPs [57,58,59]. Accordingly, a series of lactam rings containing mimetics (**4–9**) were prepared (Scheme 6). Implementing Fmoc-Glu(*O*-2-Ph*i*Pr)-OH or Fmoc-Asp(*O*-2-Ph*i*Pr)-OH and Fmoc-Lys(Mtt)-OH in position of Cys^3^ and Cys^7^, as required, enabled an on-resin macrolactamisation of various ring size analogues (peptides **4–7**) upon orthogonal deprotection by 1% *v*/*v* TFA in CH_2_Cl_2_, followed by immediate on-resin macrocyclisation mediated by PyAOP and DIPEA in DMF for 4 h, removal of the terminal *N*^α^-Fmoc protection and resin cleavage. Guided by preliminary assay results (vide infra), further ring contractions were enabled by implementing Fmoc-2,4-diaminobutyric acid (Dab) in place of lysine to prepare macrocyclic peptides **8** and **9**. With Fmoc-Dab(Dde)-OH commercially available, the *N*^α^-Fmoc group of the terminal Arg residue was exchanged on-resin for a Boc group, thus accommodating on-resin removal of the Dde protecting group by 2% *v*/*v* hydrazine in DMF (2 × 4 min), without unmasking the terminal *N*^α^-amino group [60]. With cyclisation requiring up to 24 h for completion for the more constrained lactams (**8** and **9**), DIPEA was substituted for *sym*-collidine during on-resin macrocyclisation. Upon global deprotection and liberation from the respective resins with TFA, lactam-bridged peptides **4–9** were afforded in satisfactory crude purity (ranging 44–54%) and purified to homogeneity by RP-HPLC.

### 2.3. Circular Dichroism Analysis

**Figure 4:**
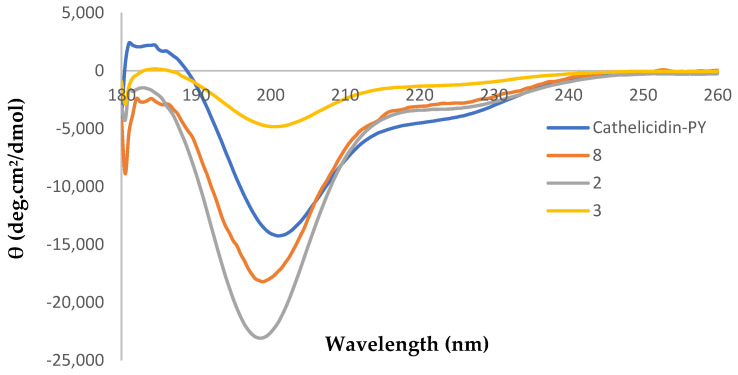
Circular dichroism (CD) spectra carried out in water for cathelicidin−PY and associated analogues, **2**, **3**, and **8**, depicting a random coil conformation. CD spectra were collected at 25 °C with peptides dissolved at 0.2 mM in pH 7.4 phosphate buffer (20 mM).

**Figure 5:**
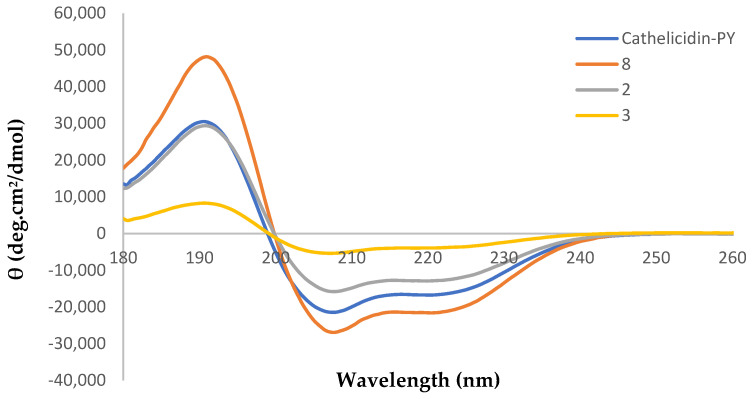
Circular dichroism (CD) spectra carried out in membrane-mimetic environments (TFE:H_2_O, 1:1, *v*/*v*) for cathelicidin−PY and associated analogues, **2**, **3**, and **8**, depicting an α−helical secondary structure. CD spectra were collected at 25 °C with peptides dissolved at 0.2 mM.

Circular dichroism (CD) experiments were used to estimate the secondary structural elements of cathelicidin-PY (**1**) and selected analogues, **2**, **3**, and **8** (selected based on bioactivity, vide infra). These peptide samples were prepared in a pH 7.4 phosphate buffer and a membrane-mimetic environment (TFE: H_2_O, 1:1, *v*/*v*) at a concentration of 0.2 mM. The CD spectra of cathelicidin-PY (**1**) demonstrated a distinct negative maxima at ~205 nm in H_2_O (Figure 4), indicating cathelicidin-PY (**1**) adopted a mostly random-coil conformation, in accordance with the original report upon its isolation [33]. The spectra in TFE:H_2_O (1:1, *v*/*v*) solution were also in good agreement, with negative maxima observed at *ca.* 208 and 222 nm, indicating α-helical secondary structure in the membrane mimetic solvent system (Figure 5).

Pleasingly, CD evaluations of peptide **8** indicated that disulfide substitution for a lactam bridge is well tolerated, with similar spectra to the native peptide **1** observed in both solvent systems, including a slightly more pronounced α-helical character in TFE:H_2_O (1:1). To our surprise, linear peptide **2**, in which the disulfide is abolished, retains similar structural properties; despite demonstrating a slightly greater degree of random coil in H_2_O, it only demonstrated a modestly reduced α-helical character in TFE:H_2_O (1:1). On the other hand, the head-to-tail bicyclised analogue **3** demonstrated a remarkably different CD spectra, with only a weak negative maxima evident at ~205 nm in H_2_O and strikingly reduced helical character in TFE:H_2_O (1:1, *v*/*v*).

### 2.4. Antimicrobial Activity

As we were primarily interested in the clinical potential of cathelicidin-PY (**1**) and its analogues **2**–**9**, cation-adjusted Mueller-Hinton broth (CAMHB) was implemented during our in vitro antimicrobial screening towards bacteria. However, non-cation adjusted Mueller Hinton broth (MHB) was also implemented to evaluate the cation sensitivity of these AMPs. In each medium, compounds **1**–**9** were subjected to minimum inhibitory concentration (MIC) assays with *E. coli* (ATCC 25922), *P. aeruginosa* (SVB-B9), and *S. aureus* (ATCC 29213), in accordance with CLSI microbroth dilution protocols (Table 1) [61]. It is noteworthy, however, that the initial report of cathelicidin-PY’s isolation documented MIC assays performed in Luria Broth (LB). Furthermore, antifungal activity towards *C. albicans* was investigated [62].

Towards *E. coli* native cathelicidin-PY (**1**) maintained high potency in CAMHB media with an MIC of 2 μM, only two-fold greater than its MIC in non-adjusted MHB and in accordance with the reported activity of the isolated compound [33].

To our surprise, the activity of the alanine-substituted linear analogue (**2**) was only two-fold diminished, suggesting the native disulfide bond is non-essential to both secondary structure (as judged by CD) and biological activity. On the other hand, imparting further conformational restriction to bicyclic analogue (**3**) resulted in an eight-fold reduction in activity, which is consistent with the loss in secondary structure observed by CD spectroscopy. Pleasingly, three lactam-bridged analogues (**4**, **6**, and **8**) demonstrated activity within two-fold of the native compound, including the most conformationally restricted analogue (**8**), in which the bridge length is only increased (relative to the native) by one atom but further rigidified by the non-rotatable peptide bond. Interestingly, analogues **4** and **6** were two-fold more active than their transposed counterparts **5** and **7**, respectively. In non-cation-adjusted MHB media, the activity trend remained similar; however, all compounds **1**–**9** were two- to four-fold more active towards *E. coli*. The consistent cation-dependent activity profile suggests analogues **2**–**9** likely share a common membrane lytic mechanism of action, in accordance with the native cathelicidin-PY (**1**) [33].

With reference to *E. coli*, Cathelicidin-PY (**1**) and analogues **2**–**9** were dramatically less active (eight-fold reduction) towards *P. aeruginosa* in CAMHB media, with the native peptide demonstrating an MIC of 16 μM and only analogues **2** and **8** demonstrating any noticeable antimicrobial activity with MIC values of 32 μM. The reduced activity of **1** towards *P. aeruginosa* is akin to the isolation report, whereby an approx. five-fold reduction in activity was observed with respect to *E. coli*. However, we found activity of these AMPs towards *P. aeruginosa* was quite dramatically restored in non-cation adjusted MHB, with the native peptide (**1**) demonstrating an MIC of 2 μM and analogues **2**, **6** and **8** demonstrating MIC values of 4–8 μM.

To our surprise, we were unable to replicate the Gram-positive antibacterial or antifungal activity that was reported for **1** upon its isolation when performing our assays in CAMHB and RPMI 1640 media, respectively. With increasing recognition for the importance of the microbiome in human health [63,64], there is a need for new generation antimicrobials with improved selectivity and reduced capacity to induce drug resistance in non-target organisms [65,66,67]. In this respect, the selective and potent activity of **1** and selected lactam bridged analogues (**4**, **6**, and **8,** MIC = 4 μM) towards *E. coli*, in the presence of physiologically relevant levels of divalent cations is both intriguing and exciting. The two-fold increased selectivity of **4** and **6** amongst the tested Gram-negative species is particularly promising.

### 2.5. Peptide Stability

The proteolytic stability of cathelicidin-PY (**1**) and analogues of interest (**2** and **8**) was determined by incubation with human serum and analysis by RP-HPLC (Table 2). Due to the loss of both secondary structure and bioactivity, the stability of bicyclic analogue (**3**) was not investigated. Despite the presence of the disulfide bonds Cys^3^ and Cys^7^ in the *N*-terminal region of cathelicidin-PY (**1**), the peptide was found to degrade rapidly, with only *ca.* 10% remaining intact after 3 h and near total degradation observed within 6 h. Somewhat surprisingly, the linearised analogue (**2**), in which the disulfide-forming Cys residues were substituted for alanine, was found to be marginally more stable towards human serum than the parent peptide (**1**). The most highly constrained of the lactamised analogues was investigated for stability. Lactam bridged analogue **8** demonstrated a somewhat modest improvement in stability, with near total degradation observed within 6 h and only *ca.* 13% remaining intact after 3 h. The biggest improvement in stability was observed within just the first hour of exposure to serum, where over 60% remained intact, compared with just *ca.* 20% for the native peptide (**1**).

## 3. Conclusions

We have successfully optimised an all-on-resin synthetic strategy to prepare cathelicidin-PY (**1**) by rapid flow Fmoc-SPPS chemistry, paired with an on-resin iodine-mediated disulfide bridge formation. Furthermore, the benefit of modified coupling conditions for racemisation prone residues (Cys/His) under high temperature flow Fmoc-SPPS was highlighted. The optimised methodology was adopted in preparing a library of eight analogues. Surprisingly, alanine substitution of the Cys^3^ to Cys^7^ disulfide in linearised analogue **2** had only a modest impact upon secondary structure, biological activity, and serum stability, suggesting that disulfide is non-essential and also amenable to chemical modification. This led to the design of lactamised analogues **4**–**9** with varying bridge lengths. On the other hand, we rationalised that a more constrained head-to-tail bicyclised analogue (**3**) may offer benefits towards both activity and peptide stability based on the calculated 3D structure of the native peptide. To our surprise, this bicyclisation abolished both bioactivity and secondary structure with respect to the native peptide, suggesting the secondary structure and bioactivity are closely linked. Selected lactam bridged analogues, however, remained similarly potent towards Gram-negative pathogens, retained similar secondary structure, and yielded a modest improvement in serum stability. Like the native compound (**1**), all synthetic derivatives also failed to demonstrate antimicrobial activity towards the Gram-positive pathogen *S. aureus* and the fungal pathogen *C. albican,* contrary to the initial report of cathelicidin-PY (**1**). In the presence of physiologically relevant levels of divalent cations, cathelicidin-PY (**1**) and select lactam bridged analogues (**4**, **6**, and **8**) demonstrated selective potency towards *E. coli*. Such selectivity is encouraging, given the growing need for selective antimicrobials that can both preserve the microbiome and avoid resistance development in off-target species.

## 4. Materials and Methods

### 4.1. General Procedures and Reagents

All reagents were purchased as reagent grade and used without further purification. Solvents for peptide synthesis and RP-HPLC were purchased as synthesis-grade and HPLC-grade, respectively.

Polystyrene AM NH_2_ (AM PS), TentaGel^®^-S-NH_2_ resin with alternative particle size of 90 μm (used in automated peptide synthesiser) and 130 μm (used in flow chemistry vessel) were purchased from RAPP polymere (Tubingen, Germany). ChemMatrix^®^ resin and Fmoc-Gly-3-(4-hydroxymethylphenoxy)propionic acid (Fmoc-Gly HMPP linker) were purchased from Polypeptide Laboratories Group (Limhamn, Sweden). 4-(Hydroxymethyl)phenoxyacetic acid (HMPA), 4-(4-hydroxymethyl-3-methoxyphenox)butyric acid (HMPB), 4-(Dimethylamino)pyridine (DMAP), Fmoc-Lys(Mtt)-OH, 1,1,1,3,3,3-hexafluoro-2-propanol (HFIP), acetic acid (Ac_2_O) and polymyxin B sulfate were purchased from AK Scientific (Union City, California). Amphotericin B (from *Streptomyces)*, human serum (from male AB serum), *N*,*N*-diisopropylethylamine (DIPEA), Fmoc-Dab(Dde)-OH, 2,4,6-trimethylpyridine (*sym*-collidine), *N,N’*-diisopropylcarbodiimide (DIC), triisopropylsilane (TIPS), piperidine, 3,6-dioxa-1,8-octanedithiol (DODT), trichloroacetic acid (TCA) and formic acid (FA) were purchased from Sigma-Aldrich (St Louis, MO, USA). Diethyl ether (Et_2_O) was purchased from Avantor Performance Materials (Center Valley, USA). Trifluoroacetic acid (TFA) was purchased from Oakwood Chemicals (Estill, SC, USA). *N*-Methylmorpholine (NMM), 1-[bis(dimethylamino)methylene]-1H-1,2,3-triazolo [4,5-b]pyridinium 3-oxide hexafluorophosphate (HATU) and all Fmoc-amino acids were purchased from CS Bio China (Shanghai, China). Fmoc-Asp(*O*-2-PhiPr)-OH and Fmoc-Glu(*O*-2-PhiPr)-OH, 6-chloro-1-hydroxybenzotriazole (6-Cl-HOBt), Fmoc-Ser(tBu)-Thr(Psi(Me,Me)pro)-OH was purchased from Apptec (Louisville, Kentucky, USA). *N,N*-dimethylformamide (DMF; AR grade) and acetonitrile (CH_3_CN, HPLC grade) were purchased from Thermo Scientific (Hampshire, NH, USA). Dichloromethane (CH_2_Cl_2_) and iodine (I_2_) were purchased from ECP Limited (Auckland, New Zealand). Polypropylene 96-well flat bottom plates were purchased from Greiner Bio-One (Kremsmünster, Austria). Milli-Q high purity deionised water (MQ H_2_O) was available from a Sartorius Arium^®^ Pro Ultrapure Water System from Sartorius Stedim Biotech (Gottingen, Germany).

Microwave reactions were carried out in a Biotage^®^ (Uppsala, Sweden) Initiator Alstra^TM^ microwave peptide synthesiser at variable temperatures. Room-temperature automated peptide synthesis was performed on a Tribute^®^ automated synthesiser at Gyros Protein Technologies (Tucson, Arizona) at room temperature. Manual flow chemistry was carried out in a custom-made metal reactor packed with the desired resin. Reagents were delivered via a 5-mL stainless steel heating loop submerged in a water bath (65 °C) to the reaction vessel, also submerged in a water bath (65 °C). All reagents were delivered at a constant flow rate of 15 mL/min.

Reverse phase high performance liquid chromatography (RP-HPLC) was performed on a Thermo Scientific (Waltham, MA, USA) Dionex Ultimate 3000 HPLC equipped with a four channel UV Detector at 210, 225, 254 and 280 nm using either an analytical column Waters (Milford, MA, USA) XTerra^®^ MS C18, (5 μM; 4.6 × 150 mm) at a flow rate of 1 mL min^−1^ or a semi-preparative column Phenomenex^®^ (Torrance, CA, USA), Gemini C18, 5 mm; 10 × 250 mm) at a flow rate of 4.5 mL min^−1^ operated at room temperature. For both analytical and semi-preparative RP-HPLC, the solvents used were as follows: solvent A = 0.1% TFA in water (MQ H_2_O) and solvent B = 0.1% TFA in MeCN. For analytical RP-HPLC, the gradient employed was 5–65% of solvent B over 60 min at a flow rate of 1 mL/min, unless specified otherwise.

High-resolution mass spectrometry (HRMS) was performed with a Bruker (Billerica, MA, USA) micrOTOFQ mass spectrometer by using electrospray ionization (ESI) in the positive mode at a nominal accelerating voltage of 70 eV.

Low-resolution mass spectrometry was performed on a Waters Quattro micro–API Mass Spectrometer in ESI positive mode.

LC-MS spectra were acquired using an Agilent Technologies (Santa Clara, CA, USA) 1260 Infinity LC equipped with an Agilent Technologies 6120 Quadrupole mass spectrometer. An analytical column (Agilent C3, 3.5 mm; 3.0 × 150 mm) was used at a flow rate of 0.3 mL min^−1^ using a linear gradient of 5% B to 95% B over 30 min, where solvent A was 0.1% formic acid in H_2_O and solvent B was 0.1% formic acid in acetonitrile.

### 4.2. Peptide Synthesis

Peptides were prepared according to Fmoc-SPPS protocols by automated peptide synthesisers or flow apparatus. For further detail and experimental data, please see the Electronic Supporting Information file.

### 4.3. Circular Dichroism (CD) Spectroscopy

All CD spectra were recorded using a Chirascan CD spectrometer (Applied Photophysics Ltd., Leatherhead, UK) at 25 °C with a cuvette of 1 mm path length (106-QS, Hellma Analytics, Mullheim, Germany) in the range from 180 and 260 nm at 0.5 nm intervals with a time-to-point of 0.5 s. Each peptide sample was prepared to a concentration of 0.2 mM in the respective solvents. The spectra used an average of five scans obtained with a 1 nm optical bandwidth. The baseline scans were collected with the solvent alone, averaged, and then subtracted from the sample scans. Raw data was exported to excel for processing and expressed as mean residue molar ellipticities [θ] in (deg.cm^2^/dmol).

### 4.4. Bacterial Minimum Inhibitory Concentration Assay

*Staphylococcus aureus* ATCC 29213, *Pseudomonas aeruginosa* (SVB-B9) (type strain), and *Escherichia coli* ATCC 25,922 were grown in either non-cation-adjusted or cation-adjusted Mueller Hinton (MH) broth at 37 °C with shaking (200 rpm). MIC assays were performed in technical triplicate in accordance with the CLSI-recommended protocol [61]. All assays were performed independently on three occasions, with the reported MIC defined as the lowest concentration for which agreement was observed consistently for all replicates.

### 4.5. Fungal Minimum Inhibitory Concentration Assay

Antimicrobial susceptibility of *Candida albicans* SC5314 (type strain) was assessed by broth microdilution in RPMI 1640 media in accordance with the CLSI recommended protocols [62]. All assays were performed independently on three occasions, with the reported MIC defined as the lowest concentration for which agreement was observed consistently for all replicates.

### 4.6. Human Serum Stability Assay

Peptides were initially dissolved in MQ H_2_O at 10 mM. These solutions were further diluted 1:3 in MQ H_2_O to achieve a final concentration of 2.5 mM and placed in an incubator at 37 °C. A solution of human serum and 100 mM phosphate buffer solution (pH 7.45) was placed at 37 °C for 20 min. Once both solutions had equilibrated at 37 °C, phosphate buffer (500 μL) and human serum (500 μL) were combined to make a final volume of 1 mL and centrifuged at 12,500 rpm for 10 min. The resulting supernatant (900 μL) was removed and warmed to 37 °C for 10 min. Once both peptide and serum supernatants were equilibrated to 37 °C, the peptide was added to the serum supernatant (1:9) to reach a final peptide incubation concentration of 250 μM in the phosphate buffer: human serum solution (1:1). Following addition of the peptide solution to the serum, the solution was immediately mixed/inverted, and an aliquot (75 μL) removed at t_0_ timepoint. The remaining serum/peptide solution was incubated under agitation at 37 °C, and aliquots were taken at 10 min, 1 h, 3 h, and 6 h. Aliquots were immediately added to an equal volume (75 μL) of cold snap solution containing 6% TCA in 30% MeCN (*v*/*v*), which was pre-cooled to 4 °C. This solution was allowed to cool for a further 5 min prior to being centrifuged at 12,500 rpm for 10 min, and the supernatant (100 μL) carefully removed and analysed by RP-HPLC (214 nm). Peak areas were normalised to a t_0_ aliquot.

## Figures and Tables

**Table 1:** Minimal inhibitory concentrations (MICs) for synthetics (**1**) and analogues (**2**–**9**).

Microorganism		Minimum Inhibitory Concentration (μM)										
	Cation Adjusted Media	Compound										
		1	2	3	4	5	6	7	8	9	P *	A *
Gram-negative												
*E. coli* (ATCC 25922)	Y	2	4	16	4	8	4	8	4	8	1	-
	N	1	1	8	2	2	2	2	1	2	1	-
*P. aeruginosa* (SVB-B9) ^T^	Y	16	32	>64	>64	>64	64	>64	32	64		2
	N	2	4	32	16	16	8	16	8	16	2	-
Gram-positive												
*S. aureus* (ATCC 29213)	Y	>64	>64	>64	>64	>64	>64	>64	>64	>64	-	>64
	N	>64	>64	>64	>64	>64	>64	>64	>64	>64	-	>64
Fungi												
*C. albicans* (SC5314) ^T^	N/A	>64	>64	>64	>64	>64	>64	>64	>64	>64		1

* P = polymyxin, A = amphotericin B; ^T^ = Type strain.

**Table 2:** Stability assay of cathelicidin-PY, **1**, and analogues **2** and **8** in human serum at various time points as determined by RP-HPLC (214 nm).

	Time Point and % Sample Remaining			
Test Compound	t_10 min_	t_1 h_	t_3 h_	t_6 h_
Cathelicidin-PY	84.7%	20.8%	8.8%	2.4%
**2**	92.9%	61.6%	8.1%	7.4%
**8**	93.9%	64.6%	13.4%	10.0%

## Data Availability

All supporting data can be found in the Electronic Supporting Information file.

## Supplementary Materials

A full Electronic Supporting Information File can be downloaded at: https://www.mdpi.com/article/10.3390/molecules28041993/s1.
