# Supplementary material for: Flow-Based Fmoc-SPPS Preparation and SAR Study of Cathelicidin-PY Reveals Selective Antimicrobial Activity
Source: Molecules. 2023 Feb 20;28(4):1993. doi: 10.3390/molecules28041993 (PMC9959817; doi:10.3390/molecules28041993)
Supplement: Supplementary file 1 [file molecules-28-01993-s001.zip › molecules-2220396-supplementary/molecules-2220396-supplementary.pdf]

Supporting Information

Flow-Based Fmoc-SPPS Preparation and SAR Study of Cathelicidin-PY Reveals Selective Antimicrobial Activity

Shama Dissanayake[1], Junming He[1], Sung H. Yang[1], Margaret A. Brimble[1-3], Paul W. R. Harris[1,2] and Alan J. Cameron[1,2]*

1 School of Chemical Sciences and School of Biological Sciences, The University of Auckland, 23 Symonds St, Auckland, 1142 New Zealand

2 Maurice Wilkins Centre for Molecular Biodiscovery, The University of Auckland, Auckland, 1142 New Zealand

* Correspondence: A. J. Cameron; alan.cameron@auckland.ac.nz

S.1 General Procedures and Reagents

All reagents were purchased as reagent grade and used without further purification. Solvents for peptide synthesis and RP-HPLC were purchased as synthesis grade and HPLC grade, respectively.

Polystyrene AM NH_2 (AM PS), TentaGel®-S-NH_2 resin with alternative particle size of 90 μm (used in automated peptide synthesiser) and 130 μm (used in flow chemistry vessel) were purchased from RAPP polymere (Tubingen, Germany). ChemMatrix® resin and Fmoc-Gly-3-(4-hydroxymethylphenoxy)propionic acid (Fmoc-Gly HMPP linker) were purchased from Polypeptide Laboratories Group (Limhamn, Sweden). 4-(Hydroxymethyl)phenoxyacetic acid (HMPA), 4-(4-hydroxymethyl-3-methoxyphenox)butyric acid (HMPB), 4-(dimethylamino)pyridine (DMAP), Fmoc-Lys(Mtt)-OH, 1,1,1,3,3,3-hexafluoro-2-propanol (HFIP), acetic acid (Ac_2O) and polymyxin B sulfate were purchased from AK Scientific (Union City, California). Amphotericin B (from *Streptomyces)*, human serum (from male AB serum), *N*,*N*-diisopropylethylamine (DIPEA), Fmoc-Dab(Dde)-OH, 2,4,6-trimethylpyridine (sym-collidine), *N,N'*-diisopropylcarbodiimide (DIC), triisopropylsilane (TIPS), piperidine, 3,6-dioxa-1,8-octanedithiol (DODT), trichloroacetic acid (TCA) and formic acid (FA) were purchased from Sigma–Aldrich (St Louis, MO, USA). Diethyl ether (Et_2O) was purchased from Avantor Performance Materials (Center Valley, USA). Trifluoroacetic acid (TFA) was purchased from Oakwood Chemicals (Estill, SC, USA). *N*-Methylmorpholine (NMM), 1-[bis(dimethylamino)methylene]-1H-1,2,3-triazolo[4,5-b]pyridinium 3-oxide hexafluorophosphate (HATU) and all Fmoc-amino acids were purchased from C S Bio China (Shanghai, China). Fmoc-Asp(*O*-2-PhiPr)-OH and Fmoc-Glu(*O*-2-PhiPr)-OH, 6-chloro-1-hydroxybenzotriazole (6-Cl-HOBt) was purchased from Apptec (Louisville, Kentucky, USA). *N,N*-dimethylformamide (DMF; AR grade) and acetonitrile (CH_3CN, HPLC grade) were purchased from Thermo Scientific (Hampshire, NH, USA). Dichloromethane (CH_2Cl_2) and iodine (I_2) were purchased from ECP Limited (Auckland, New Zealand). Polypropylene 96-well flat bottom plates were purchased from Greiner Bio-One (Kremsmünster, Austria). Milli-Q high purity deionized water (MQ H_2O) was available from a Sartorius Arium® Pro Ultrapure Water System from Sartorius Stedim Biotech (Gottingen, Germany).

Microwave reactions were carried out in a Biotage® Initiator Alstra™ microwave peptide synthesizer (Uppsala, Sweden) with variable temperatures. Room-temperature automated peptide synthesis was performed on a Tribute® automated synthesiser Gyros Protein

Technologies (Tucson, Arizona) under room temperature. Manual flow chemistry was carried out in a custom made metal reactor packed to 0.15 mmol scale of peptide synthesis. Reagents were delivered via a 5 mL stainless steel heating loop submerged in a water bath (65 ^{o}C) to the reaction vessel, also submerged in a water bath (65 ^{o}C). All reagents were delivered at constant flow rate of 15 ml/min.

Reverse phase high performance liquid chromatography (RP-HPLC) was performed on a Thermo Scientific (Waltham, MA, USA) Dionex Ultimate 3000 HPLC equipped with a four channel UV Detector at 210, 225, 254 and 280 nm using either an analytical column Waters (Milford, MA, USA) XTerra® MS C18, (5 μm; 4.6 × 150 mm) at a flow rate of 1 mL min^{-1} and a semi-preparative column Phenomenex® (Torrance, CA, USA), Gemini C18, 5 μm; 10 × 250 mm) at a flow rate of 4.5 mL min^{-1} operated at room temperature. For both analytical and semi-preparative RP-HPLC, solvents used were as follows: solvent A = 0.1% TFA in water (MQ H_2O) and solvent B = 0.1% TFA in MeCN. For analytical RP-HPLC the gradient employed was 5- 65% of solvent B over 60 minutes at flow rate 1 mL/min, unless specified otherwise.

High-resolution mass spectrometry (HRMS) was performed with a Bruker (Billerica, MA, USA) micrOTOFQ mass spectrometer by using electrospray ionisation (ESI) in the positive mode at a nominal accelerating voltage of 70 eV.

Low-resolution mass spectrometry was performed on a Waters Quattro micro–API Mass Spectrometer in ESI positive mode.

LC-MS spectra were acquired using Agilent Technologies (Santa Clara, CA, USA) 1260 Infinity LC equipped with an Agilent Technologies 6120 Quadrupole mass spectrometer. An analytical column (Agilent SB-C3, 3.5 μm; 3.0 × 150 mm) was used at a flow rate of 0.3 mL min^{-1} using a linear gradient of 5% B to 95% B over 30 min, where solvent A was 0.1% formic acid in H_2O and B was 0.1% formic acid in acetonitrile.

S.2 First synthetic approach towards the synthesis of cathelicidin-PY under microwave irradiation using AM-PS resin

Method 1: Loading Aminomethyl Polystyrene (AMPS) resin with Fmoc-Gly HMPP linker

To AM-PS resin (79.4 mg, 0.1 mmol, 1.26 mmolg^{-1}) pre-swollen in CH_2Cl_2 (5 mL, 10 min), was added a solution of Fmoc-Gly-HMPP linker (95 mg, 0.2 mmol, 2.0 equiv.) in CH_2Cl_2 (2 mL) followed by addition of DIC (31 μL, 0.2 mmol, 2.0 equiv.). The reaction was gently agitated at room temperature for 2 h. The resin was filtered and washed with CH_2Cl_2 (3 × 3 mL) and DMF (3 × 3 mL).

Method 2: General procedure for removal of N$^{\alpha}$-Fmoc protecting group

The peptidyl resin was treated with a solution of 20% *v/v* piperidine in DMF (4 mL) and the mixture agitated at room temperature for 5 min, filtered and repeated once for a further 15 mins. The resin was filtered and washed with DMF (3 × 3 mL).

Method 3: General procedure for amino acid coupling using HCTU

To the peptidyl resin (0.1 mmol) was added the appropriate amino acid, Fmoc-AA-OH (0.50 mmol, 5.0 equiv.) with HCTU (206.86 mg, 0.45 mmol, 4.5 equiv.) and NMM (109.94 μl, 0.1 mmol, 10 equiv.) in DMF. The reaction mixture was irradiated for 5 min at 75 ^{o}C, filtered and repeated again with fresh reagents. Fmoc-His-OH (309.85 mg, 0.50 mmol, 5.0 equiv.) and Fmoc-Arg(Pbf)-OH (324.4 mg, 0.50 mmol, 5.0 equiv.) were coupled for 25 minutes at room temperature and then repeated for 5 minutes at 72 ^{o}C with fresh reagents. Fmoc-Cys(Trt)-OH (292.85 mg, 0.50 mmol, 5.0 equiv.) was coupled for 15 minutes at room temperature and then repeated for 10 minutes at 47 ^{o}C at 110 W with fresh reagents.

Method 4: General procedure for capping the free amino groups:

N$^{\alpha}$-Fmoc protected peptidyl resin was treated with a solution of acetic anhydride and NMM in DMF (1 mL, 20:20:60 *v/v/v*) and the mixture was agitated for 2 min at 75 ^{o}C. The resin was filtered and washed with DMF (3 × 3 mL).

Method 5: TFA mediated resin cleavage and global deprotection

Peptidyl resin was treated with a mixture of TFA/ H_2O/DODT/TIS (95:2:2:1, 10 mL, *v/v/v*) for 120 min at room temperature. The filtrate was concentrated under a gentle stream of N_2 followed by the addition of cold diethyl ether to form a precipitate. The mixture was centrifuged, and the solution was carefully decanted off and discarded. The solid pellet was dissolved H_2O (20 mL) and lyophilised.

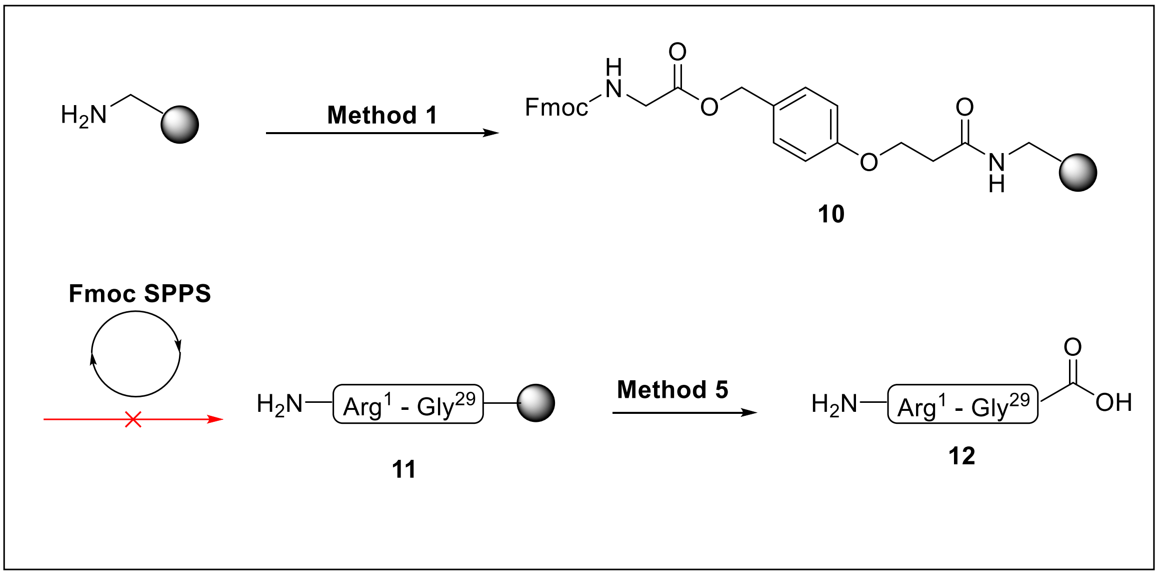

Scheme S1. First synthesis attempt of linear cathelicidin-PY peptide on AM-PS resin.

Fmoc-SPPS was used for the attempted synthesis of cathelicidin-PY employing aminomethyl polystyrene (AM-PS) resin, functionalizing with Fmoc-Gly-HMPP linker using **Method 1**. N^{α}-Fmoc protecting group was removed using **Method 2**. To resin bound NH_2-Gly-HMPP linker the appropriate amino acids were coupled using **Method 3**. Unreacted amino groups were capped using **Method 4**, followed by Fmoc-removal using **Method 2**. The resulting peptide was cleaved using **Method 5** to afford a white solid. LCMS analysis indicated that the desired product was not present.

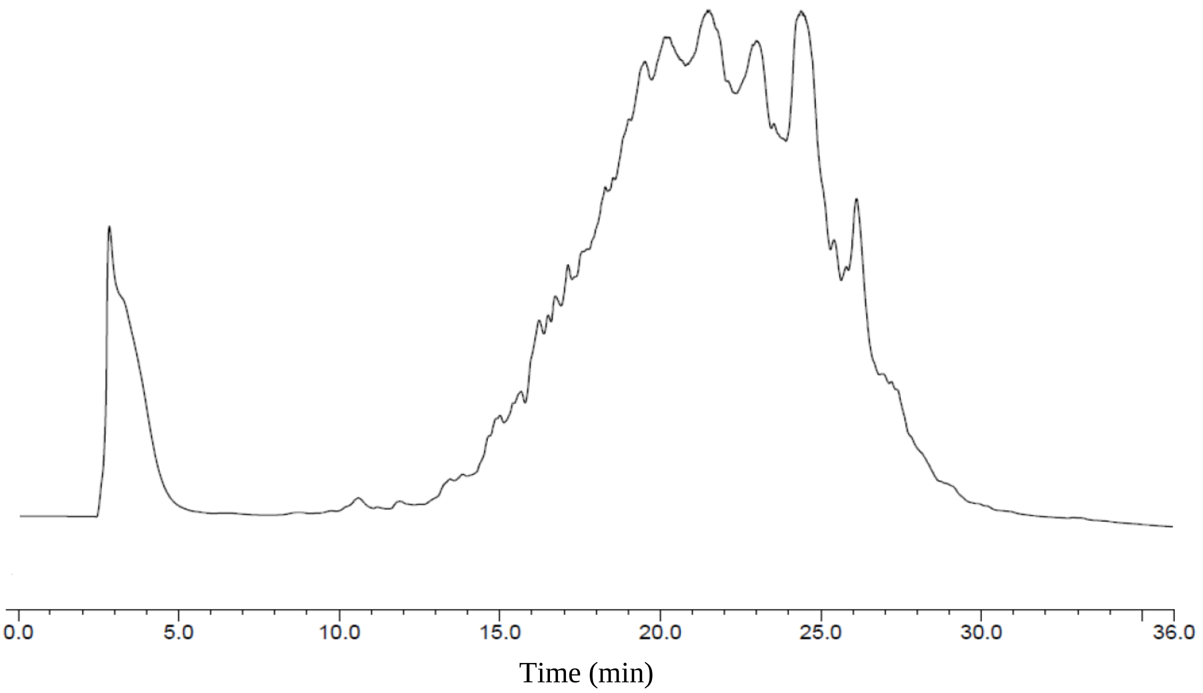

Figure S1. Analytical RP-HPLC (214 nm) chromatogram of crude peptide, **12**. Chromatographic separation was performed on Agilent 300SB-C_3 column (5 μm; 3.0 × 150 mm) and a linear gradient of 5-95% B over 30 min at room temperature, *ca*. 3% B per min at a flow rate of 0.3 mL/min. Buffer A: H_2O containing 0.1% TFA (*v/v*); buffer B: acetonitrile containing 0.1 % TFA (*v/v*).

S.3 Second synthetic approach towards the synthesis of cathelicidin-PY using automation under room temperature using AM-PS resin

Method 1: Loading Aminomethyl Polystyrene (AMPS) resin with Fmoc-Gly HMPP linker

To AM-PS resin (79.4 mg, 0.1 mmol, 1.26 $mmolg^{-1}$) pre-swollen in CH_2Cl_2 (5 mL, 10 min), was added a solution of Fmoc-Gly-HMPP linker (95 mg, 0.2 mmol, 2.0 equiv.) in CH_2Cl_2 (2 mL) followed by addition of DIC (31 μL, 0.2 mmol, 2.0 equiv.). The reaction was gently agitated at room temperature for 2 h. The resin was filtered and washed with CH_2Cl_2 (3 × 3 mL) and DMF (3 × 3 mL).

Method 2: General procedure for removal of N^{α}-Fmoc protecting group

The peptidyl resin was treated with a solution of 20% *v/v* piperidine in DMF (4 mL) and the mixture agitated at room temperature for 2 × 7 min, filtered and the resin washed with DMF.

Method 3: General procedure for amino acid coupling using HCTU

Fmoc-AA-OH (0.5 mmol, 5.0 equiv.) with HCTU (206.86 mg, 0.45 mmol, 4.5 equiv.) and NMM (109.94 μl, 1.0 mmol, 10 equiv.) in DMF (4 ml) was activated for 2 mins. This mixture was then added to the peptidyl resin (0.1 mmol), agitated in room temperature for 1 h. The resin was filtered and washed with DMF.

Method 4: General procedure for capping the free amino groups:

Fmoc-protected peptidyl resin was treated with a solution of acetic anhydride and NMM in DMF (1 mL, 20:20:60 *v/v/v*) after every coupling and the mixture was agitated at room temperature for 1 min. The resin was filtered and washed with DMF.

Method 5: TFA mediated resin cleavage and global deprotection

Peptidyl resin was treated with a mixture of TFA/H_2O/TIS (90:5:5, *v/v/v*, 10 mL) for 180 min. The filtrate was concentrated under a gentle stream of N_2 followed by the addition of cold diethyl ether to form a precipitate. The mixture was centrifuged, and the solution was carefully decanted off and discarded. The solid pellet was dissolved H_2O (20 mL) and lyophilised.

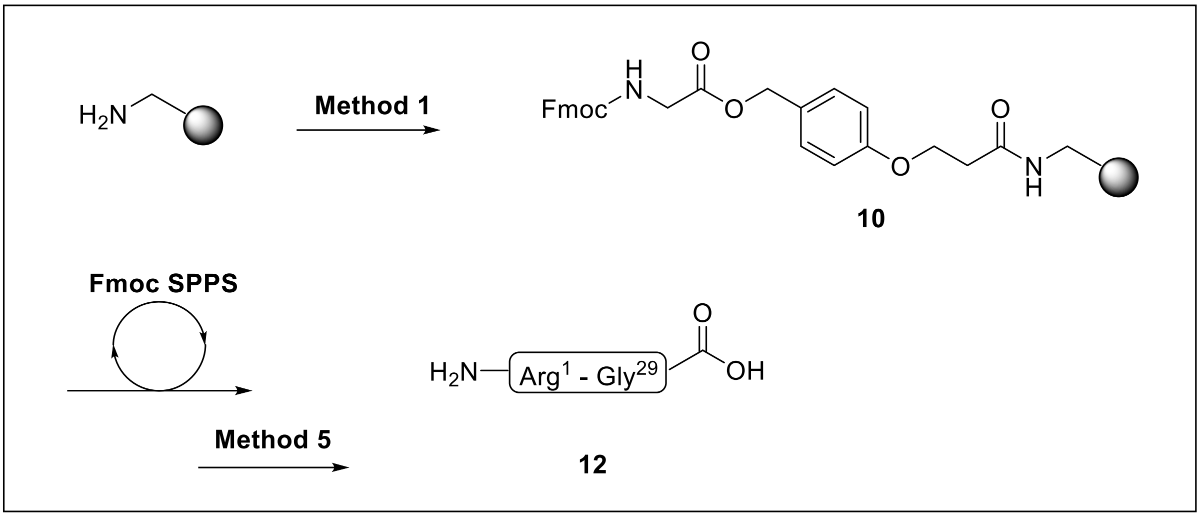

Scheme S2. Synthesis of native linear cathelicidin-PY peptide on AM-PS resin.

Fmoc-SPPS was used for the synthesis of cathelicidin-PY employing aminomethyl polystyrene (AM-PS) resin, functionalizing with Fmoc-Gly-HMPP linker using **Method 1**. The N^{α}-Fmoc protecting group was removed using **Method 2**. To resin bound NH_2-Gly-HMPP linker the appropriate amino acids were coupled using **Method 3**. Unreacted amino groups were capped using **Method 4**, followed by Fmoc-removal using **Method 2**. Resulting peptide was cleaved using **Method 5** to afford **12** as a white solid (161.23 mg, 47.1% yield, 35% crude purity).

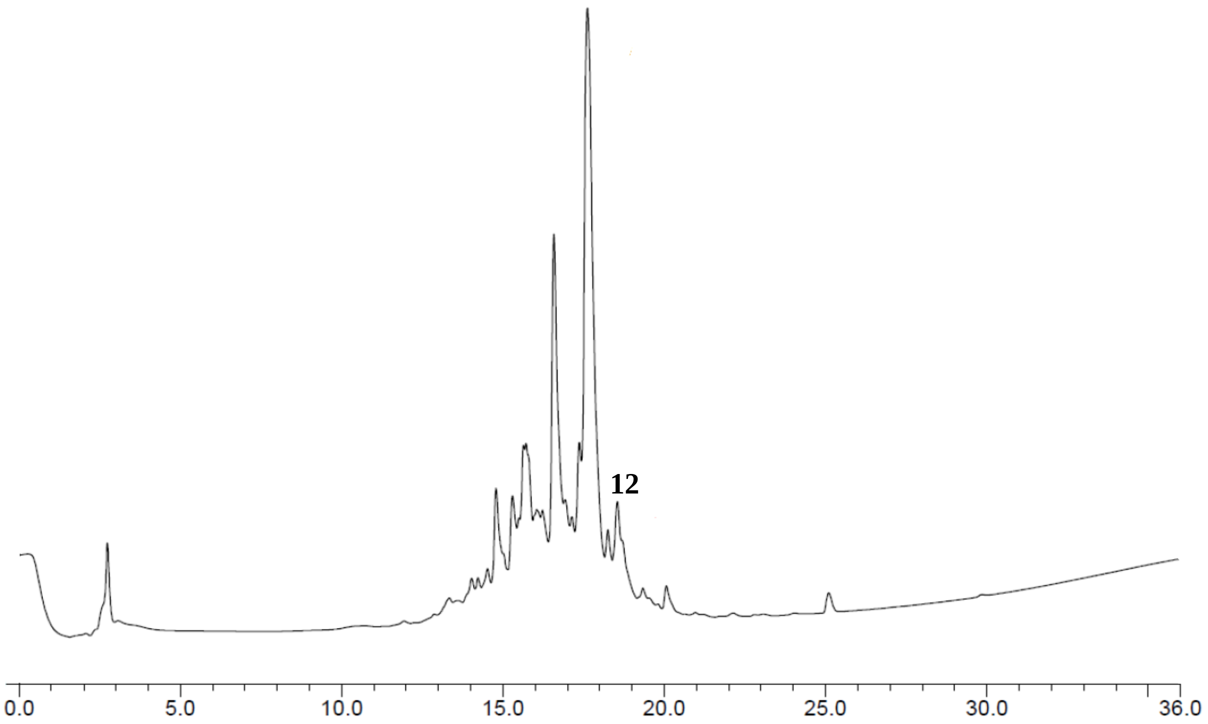

Figure S2. Analytical RP-HPLC chromatogram (214 nm) of crude linear cathelicidin-PY, **12** (t_R 18.5 min). Chromatographic separation was performed on, Phenomenex Jupiter C18 column, (5 µm; 3.0 × 150 mm), linear gradient of 5-95% over 30 min at room temperature, *ca*. 3% B min^{-1} at a flow rate of 0.5 mL/min. Buffer A: H_2O containing 0.1% TFA (*v/v*); buffer B: acetonitrile containing 0.1 % TFA (*v/v*).

S.4 Synthesis of cathelicidin-PY on TentaGel HL NH_2 resin using automated peptide synthesis in room temperature

Method 1: Loading TentaGel® HL NH2 with Fmoc-Gly HMPP linker

TentaGel HL NH_2 resin (208 mg, 0.1 mmol, 0.48 mmolg^{-1}) was loaded with Fmoc-Gly-HMPP linker (95 mg, 0.2 mmol, 2.0 equiv.) using DIC (31 μL, 0.2 mmol, 2.0 equiv.) in CH_2Cl_2 (2 mL) with a few drops of DMF for 2 hours in room temperature. The resin was filtered and washed with DMF (3 × 5 mL) and dried under vacuum to obtain TentaGel HL NH_2 loaded with Fmoc-Gly-HMPP linker, **13**

Method 2: General procedure for removal of N^{α}-Fmoc protecting group

The peptidyl resin was treated with a solution of 20% piperidine in DMF (4 mL, *v/v*) and the mixture agitated at room temperature for 2 × 7 min, filtered and the resin washed with DMF.

Method 3: General procedure for amino acid coupling using HCTU

Fmoc-AA-OH (0.50 mmol, 5.0 equiv.) with HCTU (186.2 mg, 0.45 mmol, 4.5 equiv.) and NMM (109.94 μl, 1.0 mmol, 10 equiv.) in DMF (4 mL) was activated for 2 mins. This mixture was then added to the peptidyl resin (0.1 mmol), agitated in room temperature for 1 h. The resin was filtered and washed with DMF.

Method 4: Double coupling of Fmoc-AA-OH from residues Cys^7 to Arg^1

Coupling was performed using the appropriate Fmoc-AA-OH (0.5 mmol, 5.0 equiv.), HATU (206.86 mg, 0.45 mmol, 4.5 equiv.) and NMM (101.1 μl, 0.1 mmol, 10 equiv.) in DMF for 1 h, r.t and repeated once more using fresh reagents. The resin was filtered and washed with DMF.

Method 5: General procedure for capping the free amino groups:

The N^{α}-Fmoc protected peptidyl resin was treated with a solution of acetic anhydride and NMM in DMF (1 mL, 20:20:60 *v/v/v*) and the mixture was agitated at room temperature for 1 mins. The resin was filtered and washed with DMF.

Method 6: TFA mediated resin cleavage and global deprotection

The peptidyl resin was treated with a mixture of TFA/ H_2O/DODT/TIS (95:2:2:1, 10 mL, *v/v/v/v*) for 2 h at room temperature. The filtrate was concentrated under a gentle stream of N_2

followed by the addition of cold diethyl ether to form a precipitate. The mixture was centrifuged, and the solution was carefully decanted off and discarded. The solid pellet was dissolved H_2O (20 mL) and lyophilised.

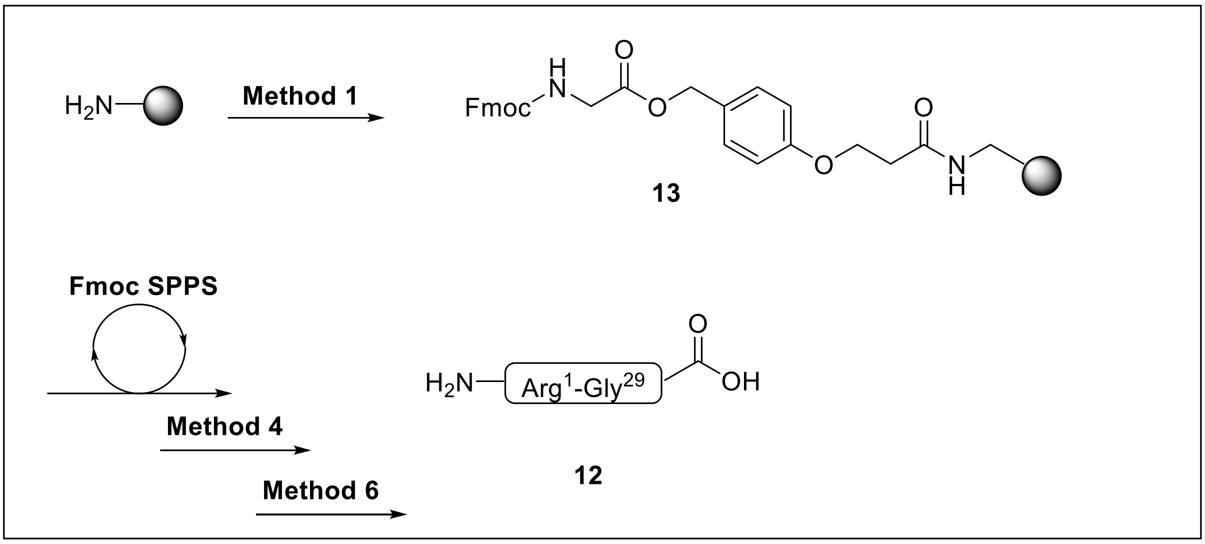

Scheme S3. Synthesis of linear cathelicidin-PY peptide, **12** on TentaGel HL NH_2 resin.

Fmoc-SPPS was used for the synthesis of linear cathelicidin-PY with TentaGel HL NH_2 resin, functionalizing with Fmoc-Gly-HMPP linker using **Method 1**. The N^{α}-Fmoc protecting group was removed using **Method 2**. To resin bound NH_2-Gly-HMPP linker the appropriate amino acids were coupled using **Method 3** up to Lys^8. The remainder of the last seven residues were double coupled following **Method 4**. Unreacted amino groups were capped using **Method 5**, followed by Fmoc-removal using **Method 2**. The resulting peptide was cleaved using **Method 6** to afford **12** as white solid (150 mg, 43.9% crude purity).

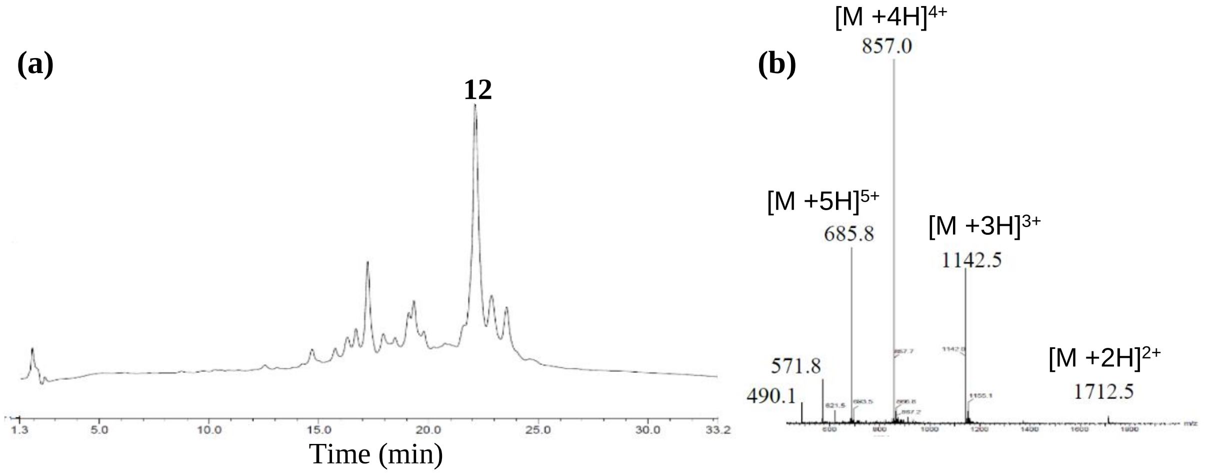

Figure S3. (a) Analytical RP-HPLC chromatograph (214 nm) of crude cathelicidin-PY, **12**; t_R 22.13 min. Chromatographic separation was performed on Thermo Scientific Dionex Ultimate 3000 HPLC using a XTerra® MS C-18 column (5 µm; 4.6 × 150 mm) on a linear gradient of 5-95% B in 35 min at room temperature, *ca*. 3% B per min at a flow rate of 1 mL/min. Buffer A: H_2O containing 0.1% TFA (*v/v*); buffer B: acetonitrile containing 0.1 % TFA (*v/v*). **(b)** ESI-MS, *m/z* for linear cathelicidin-PY, **12**, [$C_{151}H_{263}N_{47}O_{39}S_2$]: $[M+2H]^{2+}$ calculated: 1712.50, observed:1712.5, $[M+3H]^{3+}$ calculated: 1141.98 observed 1142.5, $[M+4H]^{4+}$ calculated: 856.74 observed 857.0, $[M+5H]^{5+}$ calculated: 685.59 observed 685.8; Mass calculated at 3422.95 Da.

S.5 Synthesis of linear cathelicidin-PY on lower substitution resin, TentaGel S NH_2 using automated peptide synthesiser

Method 1: Loading TentaGel® S NH_2 with HMPA linker

TentaGel S NH_2 resin (385 mg, 0.1 mmol, 0.26 mmolg^{-1}, resin particle size 90 μm) was pre-swollen in CH_2Cl_2 (5 mL) for 30 min in room temperature and CH_2Cl_2 drained. 4-(Hydroxymethyl)phenoxyacetic acid (HMPA linker) (72.9 mg, 0.4 mmol, 4 equiv.) and 6-Cl-HOBt (67.8 mg, 0.4 mmol, 4 equiv.) dissolved in DMF (2.0 mL) followed by addition of DIC (62.6 μl, 0.4 mmol, 4 equiv.) and added to the swollen resin. The reaction mixture was agitated at room temperature for 3 hours. The resin filtered and washed with DMF (3 × 3 mL) after which a negative ninhydrin test confirmed successful coupling.

Method 2: Resin functionalisation for *C*-terminal acid peptides

The symmetrical anhydride of the N^{α}-Fmoc protected *C*-terminal amino acid was prepared with Fmoc-Gly-OH (297.31 mg, 1.0 mmol, 10 equiv.) by reacting with DIC (78.3 μl, 0.5mmol, 5 equiv.) in CH_2Cl_2 for 10 mins at room temperature. The resulting symmetric anhydride (0.5 mmol, 5 equiv.) was manually esterified with the resin bound HMPA linker via acyl transfer, using a catalytic amount of DMAP (1.22 mg, 0.01 mmol, 0.1 equiv.), in DMF for 2 h at room temperature, before the resin was filtered and washed with DMF.

Method 3: General procedure for removal of N^{α}-Fmoc protecting group

The peptidyl resin was treated with a solution of 20% piperidine in DMF (4 mL, *v/v*) and the mixture agitated at room temperature for 2 × 7 min, filtered and the resin washed with DMF.

Method 4: General procedure for amino acid coupling using HATU

Fmoc-AA-OH (0.50 mmol, 5.0 equiv.) with HATU (171.1 mg, 0.45 mmol, 4.5 equiv.) and NMM (1.0 mmol, 10 equiv.) in DMF (4 mL) was activated for 2 mins. This mixture was then added to the peptidyl resin (0.1 mmol), agitated in room temperature for 1 h. The resin was filtered and washed with DMF (4 mL) for 4 × 0.5 mins.

Method 5: Manual coupling procedures for Fmoc-Cys(Trt)-OH

Manual coupling of Fmoc-Cys(Trt)-OH (292.85 mg, 0.5 mmol, 5 equiv.) with PyAOP (260.7 mg, 0.5 mmol, 5 equiv.) and *sym*-collidine (65.08 µl, 0.5 mmol, 5 equiv.) in DMF (7 mL) was performed for 1 hour at room temperature. The resin was filtered and washed with DMF.

Method 6: General procedure for capping the free amino groups:

The N^{α}-Fmoc-protected peptidyl resin was treated with a solution of acetic anhydride and NMM in DMF (1 mL, 20:20:60 *v/v/v*) and the mixture was agitated at room temperature for 1 mins. The resin was filtered and washed with DMF (4 mL) for 4 × 0.5 mins.

Method 7: TFA mediated resin cleavage and global deprotection

Peptidyl resin was treated with a mixture of TFA/H_2O/DODT/TIS (95:2:2:1, 10 mL, *v/v/v/v*) for 120 min at room temperature. The filtrate was concentrated under a gentle stream of N_2 followed by the addition of cold diethyl ether to form a precipitate. The mixture was centrifuged, and the solution was carefully decanted off and discarded. The solid pellet was dissolved H_2O (20 mL) and lyophilised.

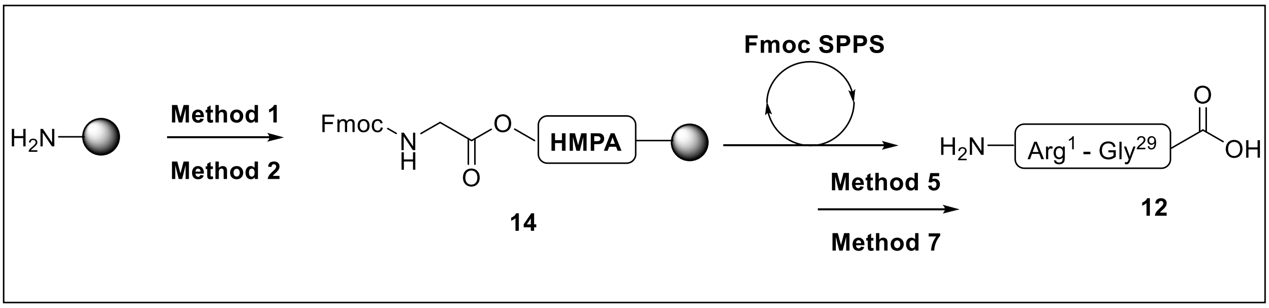

Scheme S4. Synthesis of linear cathelicidin-PY peptide, **12** on TentaGel S NH_2 resin**.**

Fmoc-SPPS was used for the synthesis of cathelicidin-PY employing TentaGel S NH_2 resin, functionalizing with HMPA linker using **Method 1**. Followed by coupling the symmetrical anhydride of the N^{α}-Fmoc protected *C*-terminal amino acid using **Method 2**. The N^{α}-Fmoc protecting group was removed using **Method 3**. To resin bound NH_2-Gly-HMPA linker the appropriate amino acids were coupled using **Method 4**. Cysteine residues Cys^3 and Cys^7 were manually coupled according to **Method 5**. Unreacted amino groups were capped using **Method 6**, followed by Fmoc-removal using **Method 3**. Resulting peptide was cleaved using **Method 7** to afford **12** as a white solid (200.5 mg, 49% crude purity).

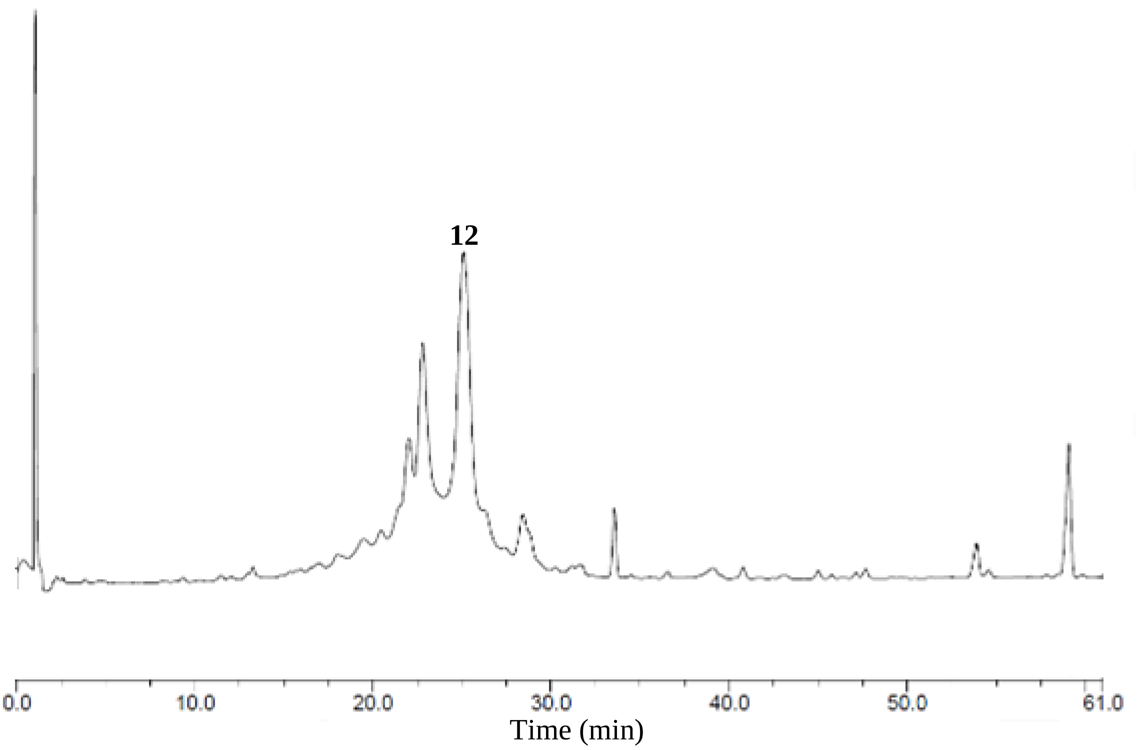

Figure S4. Analytical RP-HPLC chromatograph (214 nm) of crude linear cathelicidin-PY, **12**; t_R 26.4 min. Chromatographic separation was performed on a Thermo Scientific Dionex Ultimate 3000 HPLC using a XTerra® MS C-18 column (5 µm; 4.6 × 150 mm) and a linear gradient of 5 – 65% B in 60 min at room temperature, *ca.* 1% B per min at a flow rate of 1.0 mL/min. Buffer A: H_2O containing 0.1% TFA (*v/v*); Buffer B: acetonitrile containing 0.1 % TFA (*v/v*).

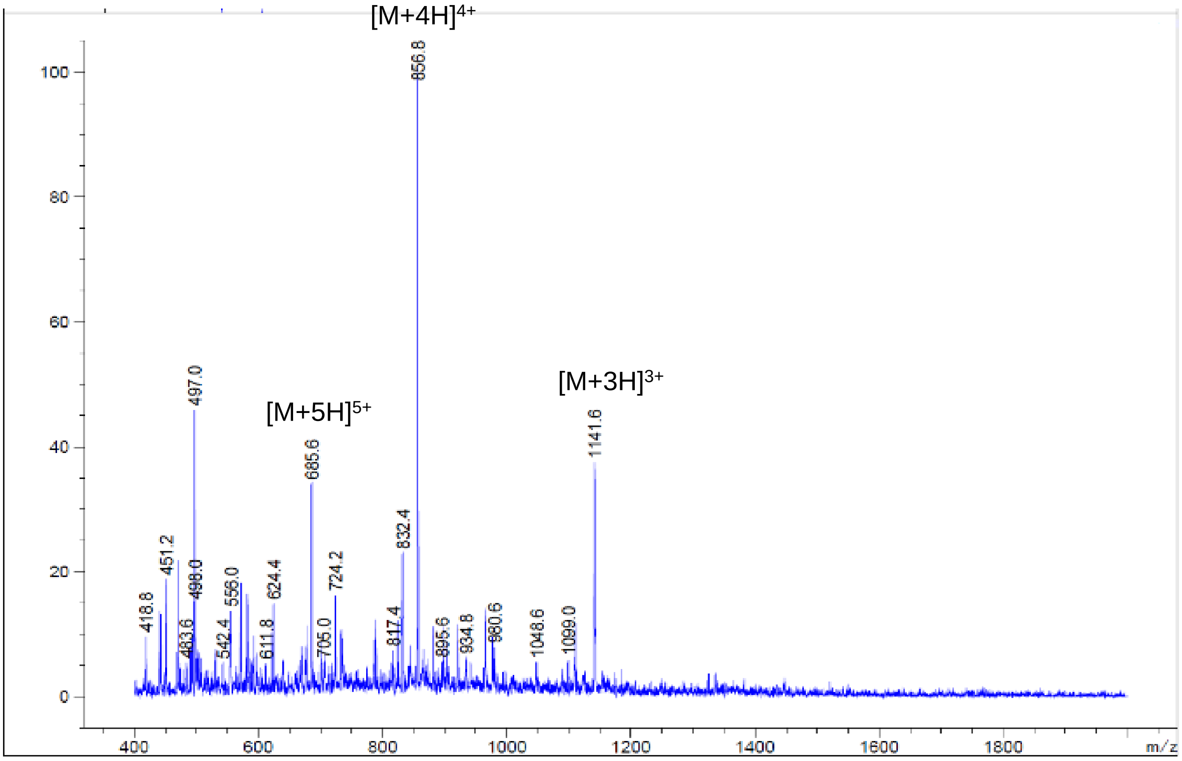

Figure S5. ESI-MS *m/z* for crude linear cathelicidin-PY, **12**; [$C_{151}H_{263}N_{47}O_{39}S_2$]: $[M+3H]^{3+}$ calculated: 1141.98 observed 1141.6, $[M+4H]^{4+}$ calculated: 856.74 observed 856.8, $[M+5H]^{5+}$ calculated: 685.59 observed 685.6; Mass calculated at 3422.95 Da.

S.6 Synthesis of linear cathelicidin-PY on ChemMatrix resin using flow chemistry

Method 1: Loading ChemMatrix® resin with HMPA linker

ChemMatrix resin (300 mg, 0.15 mmol, loading 0.6 mmolg^{-1}) of the larger particle size (130 μm) was pre-swollen in CH_2Cl_2 (25 mL, 30 min). HMPA linker (145.76 mg, 0.4 mmol, 4 equiv.) and *6*-Cl-HOBt (135.66 mg, 0.4 mmol, 4 equiv.) was dissolved in DMF (5.0 mL), mixture was added to pre-swollen resin followed by addition of DIC (125.26 μl, 0.4 mmol, 4 equiv.). The reaction mixture was gently agitated at room temperature for 3 hours. The resin was filtered and washed with DMF (3 × 3 mL) after which a negative ninhydrin test confirmed successful coupling.

Method 2: Resin functionalisation for *C*-terminal acid peptides

The symmetrical anhydride of the N^{α}-Fmoc protected *C*-terminal amino acid was prepared with Fmoc-Gly-OH (446 mg, 1.5 mmol, 10 equiv.) by reacting with DIC (77 μl, 0.5 mmol, 5 equiv.) in CH_2Cl_2 for 10 mins at room temperature. The resulting symmetric anhydride (0.5 mmol, 5 equiv.) was manually esterified with the resin bound HMPA linker via acyl transfer, using a catalytic amount of DMAP (1.83 mg, 0.01 mmol, 0.1 equiv.), in DMF for 2 h at room temperature.

Method 3: General procedure for removal of N^{α}-Fmoc protecting group

The peptidyl resin was treated with a solution of 30% piperidine in DMF (40 s, 10 mL, *v/v*) at 70 ^{o}C and resin washed by DMF (40s, 10 mL) removing any residual capping solution.

Method 4: General procedure for amino acid coupling using HATU

The freshly prepared Fmoc-AA-OH (0.30 M, 20 equiv.) with coupling reagent HATU (1.11 g, 2.9 mol, 19.5 equiv.) was dissolved in DMF (10 mL). DIPEA (1.02 mL, 5.85 mmol, 39 equiv.) was added to pre-activate the coupling solution 20 seconds prior to delivery to the on-resin peptide bearing free N^{α}-amino group. The activated amino acid solution was delivered to the resin (~40 s, 10 mL) and the delivery line is washed by drawing a further DMF (20 s, 5 mL). The peptidyl resin was further washed with DMF, removing any residual amino coupling solution (40 s, 10 mL).

Method 5: Coupling procedures for Fmoc-Cys(Trt)-OH

Fmoc-Cys(Trt)-OH (1.76 g, 3.0 mmol, 20 equiv.), PyAOP (1.525 g, 2.9 mmol, 19.5 equiv.), *sym*-collidine (761.4 µl, 5.85 mmol, 39 equiv.) in DMF (10 mL). The activated amino acid solution was delivered to the resin (~40 s, 10 mL) and the delivery line is washed by drawing a further DMF (20 s, 4 mL). The peptidyl resin was further washed with DMF, removing any residual amino acid coupling solution (40 s, 10 mL).

Method 6: TFA mediated resin cleavage and global deprotection

The peptidyl resin was treated with a mixture of TFA/H_2O/DODT/TIS (95:2:2:1, 10 mL, *v/v/v/v*) for 2 h at room temperature. The filtrate was concentrated under a gentle stream of N_2 followed by the addition of cold diethyl ether to form a precipitate. The mixture was centrifuged, and the solution was carefully decanted off and discarded. The solid pellet was dissolved H_2O (20 mL) and lyophilised.

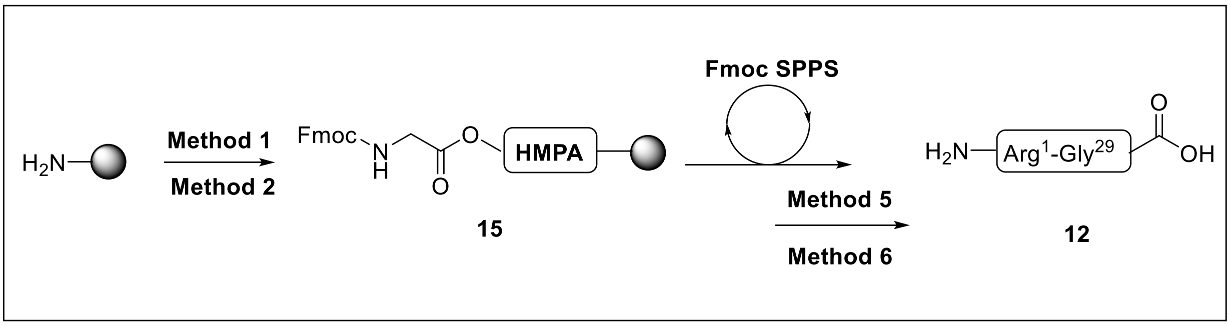

Scheme S5. Synthesis of linear cathelicidin-PY peptide, **12** on ChemMatrix® resin using flow chemistry.

Fmoc-SPPS was used for the synthesis of cathelicidin-PY with ChemMatrix resin, functionalizing with HMPA linker according to **Method 1**. Followed by symmetrical anhydride of the N^{α}-Fmoc protected *C*-terminal amino acid using **Method 2**. The Fmoc-Gly-HMPA linker loaded resin (0.15 mmol) was packed into the metal reactor for peptide synthesis under manual flow chemistry. The manual flow synthesis was initiated with pre-washing the resin with DMF (120 s, 30 mL) and performing the initial removal of the N^{α}-Fmoc protecting group using **Method 3**. The freshly made amino acids were appropriately coupled using **Method 4**, and modified coupling conditions were used for Cysteine residues Cys^3 and Cys^7 according to **Method 5**, followed by Fmoc-removal using **Method 3**. Resulting peptide was cleaved using **Method 6** to afford **12** as a white solid (325.7 mg, 38% crude purity).

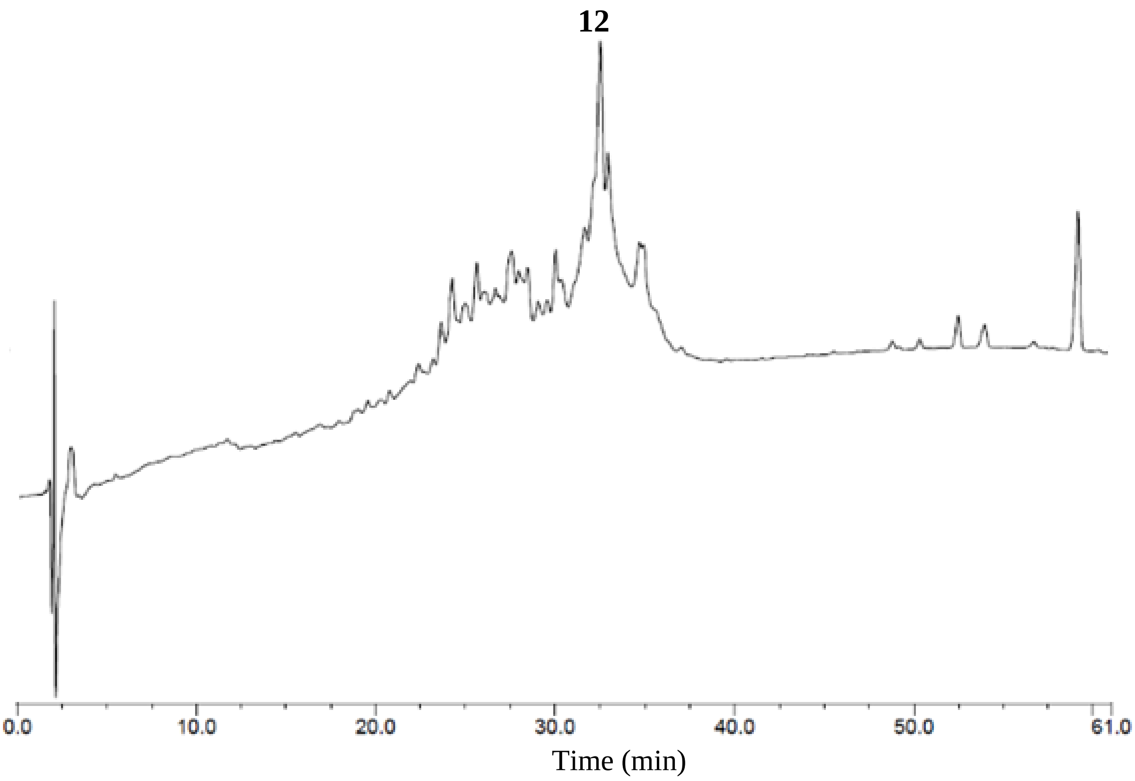

Figure S6. Analytical RP-HPLC chromatograph (214 nm) of crude linear cathelicidin-PY, **12**; t_R 33.10 min. Chromatographic separation was performed on a Thermo Scientific Dionex Ultimate 3000 HPLC using a XTerra® MS C-18 column (5 µm; 4.6 × 150 mm) and a linear gradient of 5 – 65% B in 60 min at room temperature, *ca.* 1% B per min at a flow rate of 1.0 mL/min. Buffer A: H_2O containing 0.1% TFA (*v/v*); Buffer B: acetonitrile containing 0.1 % TFA (*v/v*).

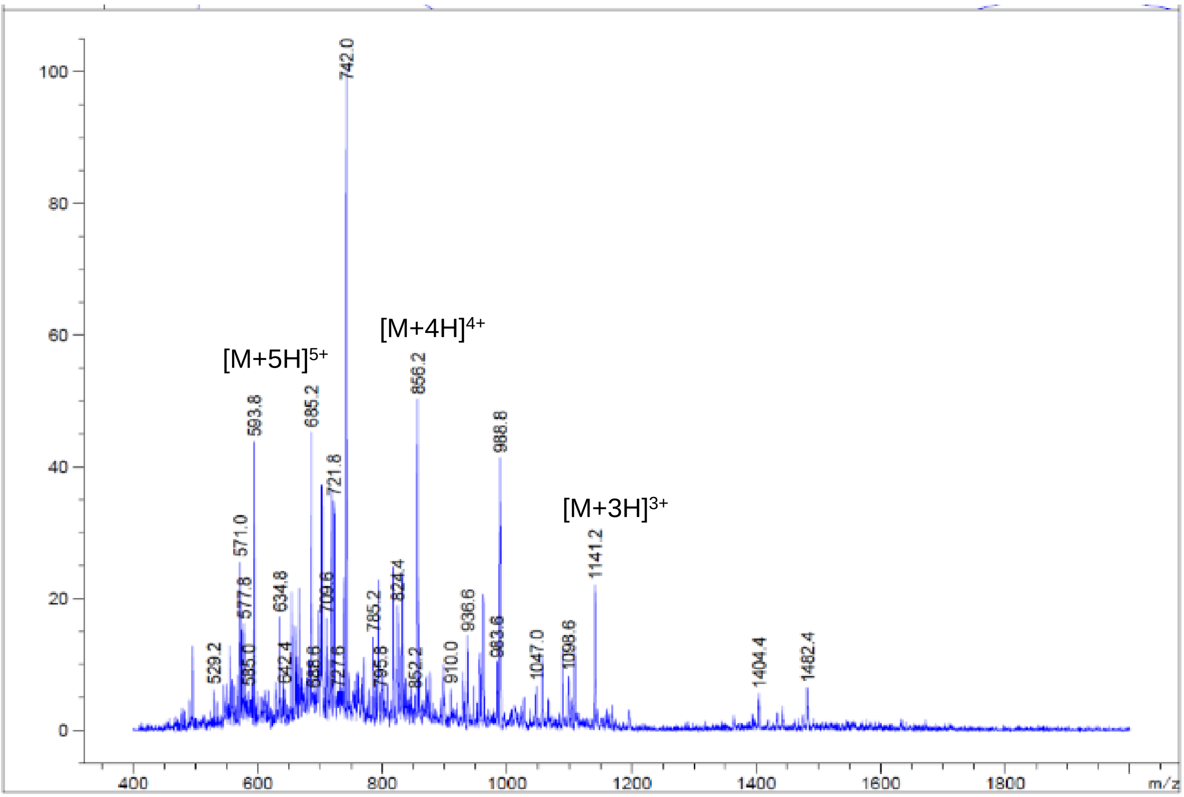

Figure S7. ESI-MS *m/z* for crude linear cathelicidin-PY, **12** [$C_{151}H_{263}N_{47}O_{39}S_2$] $[M+3H]^{3+}$ calculated: 1141.98 observed 1141.2, $[M+4H]^{4+}$ calculated: 856.74 observed 856.2, $[M+5H]^{5+}$ calculated: 685.59 observed 685.2; Mass calculated at 3422.95 Da.

S.7 Synthesis of linear cathelicidin-PY on TentaGel S NH_2 resin using flow chemistry

Method 1: Loading TentaGel® S NH_2 with HMPA linker

TentaGel® S NH_2 resin (600 mg, 0.15 mmol, 0.25 mmolg^{-1}) of particle size 130 μm was pre-swollen in CH_2Cl_2 (5 mL, 30 min). CH_2Cl_2 drained, 4-(hydroxymethyl)phenoxyacetic acid (HMPA linker) (136.64 mg, 0.75 mmol, 5 equiv.) and 6-Cl-HOBt (127.18 mg, 0.75 mmol, 5 equiv.) dissolved in DMF (2.0 mL) followed by addition of DIC (116.13 μl, 0.75 mmol, 5 equiv.) added to the swollen resin. The reaction mixture was gently agitated at room temperature for 3 hours. The resin was filtered and washed with DMF (3 × 3 mL) after which a negative ninhydrin test confirmed successful coupling.

Method 2: Resin functionalisation for *C*-terminal acid peptides

The symmetrical anhydride of the N$^{\alpha}$-Fmoc protected *C*-terminal amino acid was prepared with Fmoc-Gly-OH (445.97 mg, 1.5 mmol, 10 equiv.) by reacting with DIC (232.27 μl, 1.5 mmol, 10 equiv.) in CH_2Cl_2 for 10 mins at room temperature. The resulting symmetric anhydride (1.0 mmol, 10 equiv.) was manually esterified with the resin bound HMPA linker via acyl transfer, using a catalytic amount of DMAP (1.83 mg, 0.01 mmol, 0.1 equiv.) in DMF for 2 h at room temperature.

Method 3: General procedure for removal of N$^{\alpha}$-Fmoc protecting group

The peptidyl resin was treated with a solution of 30% piperidine and 5% formic acid in DMF (40 s, 10 mL, *v/v/v*) at 65 ^{o}C and resin washed by DMF (40s, 10 mL) removing any residual capping solution.

Method 4: General procedure for amino acid coupling using HATU

The freshly prepared Fmoc-AA-OH (0.30 M, 20 equiv.) with coupling reagent HATU (1.11 g, 2.9 mol, 19.5 equiv.) was dissolved in DMF (10 mL). DIPEA (1.019 mL, 5.85 mmol, 39 equiv.) was added to pre-activate the coupling solution for 20 seconds prior to delivery to the on-resin peptide bearing a free N$^{\alpha}$-amino group. The activated amino acid solution was fully delivered (~40 s, 10 mL) and the delivery line washed by drawing a further DMF (20 s, 4 mL). The peptidyl resin was further washed with DMF, removing any residual amino coupling solution (40 s, 10 mL).

Method 5: Coupling procedures for Fmoc-Cys(Trt)-OH and Fmoc-His(Trt)-OH

Freshly prepared Fmoc-AA-OH (0.30 M, 20 equiv.) in coupling reagent PyAOP (1.525 g, 2.9 mmol, 19.5 equiv.) was dissolved in DMF (10 mL). *Sym*-collidine (761 µl, 5.85 mmol, 39 equiv.) was added to pre-activate the coupling solution 20 seconds prior to delivery to the on-resin peptide bearing the free N^{α}-amino group. The activated amino acid solution is fully delivered (~40 s, 10 mL) and the delivery line is washed by drawing a further DMF (20 s, 4 mL). The peptidyl resin is further washed with DMF, removing any residual amino coupling solution (40 s, 10 mL).

Method 6: General procedure for capping the free amino groups:

The Fmoc-protected peptidyl resin was treated every 4th coupling cycle with a solution of 20% acetic anhydride in DMF (40 s, 10 mL, *v/v*) at 65 ^{o}C and resin washed thorough by DMF (80 s, 20 mL) removing any residual capping solution.

Method 7: On-resin iodine mediated disulfide bond formation between Cys(Trt) residues

The N^{α}-Fmoc protected peptidyl resin was treated with a fine powder of iodine (114.2 mg, 0.45 mmol, 3 equiv.) dissolved in DMF and agitated batchwise (2 × 5 min) at room temperature. Following iodine treatments, the resin was thoroughly washed with DMF (3 × 5 ml) and treated with 20% piperidine in DMF (2 x 5 ml, *v/v*) to remove the N^{α}-terminal Fmoc protecting group, followed by DMF wash (3 x 5 mL).

Method 8: TFA mediated resin cleavage and global deprotection

Peptidyl resin was treated with a mixture of TFA/H_2O/TIS (95:2.5:2.5 10 mL, *v/v/v*) for 120 min at room temperature. The filtrate was concentrated under a gentle stream of N_2 followed by the addition of cold diethyl ether to form a precipitate. The mixture was centrifuged, and the solution was carefully decanted off and discarded. The solid pellet was dissolved H_2O (20 mL) and lyophilised.

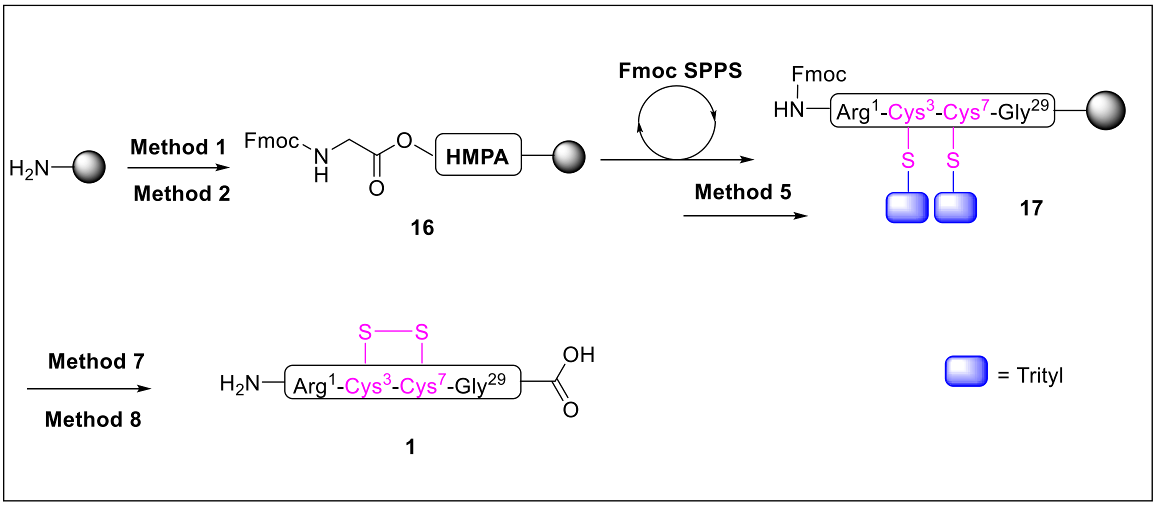

Scheme S6. Synthesis of native cathelicidin-PY peptide, **1** on TentaGel S NH_2 resin using flow chemistry.

Fmoc-SPPS was employed for the synthesis of linear cathelicidin-PY employing TentaGel S NH_2 resin, functionalizing with HMPA linker using **Method 1**. Followed by coupling the symmetrical anhydride of the N^{α}-Fmoc protected *C*-terminal amino acid using **Method 2**. The Fmoc-Gly-HMPA linker bound resin (0.15 mmol) was packed into the metal reactor for peptide synthesis under manual flow chemistry. The manual flow synthesis was initiated with pre-washing the resin with DMF (120 s, 30mL) and performing the initial removal of the N^{α}-Fmoc protecting group using **Method 3**. The freshly made amino acids were appropriately coupled using **Method 4**, modified coupling conditions were used for cysteine and histidine residues Cys^3, Cys^7 and His^{10} according to **Method 5**. Unreacted amino groups were capped every 4th coupling cycle using **Method 6**. At this stage a small portion of the resin beads was treated with **Method 3** to removal the N-terminal Fmoc protection and **Method 8** to afford a small quantity of crude linear peptide **12**, in 79% purity as judged by RP-HPLC peak area (214 nm). With the remainder of the peptidyl resin with the N^{α}-Fmoc protecting group still present, the peptidyl resin was treated with iodine according to **Method 7** to effect disulfide formation. The resulting peptide was cleaved using **Method 8** to afford **1** as a white solid (284.9 mg, 55% yield, crude purity 55.6 %). The crude peptide was purified by semi-preparative RP-HPLC using a Dionex UltiMate® 3000 on a Phenomenex Gemini C_{18} column (10 x 150 mm, 5 mm) using a linear gradient of 10 – 50% B over 40 min, (*ca.* 1.0% B min^{-1}) with a flow rate of 4.5 mL/min. Fractions were collected and analysed by ESI-

MS for compound identification. Fractions that were identified with the correct *m/z* were collected, combined and lyophilised to afford **1** as a white amorphous solid (148.15 mg, 52% yield, 95% purity).

ESI-HRMS: *m/z* calculated for $[C_{151}H_{261}N_{47}O_{39}S_2+4H]^{4+}$ calculated: 856.4924, observed 856.4910. **RP-HPLC:** $\mathbf{t_R}$ = 27.6 min.

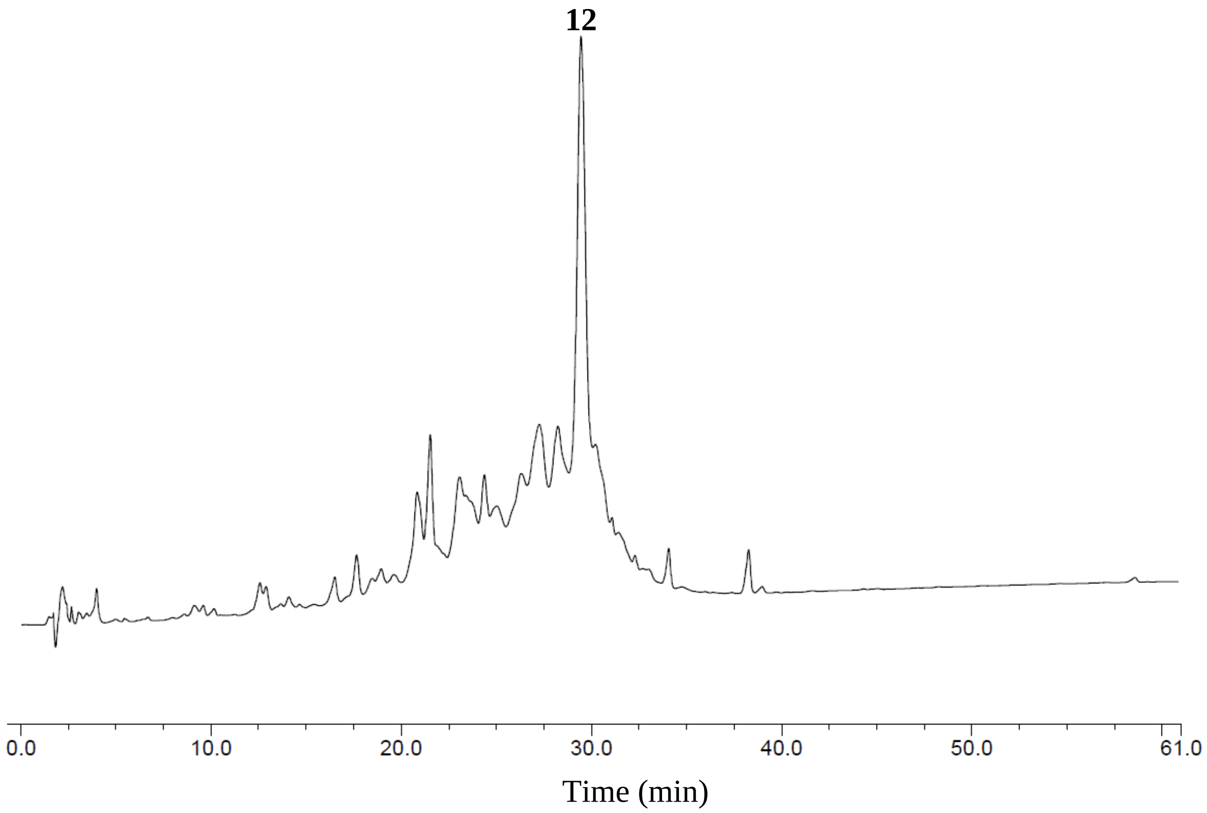

Figure S8. Analytical RP-HPLC chromatograph (214 nm) of mini cleaved crude linear cathelicidin-PY, **12**; t_R 29.4 min. Chromatographic separation was performed on a Thermo Scientific Dionex Ultimate 3000 HPLC using a XTerra® MS C-18 column (5 µm; 4.6 × 150 mm) and a linear gradient of 5 – 65% B in 60 min at room temperature, *ca.* 1% B per min at a flow rate of 1.0 mL/min. Buffer A: H_2O containing 0.1% TFA (*v/v*); Buffer B: acetonitrile containing 0.1 % TFA (*v/v*).

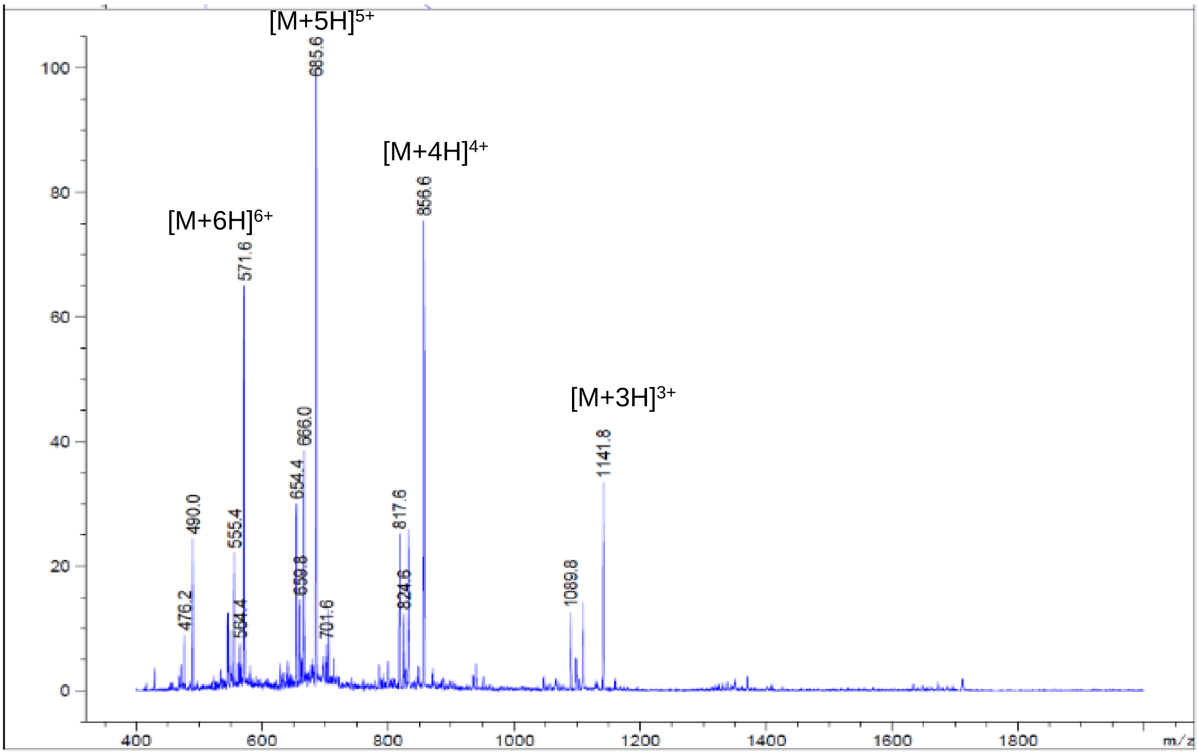

Figure S9. ESI-MS *m/z* for crude linear cathelicidin-PY, **12** [$C_{151}H_{263}N_{47}O_{39}S_2$]: $[M+3H]^{3+}$ calculated: 1141.98 observed 1141.8, $[M+4H]^{4+}$ calculated: 856.74 observed 856.6, $[M+5H]^{5+}$ calculated: 685.59 observed 685.6, $[M+6H]^{6+}$ calculated: 571.49 observed 571.6; Mass calculated at 3422.95 Da.

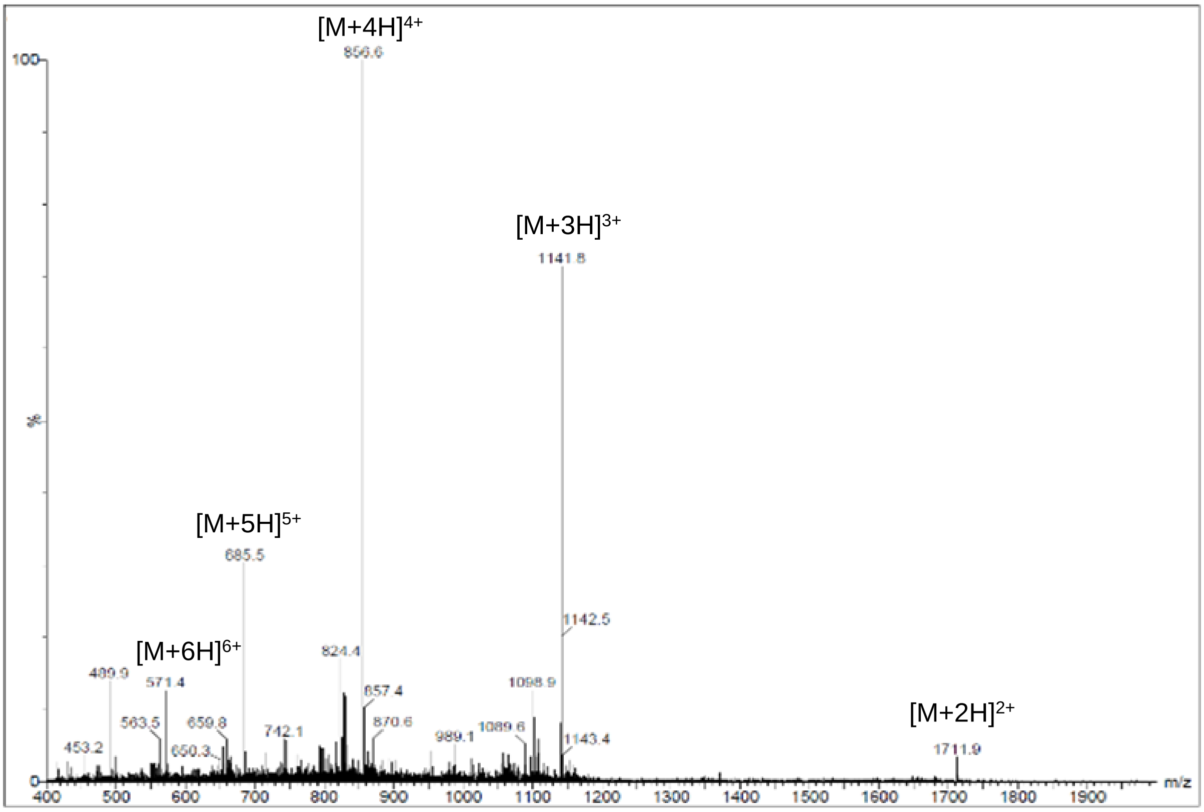

Figure S10. ESI-MS, *m/z* for crude linear cathelicidin-PY, **1**, [$C_{151}H_{261}N_{47}O_{39}S_2$]: $[M+2H]^{2+}$ calculated: 1711.46 observed 1711.9, $[M+3H]^{3+}$ calculated: 1141.31 observed 1141.8, $[M+4H]^{4+}$ calculated: 856.23 observed 856.6, $[M+5H]^{5+}$ calculated: 685.19 observed 685.5, $[M+6H]^{6+}$ calculated: 571.16 observed 571.4; Mass calculated at 3420.93 Da.

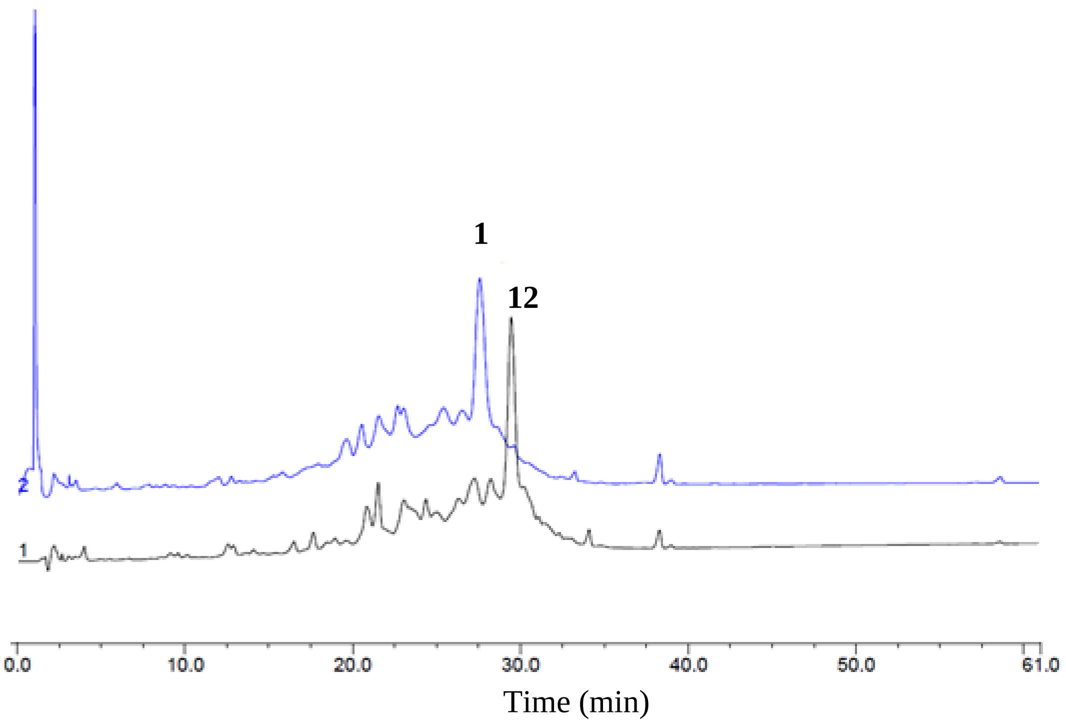

Figure S11. Analytical RP-HPLC chromatographic (214 nm) profile illustrating the cyclisation of cathelicidin-PY, **1;** t_R 27.6 min under iodide mediated disulfide formation with the linear precursor, **12**; t_R 29.4 min. Chromatographic separation was performed on a Thermo Scientific Dionex Ultimate 3000 HPLC using a XTerra® MS C-18 column (5 µm; 4.6 × 150 mm) and a linear gradient of 5 – 65% B in 60 min at room temperature, *ca.* 1% B per min at a flow rate of 1.0 mL/min. Buffer A: H_2O containing 0.1% TFA (*v/v*); Buffer B: acetonitrile containing 0.1 % TFA (*v/v*).

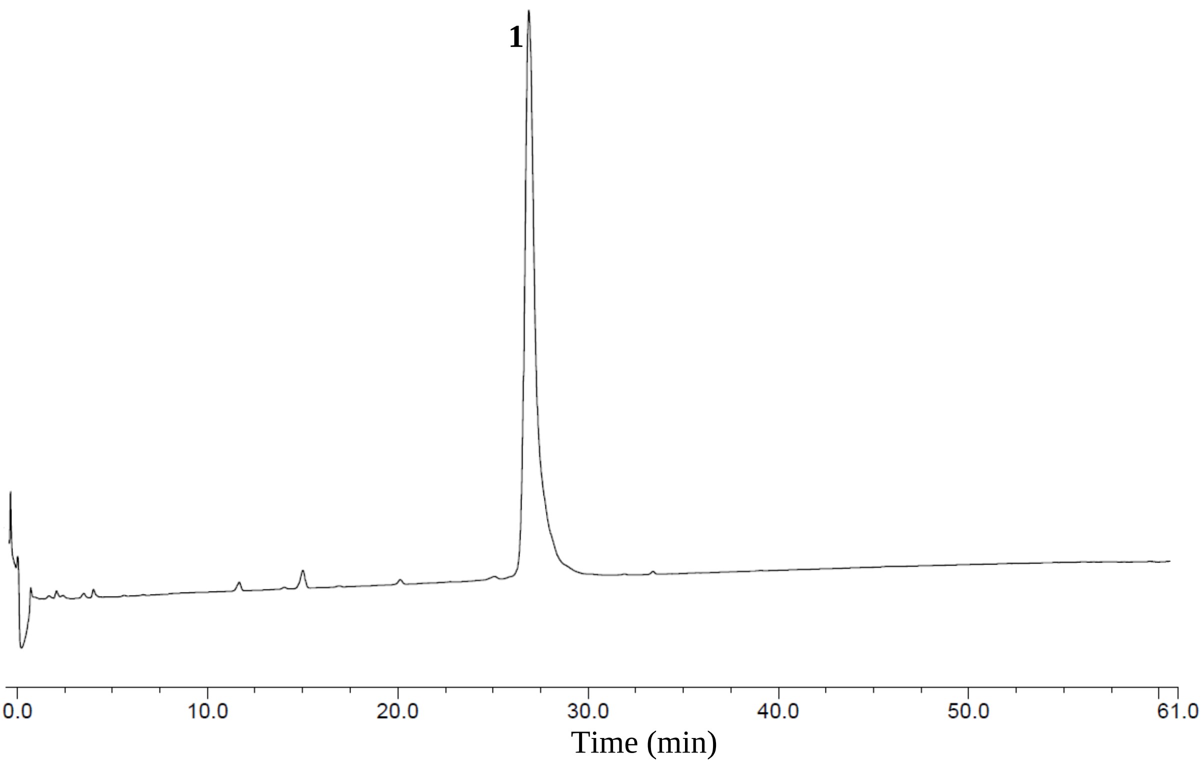

Figure S12. Analytical RP-HPLC chromatograph (214 nm) of purified cathelicidin-PY **1** t_R 29.9 min. Chromatographic separation was performed on a Thermo Scientific Dionex Ultimate 3000 HPLC using a XTerra® MS C-18 column (5 µm; 4.6 × 150 mm) and a linear gradient of 5 – 65% B in 60 min at room temperature, *ca.* 1% B per min at a flow rate of 1.0 mL/min. Buffer A: H_2O containing 0.1% TFA (*v/v*); Buffer B: acetonitrile containing 0.1 % TFA (*v/v*).

Acquisition Parameter

Source Type	ESI	Ion Polarity	Positive	Set Nebulizer	0.4 Bar
Focus	Active	Set Capillary	4500 V	Set Dry Heater	180 °C
Scan Begin	100 m/z	Set End Plate Offset	-500 V	Set Dry Gas	4.0 l/min
Scan End	4000 m/z	Set Collision Cell RF	650.0 Vpp	Set Divert Valve	Waste

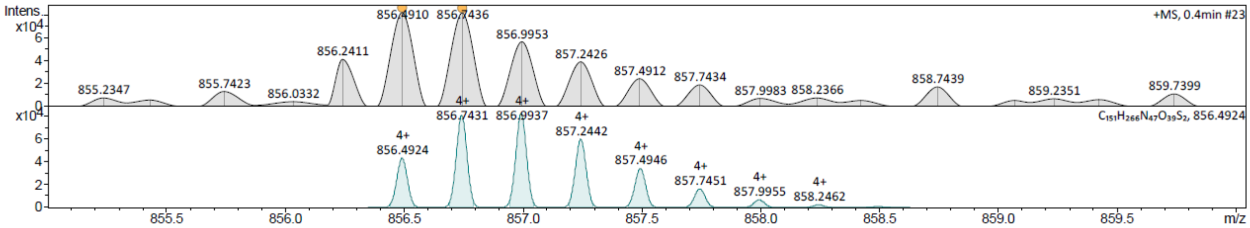

Meas. m/z	#	Ion Formula	m/z	err [ppm]	mSigma	# Sigma	Score	rdb	e⁻ Conf	N-Rule
856.4910	1	C151H266N47O39S2	856.4924	1.6	213.1	1	100.00	42.5	odd	ok
856.7436	1	C151H267N47O39S2	856.7443	0.8	309.3	1	100.00	42.0	even	ok

Figure S13. ESI-HRMS, formula analysis for $[C_{151}H_{261}N_{47}O_{39}S_2+4H]^{4+}$ calculated: 856.4924, observed 856.4910.

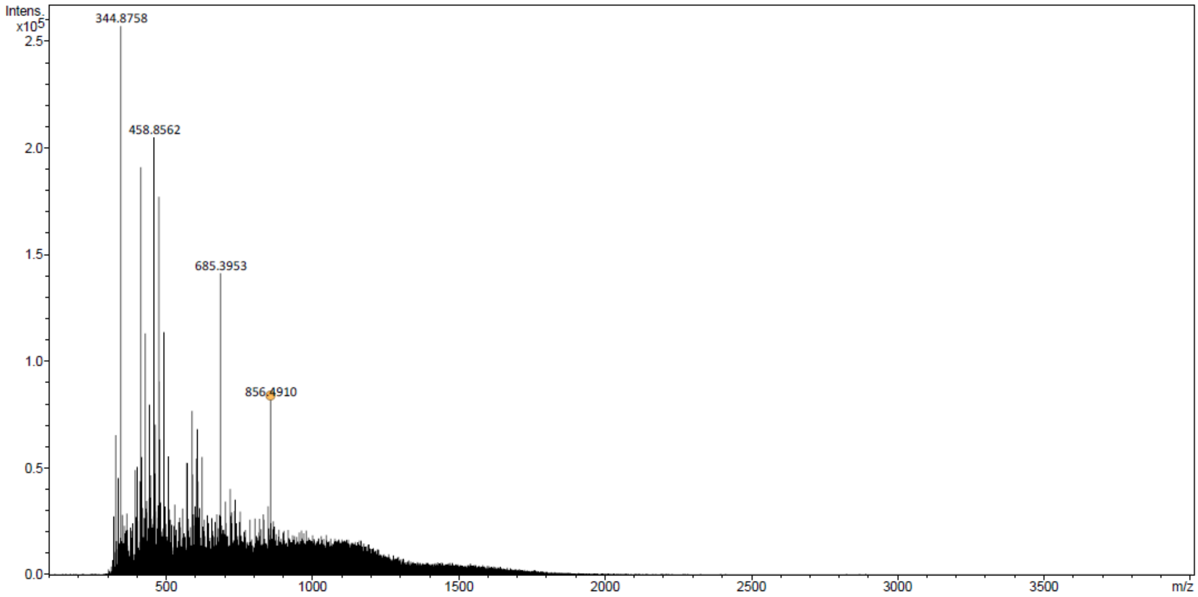

Figure S14. ESI-HRMS, m/z calculated for cathelicidin-PY, **1**, $[C_{151}H_{261}N_{47}O_{39}S_2+4H]^{4+}$ 856.4924, observed 856.4910.

S.8 Synthesis of linear Ala^3 and Ala^7 substituted cathelicidin-PY analogue (2) on TentaGel S NH_2 resin using flow chemistry

H_2N-RK**A**NFL**A**KLKEKLRTVITSHIDKVLRPQG-COOH **2**

Method 1: Loading TentaGel® S NH_2 with HMPA linker

TentaGel® S NH_2 resin (600 mg, 0.15 mmol, 0.25 mmolg^{-1}) with particle size 130 μm, was pre-swollen in CH_2Cl_2 (5 mL, 30 min). CH_2Cl_2 drained, 4-(hydroxymethyl)phenoxyacetic acid (HMPA linker) (136.64 mg, 0.75 mmol, 5 equiv.) and 6-Cl-HOBt (127.18 mg, 0.75 mmol, 5 equiv.) dissolved in DMF (2.0 mL) followed by addition of DIC (116.13 μl, 0.75 mmol, 5 equiv.) added to the swollen resin. The reaction mixture was gently agitated at room temperature for 3 hours. The resin was filtered and washed with DMF (3 × 3 mL) after which a negative ninhydrin test confirmed successful coupling.

Method 2: Resin functionalisation for *C*-terminal acid peptides

The symmetrical anhydride of the N$^{\alpha}$-Fmoc protected *C*-terminal amino acid was prepared with Fmoc-Gly-OH (445.97 mg, 1.5 mmol, 10 equiv.) by reacting with DIC (232.27 μl, 1.5 mmol, 10 equiv.) in CH_2Cl_2 for 10 mins at room temperature. The resulting symmetric anhydride (1.0 mmol, 10 equiv.) was manually esterified with the resin bound HMPA linker via acyl transfer, using a catalytic amount of DMAP (1.83 mg, 0.01 mmol, 0.1 equiv.) in DMF for 2 h at room temperature.

Method 3: General procedure for removal of N$^{\alpha}$-Fmoc protecting group

The peptidyl resin was treated with a solution of 30% piperidine, 5% formic acid in DMF (40 s, 10 mL, *v/v/v*) at 65 ^{o}C and resin washed by DMF (40s, 10 mL) removing any residual capping solution.

Method 4: General procedure for amino acid coupling

The freshly prepared Fmoc-AA-OH (0.30 M, 20 equiv.) with coupling reagent HATU (1.11 g, 2.9 mol, 19.5 equiv.) was dissolved in DMF (10 mL). DIPEA (1.019 mL, 5.85 mmol, 39 equiv.) was added to pre-activate the coupling solution for 20 seconds prior to delivery to the on-resin peptide bearing a free N$^{\alpha}$-amino group. The activated amino acid solution was fully delivered (~40 s, 10 mL) and the delivery line washed by drawing a further DMF (20 s, 4 mL).

The peptidyl resin was further washed with DMF, removing any residual amino coupling solution (40 s, 10 mL).

Method 5: General procedure for capping the free amino groups:

The Fmoc-protected peptidyl resin was treated every 4th coupling cycle with a solution of 20% acetic anhydride in DMF (40 s, 10 mL, *v/v*) at 65 ^{o}C and resin washed thorough by DMF (80 s, 20 mL) removing any residual capping solution.

Method 7: TFA mediated resin cleavage and global deprotection

Peptidyl resin was treated with a mixture of TFA/ H_2O/TIS (95:2.5:2.5, 10 mL, *v/v/v*) for 120 min at room temperature. The filtrate was concentrated under a gentle stream of N_2 followed by the addition of cold diethyl ether to form a precipitate. The mixture was centrifuged, and the solution was carefully decanted off and discarded. The solid pellet was dissolved H_2O (20 mL) and lyophilised.

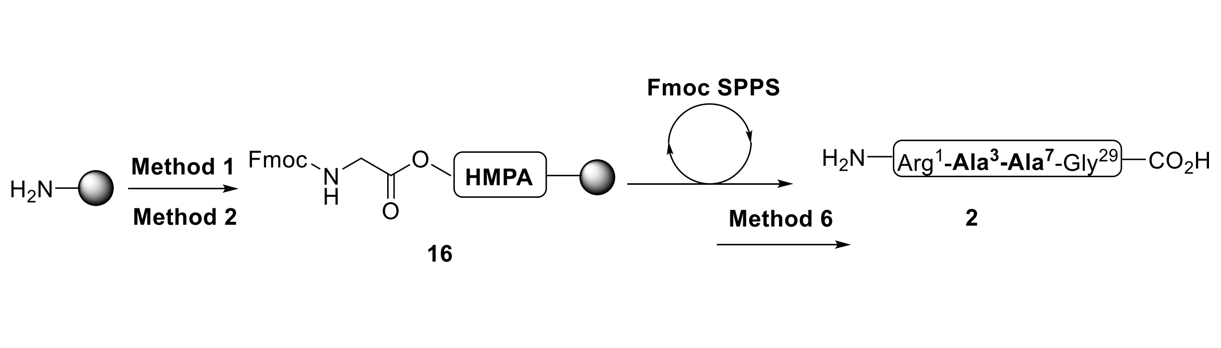

Scheme S7. Synthesis of Ala^{3} and Ala^{7} substituted linear cathelicidin-PY peptide, **2** using flow chemistry.

Fmoc-SPPS was used for synthesis of the linear peptide employing TentaGel S NH_2 resin, functionalized with HMPA linker using **Method 1**. Followed by coupling the symmetrical anhydride of the N^{α}-Fmoc protected *C*-terminal amino acid using **Method 2**. The Fmoc-Gly-HMPA linker bound resin (0.15 mmol) was packed into the metal reactor for peptide synthesis under manual flow chemistry. The manual flow synthesis was initiated by pre-washing the resin with DMF (120 s, 30mL) and performing the initial removal of the N^{α}-Fmoc protecting group using **Method 3**. The freshly made amino acids were appropriately coupled using **Method 4**. Unreacted amino groups were capped every 4th coupling cycle using **Method 5**, followed by Fmoc-removal using **Method 3**. The resulting peptide was cleaved using **Method 6** to afford **2** as a white solid (408.36 mg, 81% yield, crude purity of 51.6%). The crude peptide was purified by semi-preparative RP-HPLC using a Dionex UltiMate® 3000 on a Phenomenex Gemini C_{18} column (10 x 150 mm, 5 mm) using a linear gradient of 10 – 50% B over 40 min, (*ca.* 1.0% B min^{-1}) with a flow rate of 4.5 mL/min. Fractions were collected and analysed by ESI-MS for compound identification. Fractions that were identified with the correct *m/z* were collected, combined and lyophilised to afford, **2** as a white amorphous solid (212.35 mg, 52% yield, 98.2% purity).

ESI-HRMS: *m/z* calculated for $[C_{151}H_{263}N_{47}O_{39}+4H]^{4+}$ calculated:841.0103, observed 841.0127; **RP-HPLC:** t_R = 26.1 min.

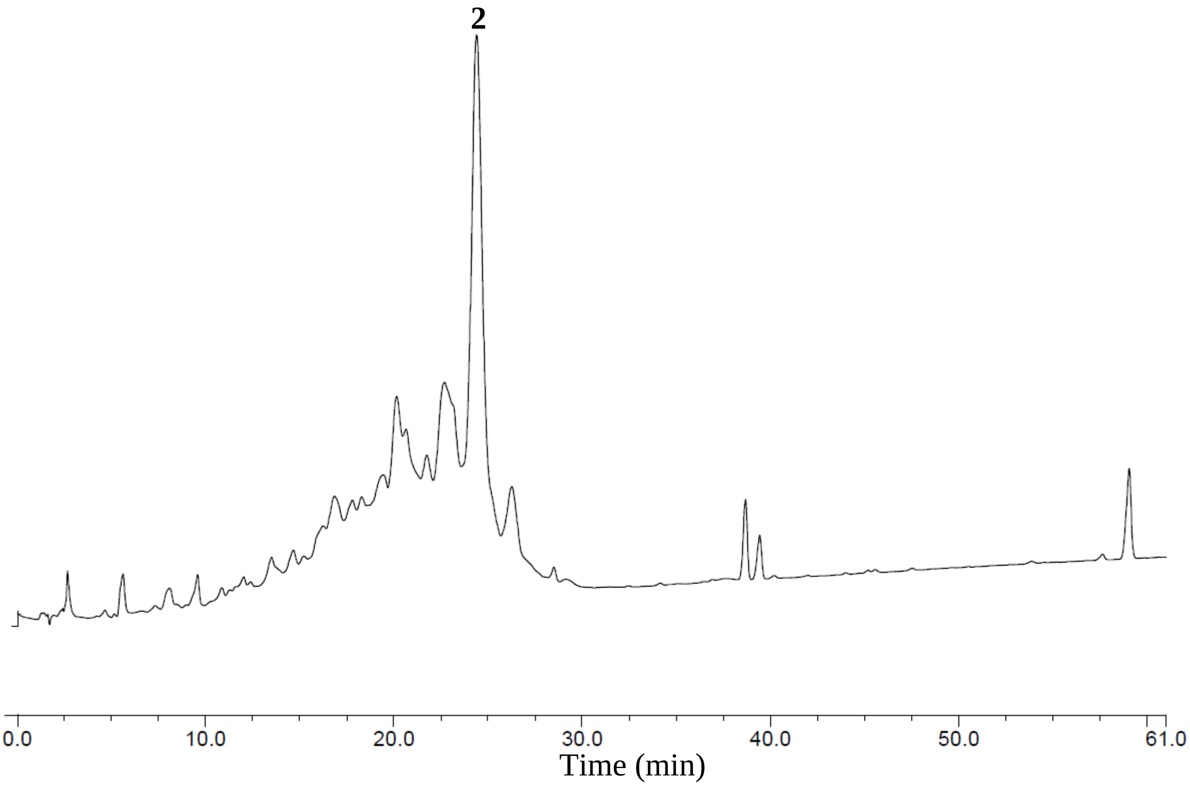

Figure S15. Analytical RP-HPLC chromatograph (214 nm) of crude cathelicidin-PY analogue, **2**; t_R 24.4 min. Chromatographic separation was performed on a Thermo Scientific Dionex Ultimate 3000 HPLC using a XTerra® MS C-18 column (5 µm; 4.6 × 150 mm) and a linear gradient of 5 – 65% B in 60 min at room temperature, *ca.* 1% B per min at a flow rate of 1.0 mL/min. Buffer A: H_2O containing 0.1% TFA (*v/v*); Buffer B: acetonitrile containing 0.1 % TFA (*v/v*).

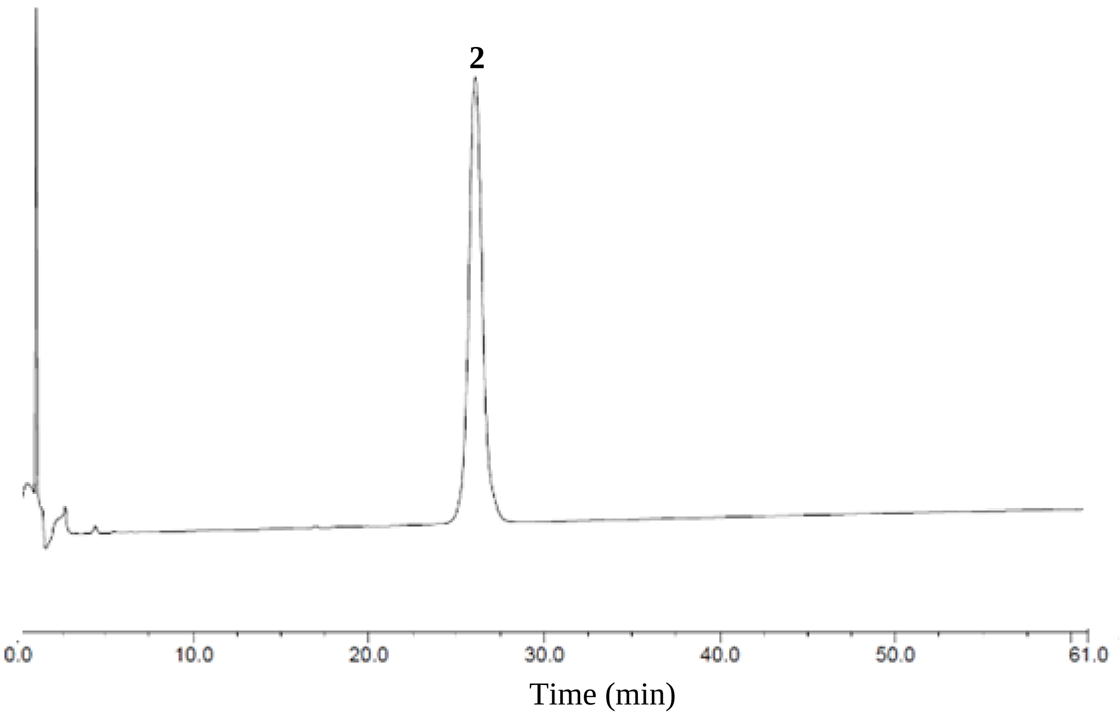

Figure S16. Analytical RP-HPLC chromatograph (214 nm) of purified cathelicidin-PY analogue, **2**; t_R 26.1 min. Chromatographic separation was performed on a Thermo Scientific Dionex Ultimate 3000 HPLC using a XTerra® MS C-18 column (5 µm; 4.6 × 150 mm) and a linear gradient of 5 – 65% B in 60 min at room temperature, *ca.* 1% B per min at a flow rate of 1.0 mL/min. Buffer A: H_2O containing 0.1% TFA (*v/v*); Buffer B: acetonitrile containing 0.1 % TFA (*v/v*).

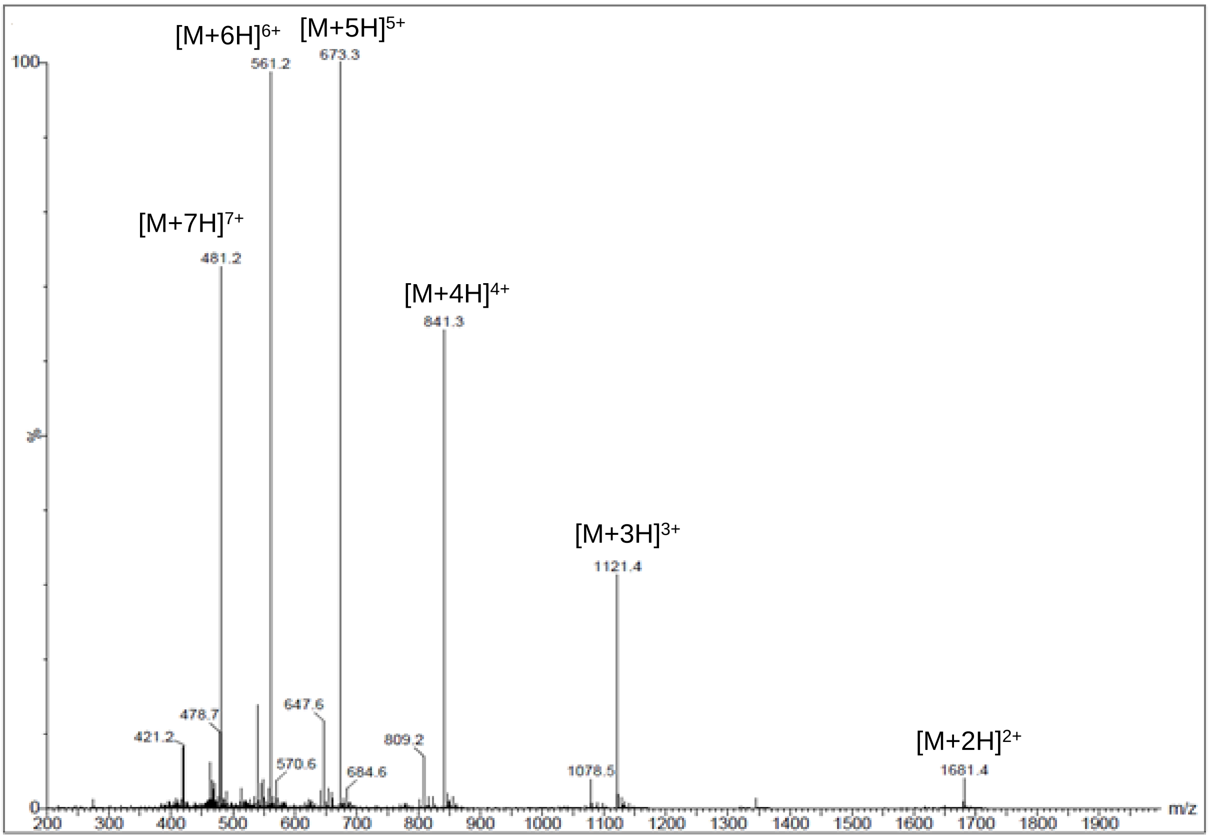

Figure S17. ESI-MS, *m/z* for purified linear analogue, **2**; [$C_{151}H_{263}N_{47}O_{39}$] $[M+2H]^{2+}$ calculated:1681.51, observed 1681.4; $[M+3H]^{3+}$ calculated: 1121.34, observed 1121.4; $[M+4H]^{4+}$ calculated: 841.25, observed 841.3; $[M+5H]^{5+}$ calculated: 673.20, observed 673.3; $[M+6H]^{6+}$ calculated: 561.17, observed 561.2; $[M+7H]^{7+}$ calculated: 481.14, observed 481.2; Mass calculated at 3361.01 Da.

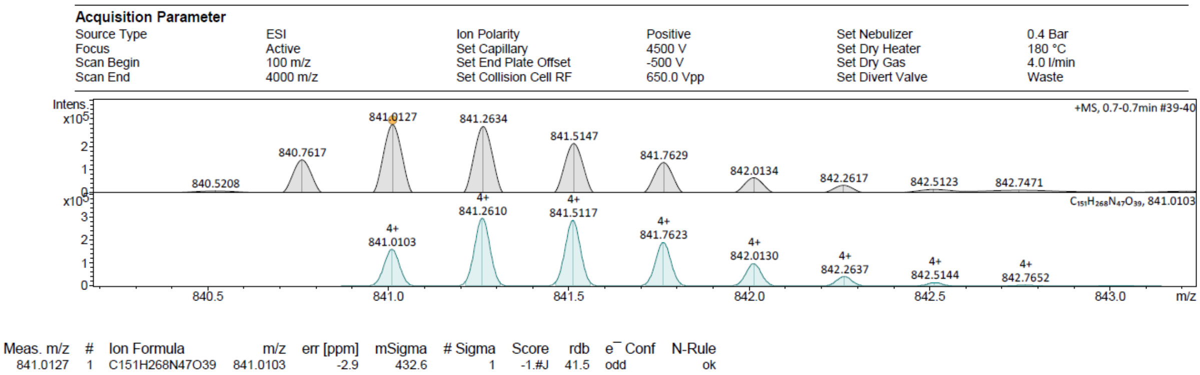

Figure S18. ESI-HRMS, formula analysis for $[C_{151}H_{263}N_{47}O_{39}+4H]^{4+}$ calculated:841.0103, observed 841.0127.

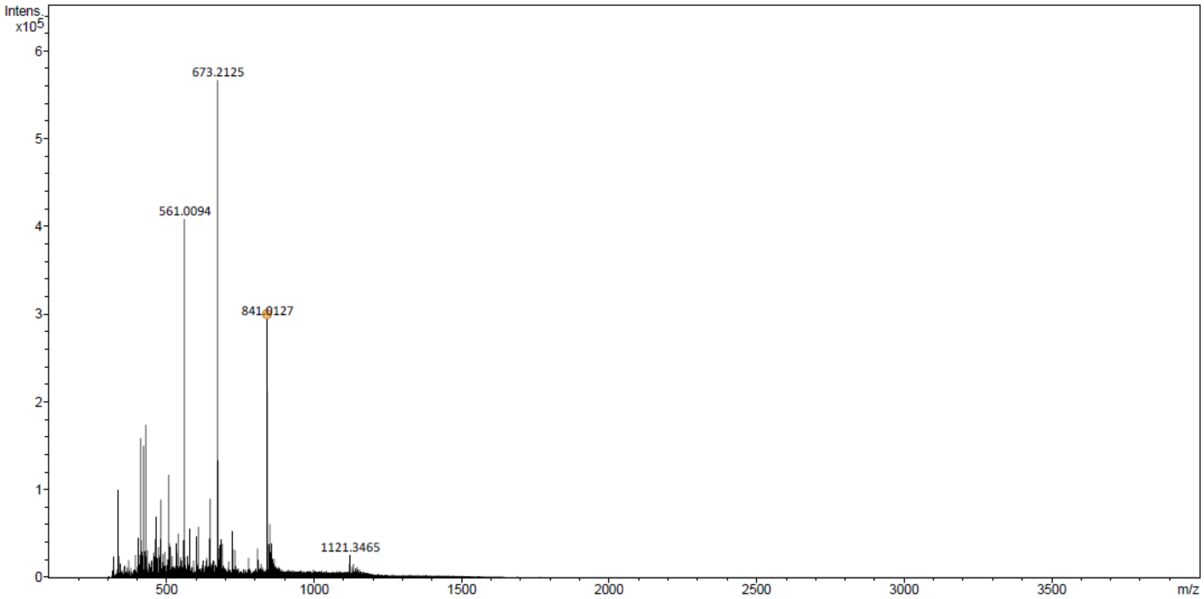

Figure S19. ESI-HRMS, m/z calculated for **1**, $[C_{151}H_{263}N_{47}O_{39}+4H]^{4+}$ 841.0103, observed 841.0127.

S.9 Synthesis of bicyclic cathelicidin-PY analogue (3) on TentaGel S NH_2 resin using flow chemistry

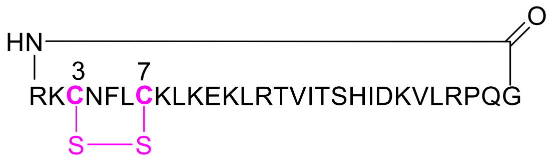

3

Method 1: Loading TentaGel® S NH_2 with HMPB linker

TentaGel® S NH_2 resin (600 mg, 0.15 mmol, 0.25 $mmolg^{-1}$) was pre-swollen in CH_2Cl_2 (5 mL, 30 min). 4-(4-Hydroxymethyl-3-methoxyphenoxy)butyric acid (HMPB linker) (180.18 mg, 0.75 mmol, 5 equiv.) and 6-Cl-HOBt (127.18 mg, 0.5 mmol, 5 equiv.) dissolved in DMF (2.0 mL) followed by addition of DIC (117.43 µl, 0.75 mmol, 5 equiv.) added to the swollen resin. The reaction mixture was gently agitated at room temperature for 3 h. The resin was filtered and washed with DMF (3 × 3 mL) after which a negative ninhydrin test confirmed successful coupling.

Method 2: Resin functionalisation for *C*-terminal acid peptides

The symmetrical anhydride of the N^{α}-Fmoc protected *C*-terminal amino acid was prepared with Fmoc-Gly-OH (445.97 mg, 1.5 mmol, 10 equiv.) by reacting with DIC (232.27 µl, 1.5 mmol, 10 equiv.) in CH_2Cl_2 for 10 mins at room temperature. The resulting symmetric anhydride (1.0 mmol, 10 equiv.) was manually esterified with the resin bound HMPA linker via acyl transfer, using a catalytic amount of DMAP (1.83 mg, 0.01 mmol, 0.1 equiv.) in DMF for 2 h at room temperature.

Method 3: General procedure for removal of N^{α}-Fmoc protecting group

The peptidyl resin was treated with a solution of 30% piperidine, 5% formic acid in DMF (40 s, 10 mL, *v/v/v*) at 65 °C and resin washed by DMF (40s, 10 mL) removing any residual capping solution.

Method 4: General procedure for amino acid coupling using HATU

The freshly prepared Fmoc-AA-OH (0.30 M, 20 equiv.) with coupling reagent HATU (1.11 g, 2.9 mol, 19.5 equiv.) was dissolved in DMF (10 mL). DIPEA (1.019 mL, 5.85 mmol, 39 equiv.) was added to pre-activate the coupling solution for 20 seconds prior to delivery to the on-resin

peptide bearing a free N^{α}-amino group. The activated amino acid solution was fully delivered (~40 s, 10 mL) and the delivery line washed by drawing a further DMF (20 s, 4 mL). The peptidyl resin was further washed with DMF, removing any residual amino coupling solution (40 s, 10 mL).

Method 5: Coupling procedures for Fmoc-Cys(Trt)-OH and Fmoc-His(Trt)-OH

Freshly prepared Fmoc-AA-OH (0.30 M, 20 equiv.) in coupling reagent PyAOP (1.525 g, 2.9 mmol, 19.5 equiv.) was dissolved in DMF (10 mL). *Sym*-collidine (761 μl, 5.85 mmol, 39 equiv.) was added to pre-activate the coupling solution 20 seconds prior to delivery to the on-resin peptide bearing the free N^{α}-amino group. The activated amino acid solution was fully delivered (~40 s, 10 mL) and the delivery line is washed by drawing a further DMF (20 s, 4 mL). The peptidyl resin is further washed with DMF, removing any residual amino coupling solution (40 s, 10 mL).

Method 6: General procedure for capping the free amino groups:

The Fmoc-protected peptidyl resin was treated every 4th coupling cycle with a solution of 20% acetic anhydride in DMF (40 s, 10 mL, *v/v*) at 65 ^{o}C and resin washed thorough by DMF (80 s, 20 mL) removing any residual capping solution.

Method 7: On-resin iodine mediated disulfide bond formation between Cys(Trt) residues

The N^{α}-Fmoc protected peptidyl resin was treated with a fine powder of iodine (114.2 mg, 0.45 mmol, 3 equiv.) dissolved in DMF and agitated batchwise (2 × 5 min) at room temperature. Following iodine treatments, the resin was thoroughly washed with DMF (3 × 5 ml) and treated with 20% piperidine in DMF (2 x 5 ml, *v/v*) to remove the N^{α}-terminal Fmoc protecting group, followed by DMF wash (3 x 5 mL).

Method 8: Acid sensitive peptidyl resin cleavage

The resin was washed with DMF (3 × 5 ml) followed by CH_2Cl_2 (3 × 5 ml) and dried under *in vacuo*. The dried resin bound peptide was treated with 20% HFIP in CH_2Cl_2 (10 ml, *v/v*) and agitated at room temperature for 2 × 1 h. The cleavage solution containing the liberated fully sidechain protected peptide was filtered from the resin and its volume reduced under N_2, to produce a colourless gum like product. Diethyl ether (45 mL) was added to collate all residual gum like product and centrifuged to produce a pellet of gum like product. The pellet was

isolated by centrifugation, washed once more with diethyl ether (45 mL), dissolved in MeCN: H_2O (1:1, 15 mL, *v/v*) and lyophilised.

Method 9: *N* to *C* terminal amide bond formation

The fully side-chain protected peptide was dissolved in DMF to yield a peptide concentration of 0.5 mM. To this mixture PyAOP (391.1 mg, 0.75 mmol, 5 equiv.) and DIPEA (1% *v/v*) were added and stirred overnight at room temperature. The solvent was removed under reduced pressure to yield the crude side-chain protected bicyclic peptide as gum.

Method 10: TFA mediated resin cleavage and global deprotection

The crude sidechain protected bicyclic peptide was treated with a mixture of TFA/H_2O/TIS (95:2.5:2.5, 10 mL, *v/v/v*) for 2 h. The filtrate was concentrated under a gentle stream of N_2 followed by the addition of cold diethyl ether to form a precipitate. The mixture was centrifuged, and the solution was carefully decanted off and discarded. The solid pellet was dissolved H_2O (20 mL) and lyophilised.

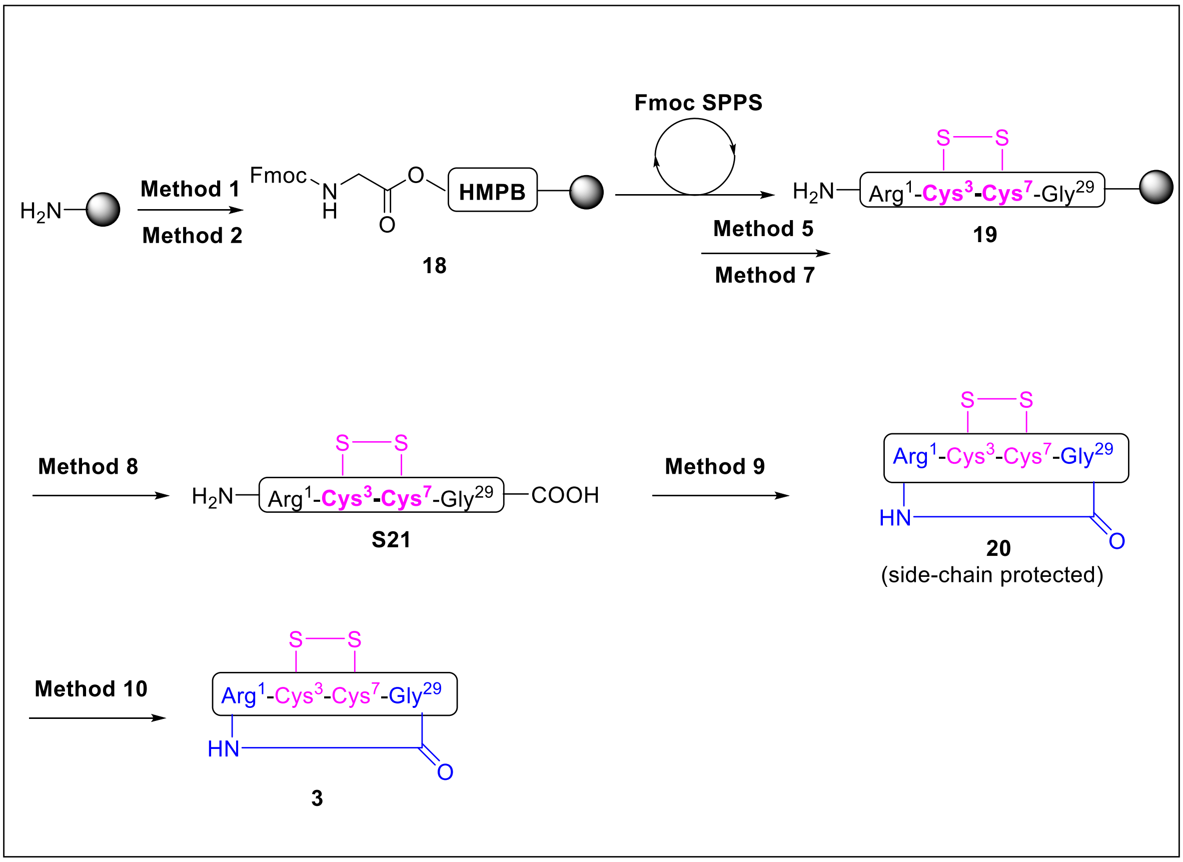

Scheme S8. Synthesis of head to tail bicyclised analogue of cathelicidin-PY peptide, **3.**

Fmoc-SPPS was used to prepare the linear sequence of cathelicidin-PY employing TentaGel S

NH_2 resin, functionaled with HMPB linker according to **Method 1**. Followed by symmetrical anhydride of the N^{α}-Fmoc protected *C*-terminal amino acid using **Method 2**. The Fmoc-Gly-HMPB linker bound resin (0.15 mmol) was packed into the metal reactor for peptide synthesis under manual flow chemistry. The manual flow synthesis was initiated by pre-washing the resin with DMF (120 s, 30mL) and performing the initial removal of the N^{α}-Fmoc protecting group using **Method 3**. The freshly made amino acids were appropriately coupled using **Method 4**, modified coupling conditions were used for cysteine and histidine residues Cys^{3}, Cys^{7} and His^{10} according to **Method 5**. Unreacted amino groups were capped using **Method 6**, followed by Fmoc-removal using **Method 3**. Once the linear cathelicidin-PY was synthesised, with the N^{α}-Fmoc protecting group still present, the peptidyl resin was treated according to **Method 7** to effect disulfide formation. The resulting monocyclic peptide was cleaved with a mildly acid cleavage cocktail according to **Method 8** to afford **S21** as a colourless gum (356.3 mg, 38.5% yield) of the fully side-chain protected crude peptide. Head to tail cyclisation was mediated according to **Method 9**. The resulting bicyclic peptide underwent global deprotection according to **Method 10** to afford **3** as a white solid (68 mg, 13.3 % yield, crude purity 18.6%). The crude peptide was purified by semi-preparative RP-HPLC using a Dionex UltiMate® 3000 on a Phenomenex Gemini C_{18} column, (10 x 150 mm, 5 mm) using a linear gradient of 10 – 60 % B over 50 min, (*ca.* 1.0% B min^{-1}) with a flow rate of 4.5 mL/min. Fractions were collected and analysed by ESI-MS for compound identification. Fractions that were identified with the correct *m/z* were collected, combined and lyophilised to afford **3** as a white amorphous solid (8.2 mg, 12.1% yield, 97.8% purity).

ESI-MS: *m/z* calculated for $[C_{151}H_{259}N_{47}O_{38}S_2+4H]^{4+}$ calculated:852.2417, observed 852.2438. **RP-HPLC:** $\mathbf{t_R}$ = 11.5 min

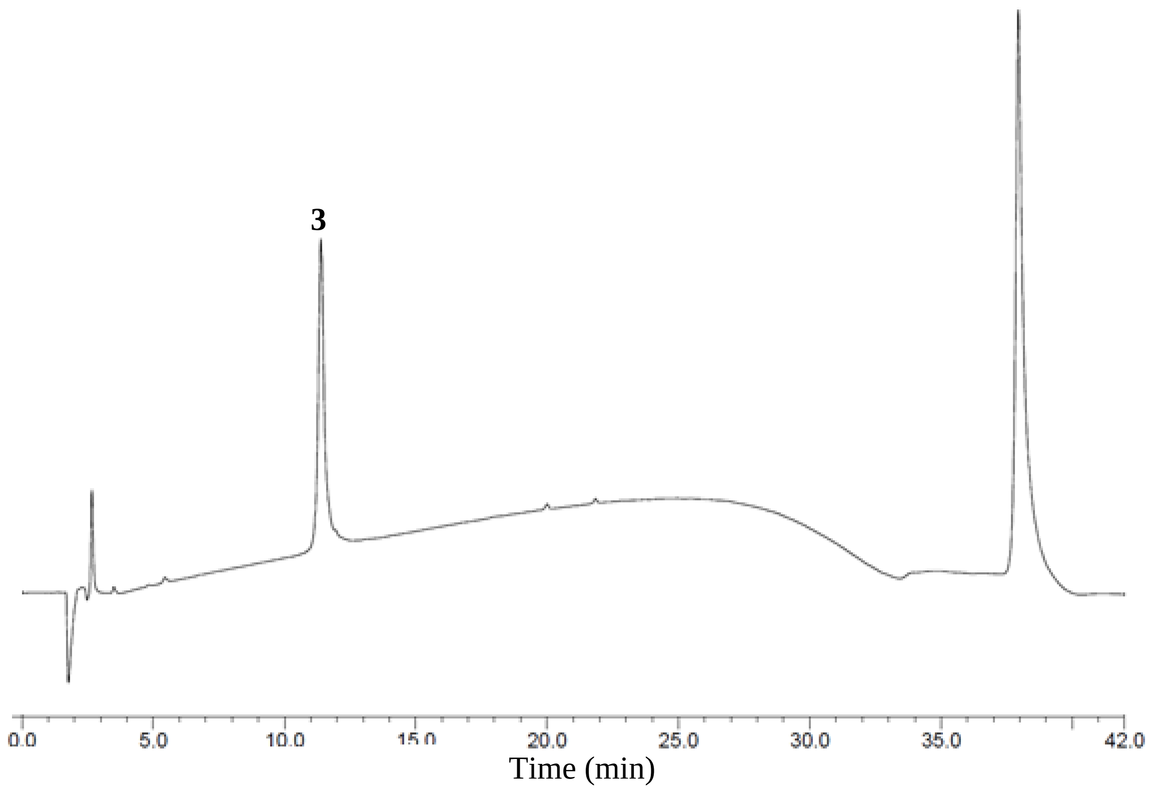

Figure S20. Analytical RP-HPLC chromatogram (214 nm) of purified head-to-tail bicyclised analogue, **3**; t_R =11.5 min. Chromatographic separations were performed on a Thermo Scientific Dionex Ultimate 3000 HPLC using a XTerra® MS C-18 column (5 µm; 4.6 × 150 mm) and a linear gradient of 10 – 50% B in 40 min at room temperature, *ca.* 1% B per min at a flow rate of 1.0 mL/min. Buffer A: H_2O containing 0.1% TFA (*v/v*); Buffer B: acetonitrile containing 0.1 % TFA (*v/v*).

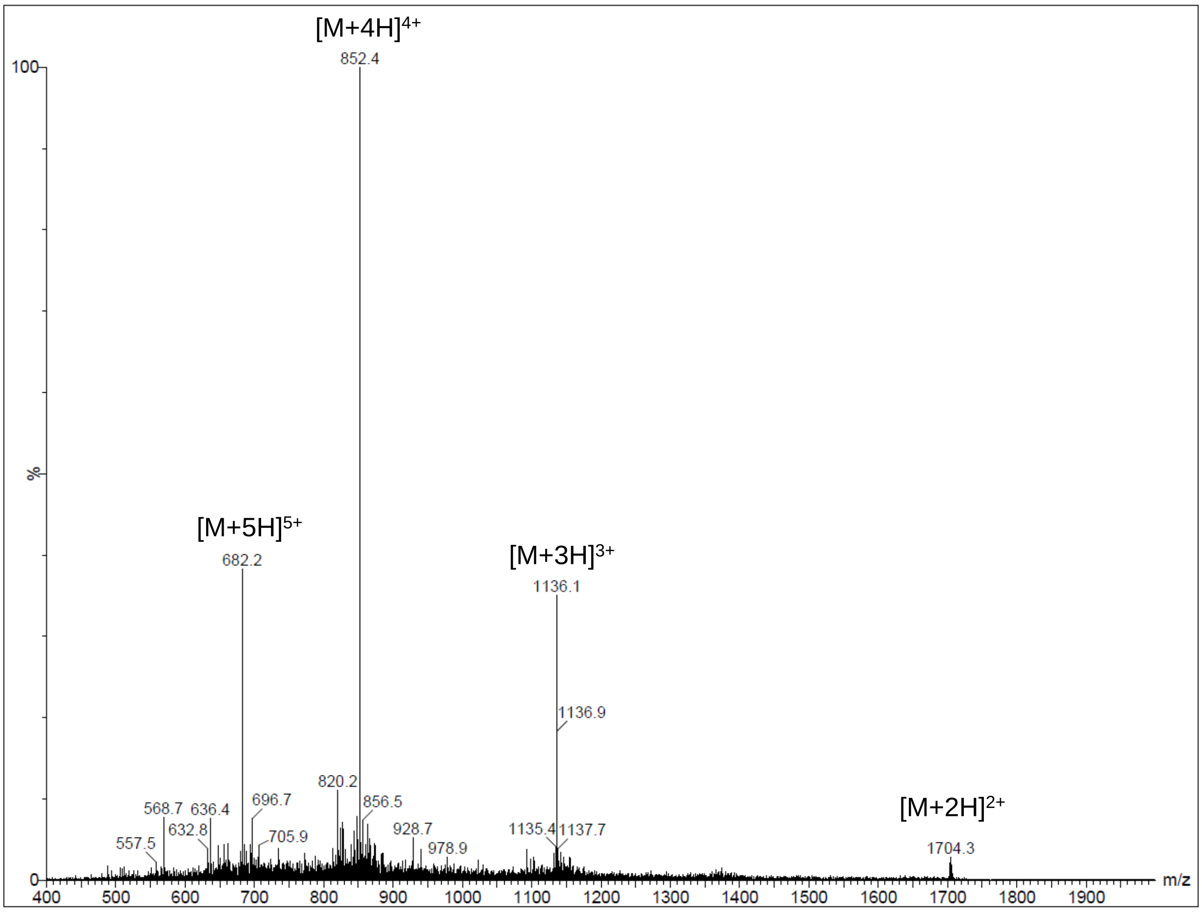

Figure S21. ESI-MS *m/z* profile of purified head-to-tail bicyclised analogue, **3**; [$C_{151}H_{259}N_{47}O_{38}S_2$] $[M+2H]^{2+}$ calculated:1702.46, observed 1704.3; $[M+3H]^{3+}$ calculated: 1135.31, observed 1136.1; $[M+4H]^{4+}$ calculated: 851.73, observed 852.4; $[M+5H]^{5+}$ calculated: 681.58, observed 682.2; Mass calculated at 3402.92 Da.

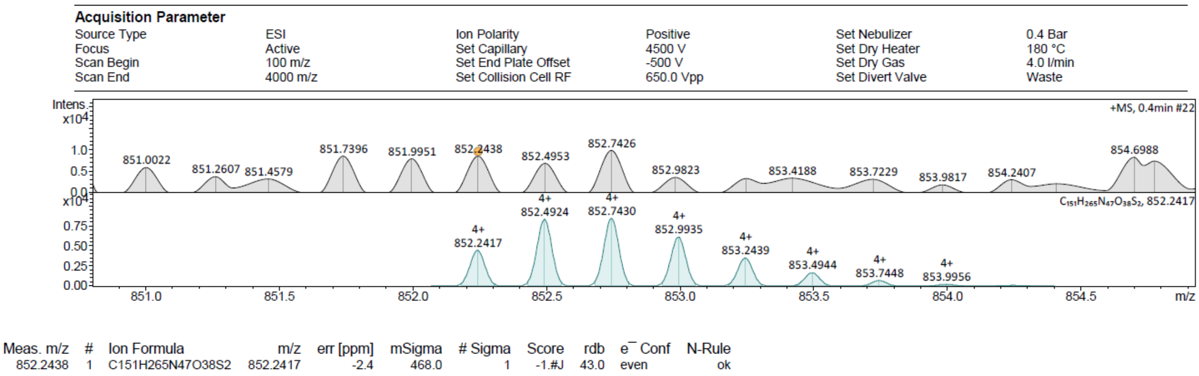

Figure S22. ESI-HRMS, formula analysis for $[C_{151}H_{259}N_{47}O_{38}S_2+4H]^{4+}$ calculated:852.2417, observed 852.2438.

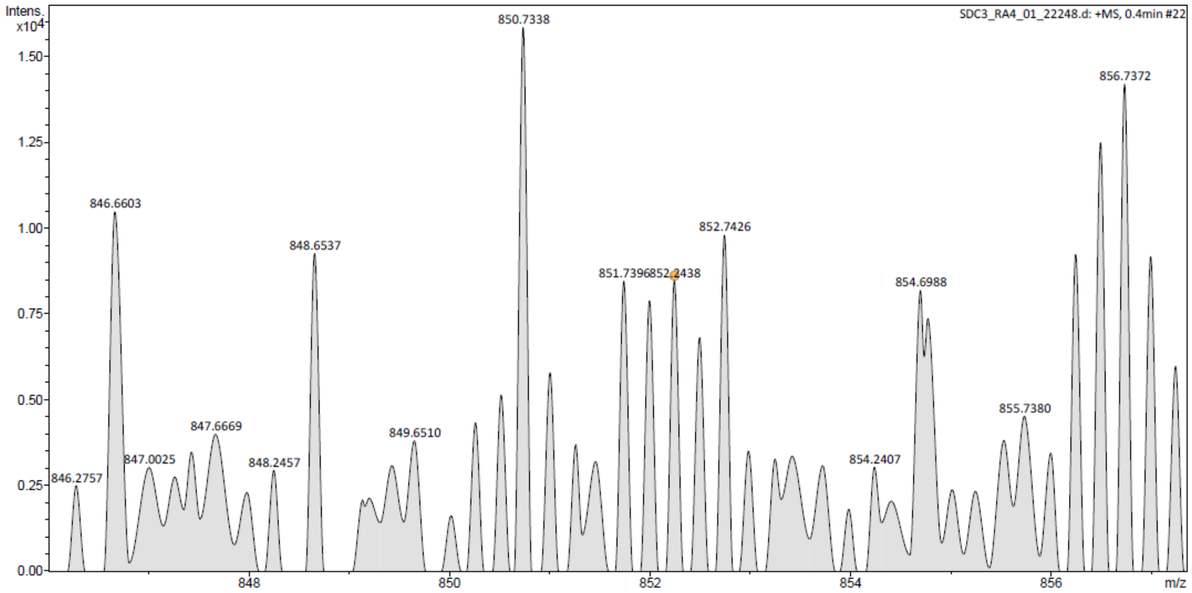

Figure S23. ESI-HRMS, m/z calculated for **3**, $[C_{151}H_{259}N_{47}O_{38}S_2{+}4H]^{4+}$ 852.2417, observed 852.2438.

S.10 Synthesis of Glu[3] / Lys[7] lactam ring mimetic (4)

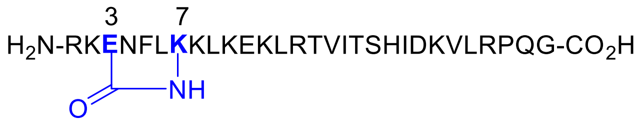

4

Method 1: Loading TentaGel® S NH_2 with HMPA linker

TentaGel® S NH_2 resin (600 mg, 0.15 mmol, 0.25 mmolg^{-1}) of particle size 130 μm was pre-swollen in CH_2Cl_2 (5 mL, 30 min). CH_2Cl_2 drained, 4-(hydroxymethyl)phenoxyacetic acid (HMPA linker) (136.64 mg, 0.75 mmol, 5 equiv.) and 6-Cl-HOBt (127.18 mg, 0.75 mmol, 5 equiv.) dissolved in DMF (2.0 mL) followed by addition of DIC (116.13 μl, 0.75 mmol, 5 equiv.) added to the swollen resin. The reaction mixture was gently agitated at room temperature for 3 hours. The resin was filtered and washed with DMF (3 × 3 mL) after which a negative ninhydrin test confirmed successful coupling.

Method 2: Resin functionalisation for *C*-terminal acid peptides

The symmetrical anhydride of the N^{α}-Fmoc protected *C*-terminal amino acid was prepared with Fmoc-Gly-OH (445.97 mg, 1.5 mmol, 10 equiv.) by reacting with DIC (232.27 μl, 1.5 mmol, 10 equiv.) in CH_2Cl_2 for 10 mins at room temperature. The resulting symmetric anhydride (1.0 mmol, 10 equiv.) was manually esterified with the resin bound HMPA linker via acyl transfer, using a catalytic amount of DMAP (1.83 mg, 0.01 mmol, 0.1 equiv.) in DMF for 2 h at room temperature.

Method 3: General procedure for removal of N^{α}-Fmoc protecting group

The peptidyl resin was treated with a solution of 30% piperidine, 5% formic acid in DMF (40 s, 10 mL, *v/v/v*) at 65 °C and resin washed by DMF (40s, 10 mL) removing any residual capping solution.

Method 4: General procedure for amino acid coupling

The freshly prepared Fmoc-AA-OH (0.30 M, 20 equiv.) with coupling reagent HATU (1.11 g, 2.9 mol, 19.5 equiv.) was dissolved in DMF (10 mL). DIPEA (1.019 mL, 5.85 mmol, 39 equiv.) was added to pre-activate the coupling solution for 20 seconds prior to delivery to the on-resin peptide bearing a free N^{α}-amino group. The activated amino acid solution was fully delivered

(~40 s, 10 mL) and the delivery line washed by drawing a further DMF (20 s, 4 mL). The peptidyl resin was further washed with DMF, removing any residual amino coupling solution (40 s, 10 mL).

Method 5: Coupling procedure for Fmoc-His(Trt)-OH

Freshly prepared Fmoc-His(Trt)-OH (1.9 g, 3.1 mmol, 20 equiv.) in coupling reagent PyAOP (1.525 g, 2.9 mmol, 19.5 equiv.) was dissolved in DMF (10 mL). *Sym*-collidine (761 µl, 5.85 mmol, 39 equiv.) was added to pre-activate the coupling solution 20 seconds prior to delivery to the on-resin peptide bearing free N^{α}-amino group. The activated amino acid solution is fully delivered (~40 s, 10 mL) and the delivery line is washed by drawing a further DMF (20 s, 4 mL). The peptidyl resin is further washed with DMF, removing any residual amino coupling solution (40 s, 10 mL).

Method 6: General procedure for capping the free amino groups:

The Fmoc-protected peptidyl resin was treated every 4th coupling cycle with a solution of 20% acetic anhydride in DMF (40 s, 10 mL, *v/v*) at 65 ^{o}C and resin washed thorough by DMF (80 s, 20 mL) removing any residual capping solution.

Method 7: General procedure for removal of 4-Methyltrityl (Mtt) and 2-phenylisopropyl (*O*-2-PhiPr) orthogonal protecting groups

Peptidyl resin was treated with 1.0% TFA in CH_2Cl_2 (10 × 3 min, *v/v*) and agitated at room temperature. At the end of 10th treatment cycle the resin was thoroughly washed with CH_2Cl_2 (5 × 5 mL) and with DMF (3 × 5 mL).

Method 8: General procedure to form lactam bridging between residue Glu^{3} and Lys^{7}

Peptidyl resin was treated with PyAOP (391 mg, 0.75 mmol, 5 equiv.) and DIPEA (130.6 µl, 0.75 mmol, 5 equiv.) in DMF (5 mL) and agitated for 4 h at room temperature. Resin thoroughly washed with DMF (3 × 5 mL).

Method 9: TFA mediated resin cleavage and global deprotection

Peptidyl resin was treated with a mixture of TFA/H_2O/TIS (95:2.5:2.5, 10 mL, *v/v/v*) for 120 min. The filtrate was concentrated under a gentle stream of N_2 followed by the addition of cold diethyl ether to form a precipitate. The mixture was centrifuged, and the solution was carefully decanted off and discarded. The solid pellet was dissolved H_2O (20 mL) and lyophilised.

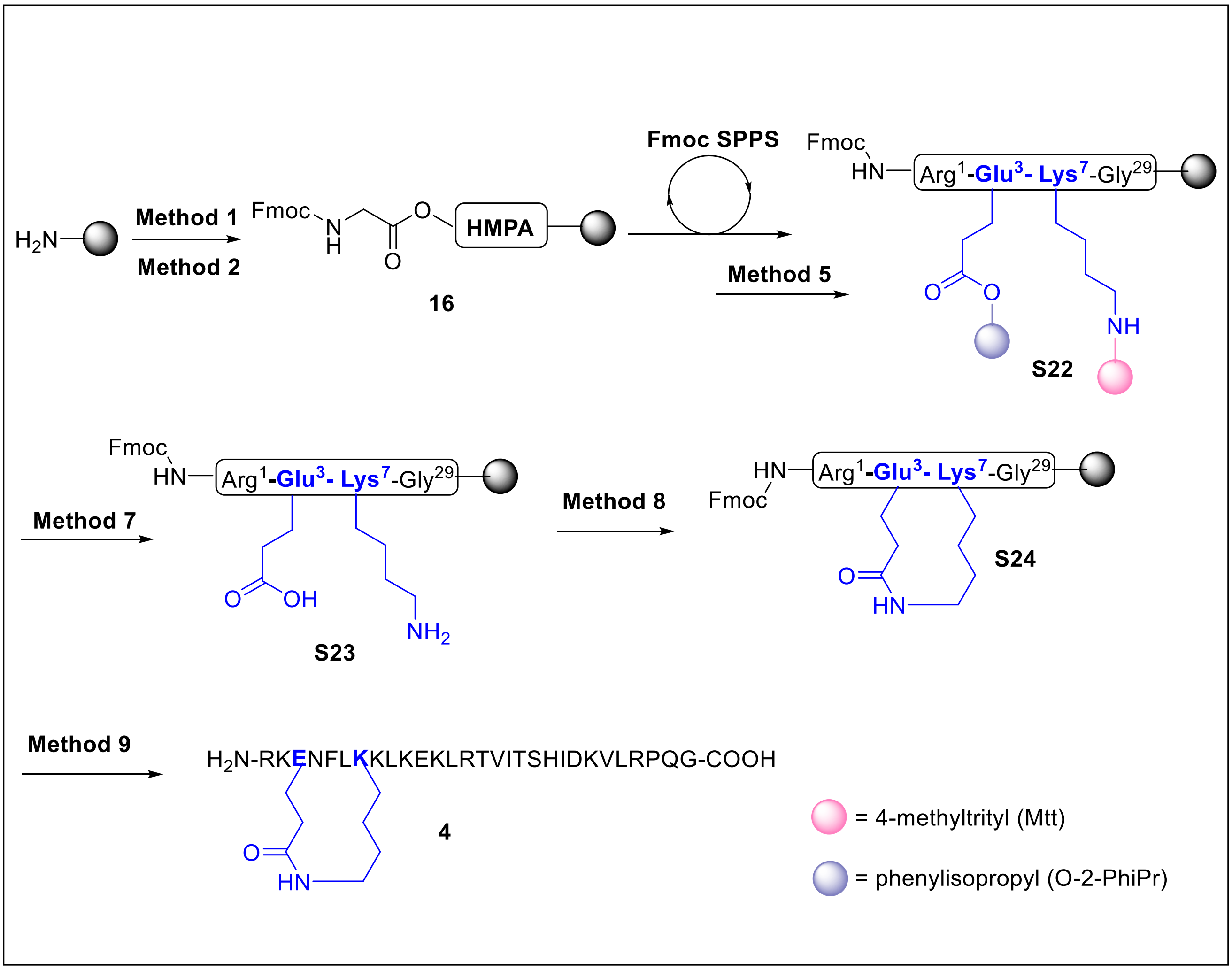

Scheme S9. Synthesis of analogue of cathelicidin-PY peptide, **4.**

Fmoc-SPPS was used for the synthesis of the linear peptide employing TentaGel S NH_2 resin, functionalizing with HMPA linker using **Method 1**. Followed coupling the symmetrical anhydride of the N^{α}-Fmoc protected *C*-terminal amino acid using **Method 2**. The Fmoc-Gly-HMPA linker bound resin (0.15 mmol) was packed into the metal reactor for peptide synthesis under manual flow chemistry. The manual flow synthesis was initiated by pre-washing the resin with DMF (120 s, 30mL) and performing the initial removal of the N^{α}-Fmoc protecting group using **Method 3**. The freshly made amino acids were appropriately coupled using **Method 4**, modified coupling conditions were used for the histidine residue, His^{10} according to **Method 5**. Unreacted amino groups were capped using **Method 6**, followed by Fmoc-removal using **Method 3**. Once the linear sequence was synthesised, with the N^{α}-Fmoc

protecting group still present, the peptidyl resin was treated with a mildly acidic cocktail to remove the orthogonal protecting group of Glu[3] and Lys[7] using **Method 7**. Side-chain-to-side-chain lactamisation was carried out according to **Method 8**. The resulting peptide was cleaved using **Method 9** to afford **4** as a white solid (363.8 mg, 65.6% yield, crude purity of 54.6%). The crude peptide was purified by semi-preparative RP-HPLC on a Phenomenex ® Gemini C18 (10 x 150 mm, 5 mm) using a linear gradient of 10 - 50% to 40 min (*ca*. 1% B/min) with a flow rate of 4.5 mL/min. Fractions were collected and analysed by ESI-MS for compound identification. Fractions identified with correct *m/z* were collected, combined and lyophilised to afford **4** as a white amorphous powder (40.75 mg, 11.2% yield, 95.8% purity. t_R = 24.58 min).

ESI-MS: *m/z* calculated for $[C_{156}H_{270}N_{48}O_{40}]^{3+}$ 1153.0; observed 1152.8. **RP-HPLC:** $\mathbf{t_R}$ = 24.6 min.

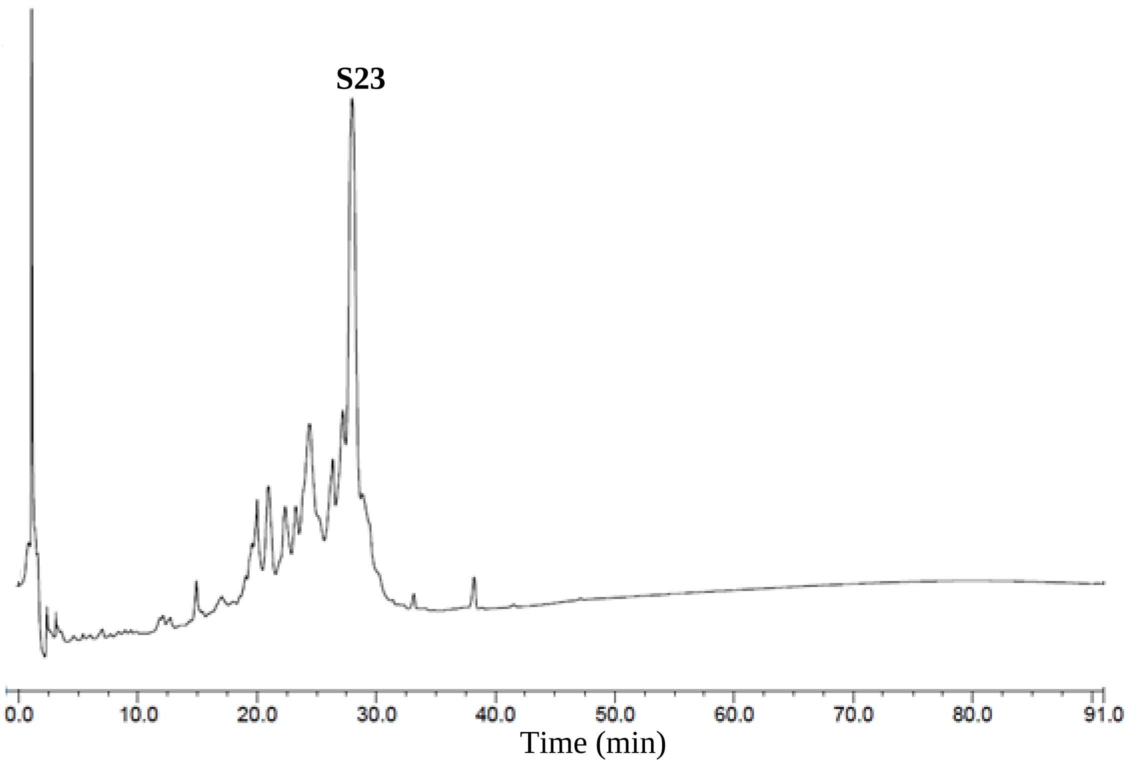

Figure S24. Analytical RP-HPLC chromatogram (214 nm) of mini cleaved crude peptide, **S23**, t_R = 28.0 min. Chromatographic separations were performed on a Thermo Scientific Dionex Ultimate 3000 HPLC using a XTerra® MS C-18 column (5 μm; 4.6 × 150 mm) and a linear gradient of 5 – 95% B in 90 min at room temperature, *ca.* 1% B per min at a flow rate of 1.0 mL/min. Buffer A: H_2O containing 0.1% TFA (*v/v*); Buffer B: acetonitrile containing 0.1 % TFA (*v/v*).

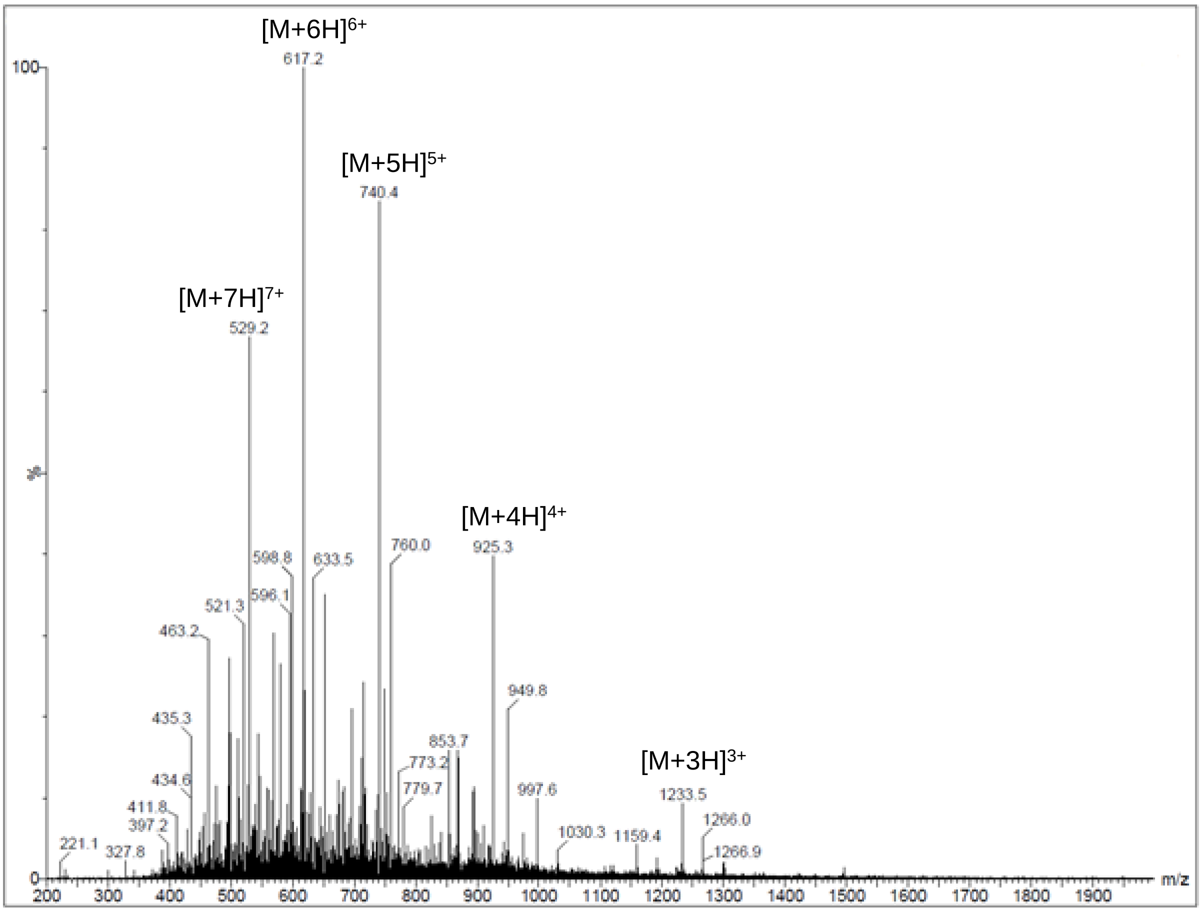

Figure S25. ESI-MS *m/z* for crude N^{α}-Fmoc protected linear peptide **S23**; [$C_{171}H_{282}N_{48}O_{43}$] $[M+3H]^{3+}$ calculated: 1233.05, observed 1233.5; $[M+4H]^{4+}$ calculated: 925.04, observed 925.3; $[M+5H]^{5+}$ calculated: 740.23, observed 740.4; $[M+6H]^{6+}$ calculated: 617.02, observed 617.2; $[M+7H]^{7+}$ calculated: 529.02, observed 529.2. Mass calculated at 3696.14 Da.

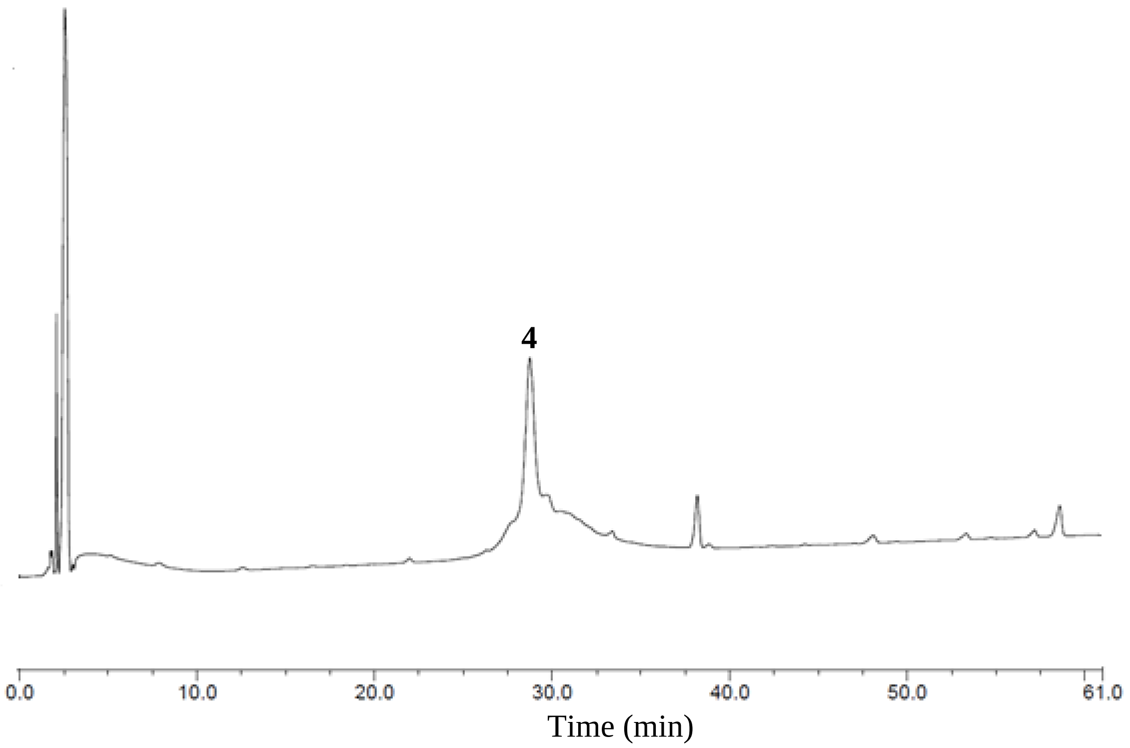

Figure S26. Analytical RP-HPLC chromatogram (214 nm) of crude peptide, **4**, t_R = 28.40 min. Chromatographic separations were performed on a Thermo Scientific Dionex Ultimate 3000 HPLC using a XTerra® MS C-18 column (5 µm; 4.6 × 150 mm) and a linear gradient of 5 – 65% B in 60 min at room temperature, *ca.* 1% B per min at a flow rate of 1.0 mL/min. Buffer A: H_2O containing 0.1% TFA (*v/v*); Buffer B: acetonitrile containing 0.1 % TFA (*v/v*).

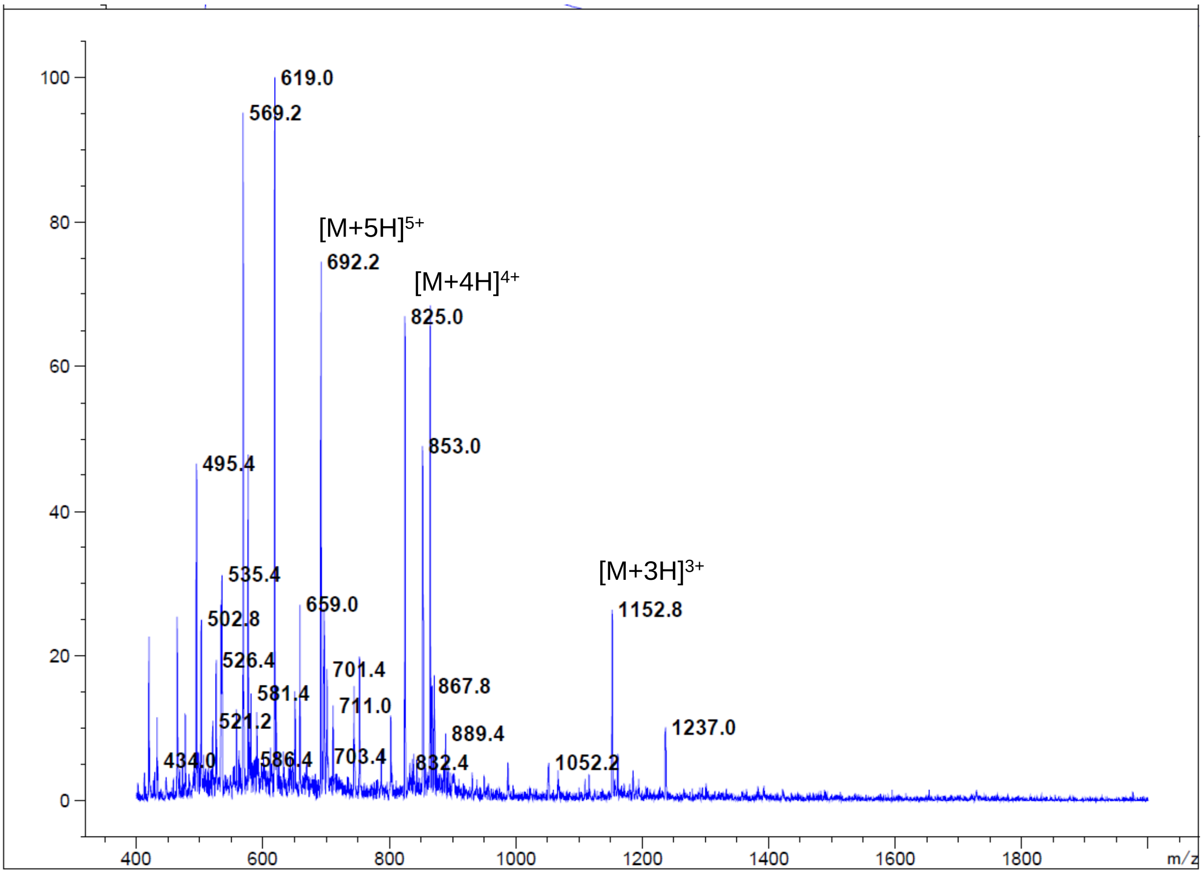

Figure S27. ESI-MS *m/z* for crude peptide **4**; [$C_{156}H_{270}N_{48}O_{40}$] $[M+3H]^{3+}$ calculated: 1153.02, observed 1152.8; $[M+4H]^{4+}$ calculated: 865.02, observed 865.3; $[M+5H]^{5+}$ calculated: 692.21, observed 692.2; Mass calculated at 3456.06 Da.

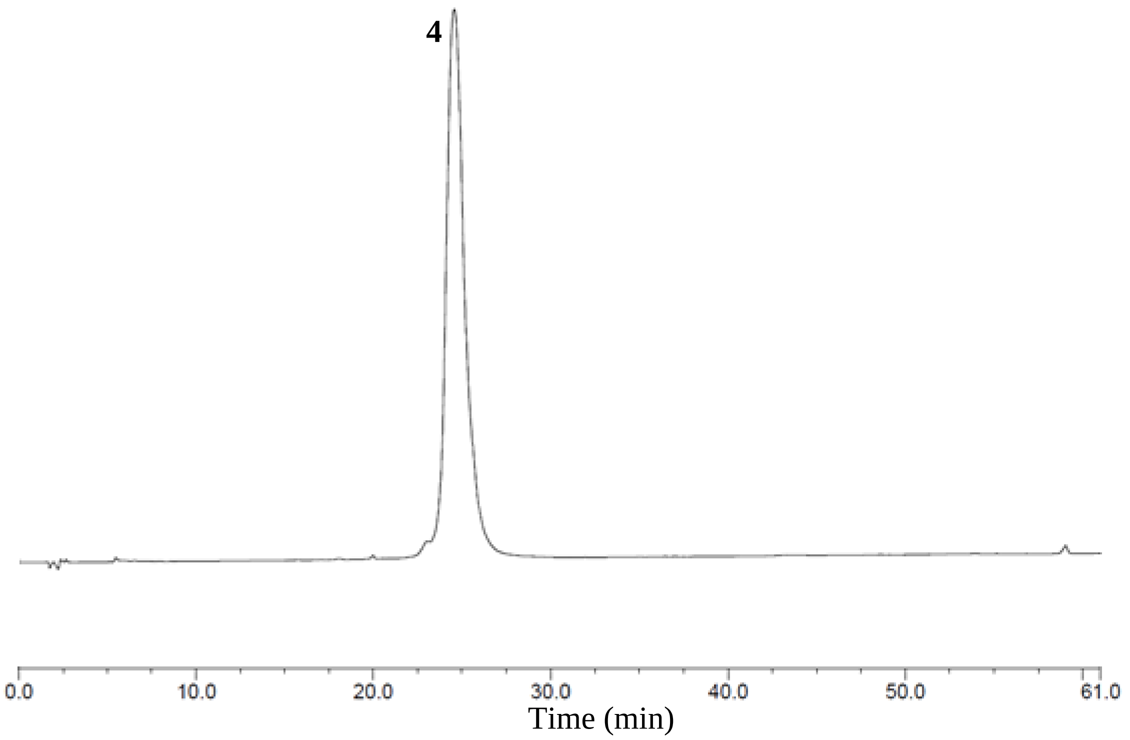

Figure S28. Analytical RP-HPLC chromatogram (214 nm) of purified peptide **4**, t_R = 24.6 min. Chromatographic separations were performed on a Thermo Scientific Dionex Ultimate 3000 HPLC using a XTerra® MS C-18 column (5 µm; 4.6 × 150 mm) and a linear gradient of 5 – 65% B in 60 min at room temperature, *ca.* 1% B per min at a flow rate of 1.0 mL/min. Buffer A: H_2O containing 0.1% TFA (*v/v*); Buffer B: acetonitrile containing 0.1 % TFA (*v/v*).

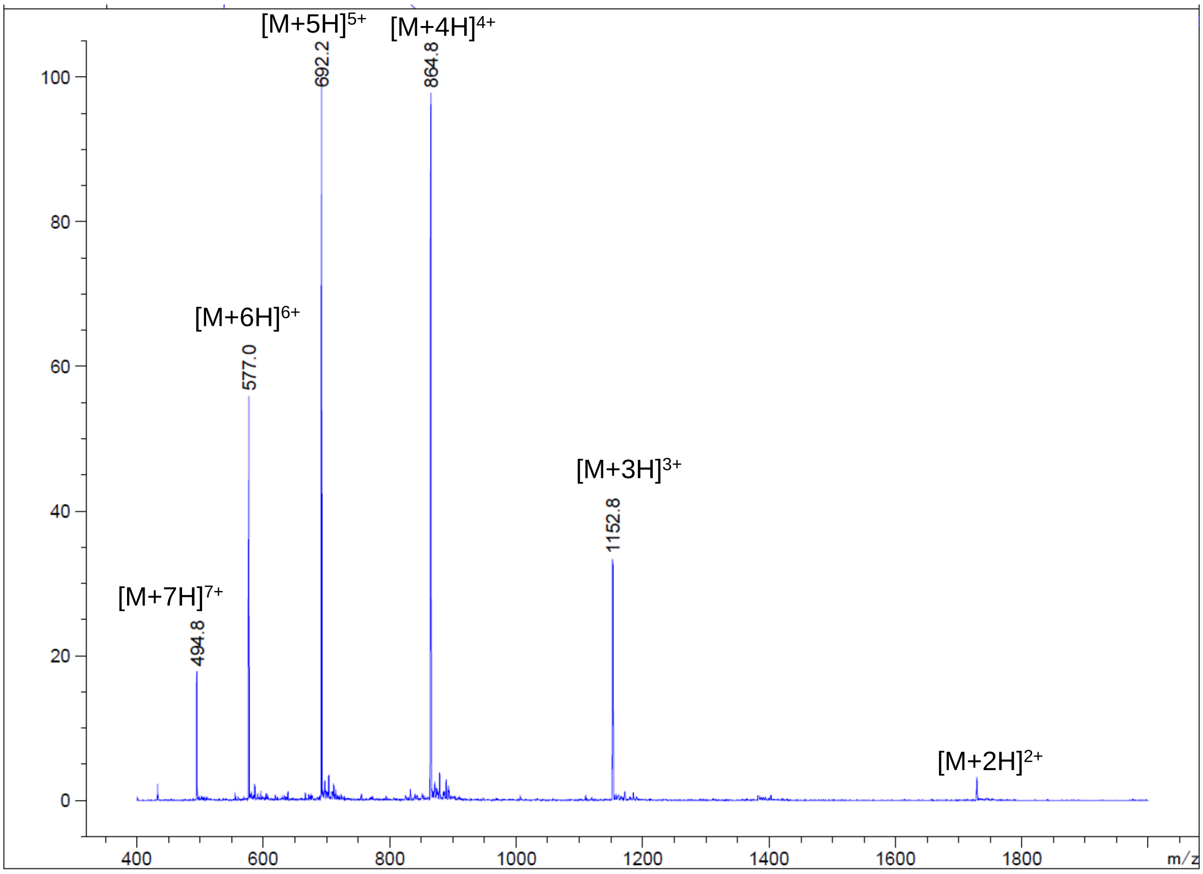

Figure S29. ESI-MS *m/z* for purified peptide **4**; [$C_{156}H_{270}N_{48}O_{40}$] $[M+2H]^{2+}$ calculated:1729.03, observed 1729.2; $[M+3H]^{3+}$ calculated: 1153.02, observed 1152.8; $[M+4H]^{4+}$ calculated: 865.02, observed 864.8; $[M+5H]^{5+}$ calculated: 692.21, observed 692.2; $[M+6H]^{6+}$ calculated: 577.01, observed 577.0; $[M+7H]^{7+}$ calculated: 494.72, observed 494.8; Mass calculated at 3456.06 Da.

S.11 Synthesis of Asp^{3} / Lys^{7} lactam ring mimetic (5)

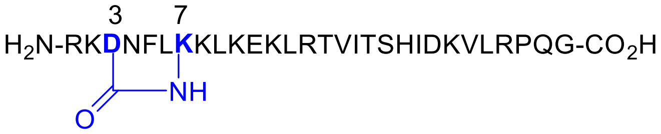

5

Method 1: Loading TentaGel® S NH$_2$ with HMPA linker

TentaGel® S NH$_2$ resin (600 mg, 0.15 mmol, 0.25 mmolg^{-1}) of particle size 130 μm was pre-swollen in CH_2Cl_2 (5 mL, 30 min). CH_2Cl_2 drained, 4-(Hydroxymethyl)phenoxyacetic acid (HMPA linker) (136.64 mg, 0.75 mmol, 5 equiv.) and *6*-Cl-HOBt (127.18 mg, 0.75 mmol, 5 equiv.) dissolved in DMF (2.0 mL) followed by addition of DIC (116.13 μl, 0.75 mmol, 5 equiv.) added to the swollen resin. The reaction mixture was gently agitated at room temperature for 3 hours. The resin was filtered and washed with DMF (3 × 3 mL) after which a negative ninhydrin test confirmed successful coupling.

Method 2: Resin functionalisation for *C*-terminal acid peptides

The symmetrical anhydride of the N$^{\alpha}$-Fmoc protected *C*-terminal amino acid was prepared with Fmoc-Gly-OH (445.97 mg, 1.5 mmol, 10 equiv.) by reacting with DIC (232.27 μl, 1.5 mmol, 10 equiv.) in CH_2Cl_2 for 10 mins at room temperature. The resulting symmetric anhydride (1.0 mmol, 10 equiv.) was manually esterified with the resin bound HMPA linker via acyl transfer, using a catalytic amount of DMAP (1.83 mg, 0.01 mmol, 0.1 equiv.) in DMF for 2 h at room temperature.

Method 3: General procedure for removal of N$^{\alpha}$-Fmoc protecting group

The peptidyl resin was treated with a solution of 30% piperidine, 5% formic acid in DMF (40 s, 10 mL, *v/v/v*) at 65 °C and resin washed by DMF (40s, 10 mL) removing any residual capping solution.

Method 4: General procedure for amino acid coupling

The freshly prepared Fmoc-AA-OH (0.30 M, 20 equiv.) with coupling reagent HATU (1.11 g, 2.9 mol, 19.5 equiv.) was dissolved in DMF (10 mL). DIPEA (1.019 mL, 5.85 mmol, 39 equiv.) was added to pre-activate the coupling solution for 20 seconds prior to delivery to the on-resin peptide bearing a free N$^{\alpha}$-amino group. The activated amino acid solution was fully delivered

(~40 s, 10 mL) and the delivery line washed by drawing a further DMF (20 s, 4 mL). The peptidyl resin was further washed with DMF, removing any residual amino coupling solution (40 s, 10 mL).

Method 5: Coupling procedures for Fmoc-His(Trt)-OH

Freshly prepared Fmoc-His(Trt)-OH (1.9 g, 3.1 mmol, 20 equiv.) in coupling reagent PyAOP (1.525 g, 2.9 mmol, 19.5 equiv.) was dissolved in DMF (10 mL). *Sym*-collidine (761 µl, 5.85 mmol, 39 equiv.) was added to pre-activate the coupling solution 20 seconds prior to delivery to the on-resin peptide bearing free N^{α}-amino group. The activated amino acid solution is fully delivered (~40 s, 10 mL) and the delivery line is washed by drawing a further DMF (20 s, 4 mL). The peptidyl resin is further washed with DMF, removing any residual amino coupling solution (40 s, 10 mL).

Method 6: General procedure for capping the free amino groups:

The Fmoc-protected peptidyl resin was treated every 4th coupling cycle with a solution of 20% acetic anhydride in DMF (40 s, 10 mL, *v/v*) at 65 ^{o}C and resin washed thorough by DMF (80 s, 20 mL) removing any residual capping solution.

Method 7: General procedure for removal of 4-Methyltrityl (Mtt) and 2-phenylisopropyl (*O*-2-PhiPr) orthogonal protecting groups

Peptidyl resin was treated with 1.0% TFA in CH_2Cl_2 (10 × 3 min, *v/v*) and agitated at room temperature. At the end of 10th treatment cycle the resin was thoroughly washed with CH_2Cl_2 (5 × 5 mL) and with DMF (3 × 5 mL).

Method 8: General procedure to form lactam bridging between residue Asp^3 and Lys^7

Peptidyl resin was treated with PyAOP (391 mg, 5 equiv.) and DIPEA (130.64 µl, 5 equiv.) in DMF (5 mL) and agitated for 4 h at room temperature. Resin thoroughly washed with DMF (3 × 5 mL).

Method 9: TFA mediated resin cleavage and global deprotection

Peptidyl resin was treated with a mixture of TFA/H_2O/TIS (95:2.5:2.5, 10 mL, *v/v/v*) for 120 min. The filtrate was concentrated under a gentle stream of N_2 followed by the addition of cold diethyl ether to form a precipitate. The mixture was centrifuged, and the solution was carefully decanted off and discarded. The solid pellet was dissolved H_2O (20 mL) and lyophilised.

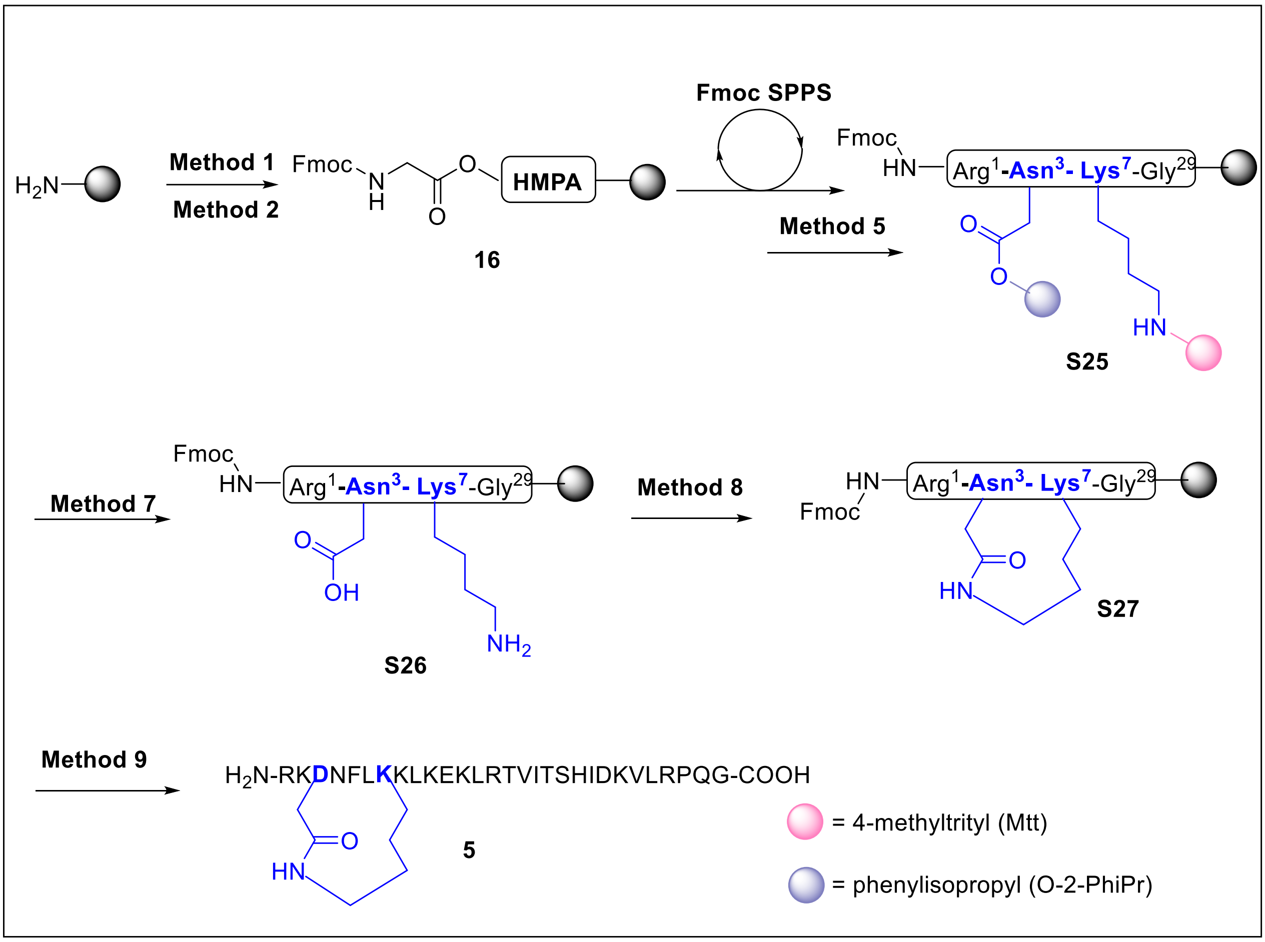

Scheme S10. Synthesis of analogue **5** using flow chemistry.

Fmoc-SPPS was used for synthesis of the linear peptide employing TentaGel S NH_2 resin, functionalizing with HMPA linker using **Method 1**. Followed by symmetrical anhydride of the N^{α}-Fmoc protected *C*-terminal amino acid using **Method 2**. The Fmoc-Gly-HMPA linker bound resin (0.15 mmol) was packed into the metal reactor for peptide synthesis under manual flow chemistry. The manual flow synthesis was initiated with pre-washing the resin with DMF (120 s, 30mL) and performing the initial removal of the N^{α}-Fmoc protecting group using **Method 3**. The freshly made amino acids were appropriately coupled using **Method 4**, modified coupling conditions were used for the histidine residue, His^{10} according to **Method**

5. Unreacted amino groups were capped using **Method 6**, followed by Fmoc-removal using **Method 3**. Once the linear cathelicidin-PY was synthesised, with the N^{α}-Fmoc protecting group still present the peptidyl resin was treated with acid sensitive cocktail to remove orthogonal protecting group off Asp^3 and Lys^7 using **Method 7**. Side-chain-to-side-chain lactam cyclisation was carried out according to **Method 8**. The resulting peptide was cleaved using **Method 9** to afford **5** as a white solid (301.5 mg, 58.4% yield, crude purity 44.6%).

The crude peptide was purified by semi-preparative RP-HPLC using a Dionex UltiMate® 3000 on a Phenomenex Gemini C_{18} column (10 x 150 mm, 5 mm) using a linear gradient of 5% to 65% over 60 min (*ca.* 1% B/min) with a flow rate of 4.5 mL/min. Fractions were collected and analysed by ESI-MS for compound identification. Fractions that were identified with the correct *m/z* were collected, combined and lyophilised to afford **5** as a white amorphous powder (32.56 mg, 10.8% yield, 98.1% purity)

ESI-MS: *m/z* calculated for $[C_{155}H_{268}N_{48}O_{40}+4H]^{4+}$ calculated:861., observed 861.7726. **RP-HPLC:** $\mathbf{t_R}$ = 24.7 min.

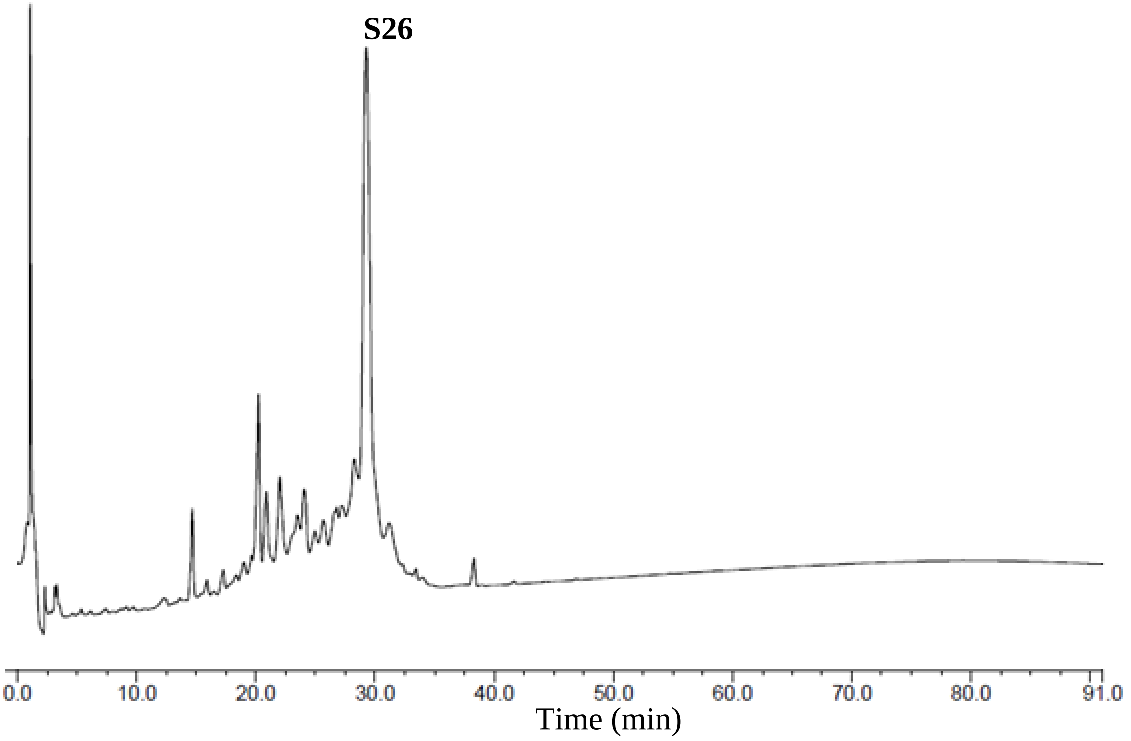

Figure S30. Analytical RP-HPLC chromatogram (214 nm) of linear peptide **S26**, t_R = 29.3 min. Chromatographic separations were performed on a Thermo Scientific Dionex Ultimate 3000 HPLC using a XTerra® MS C-18 column (5 μm; 4.6 × 150 mm) and a linear gradient of 5 - 95% B in 90 min at room temperature, *ca.* 1% B per min at a flow rate of 1.0 mL/min. Buffer A: H_2O containing 0.1% TFA (*v/v*); Buffer B: acetonitrile containing 0.1 % TFA (*v/v*).

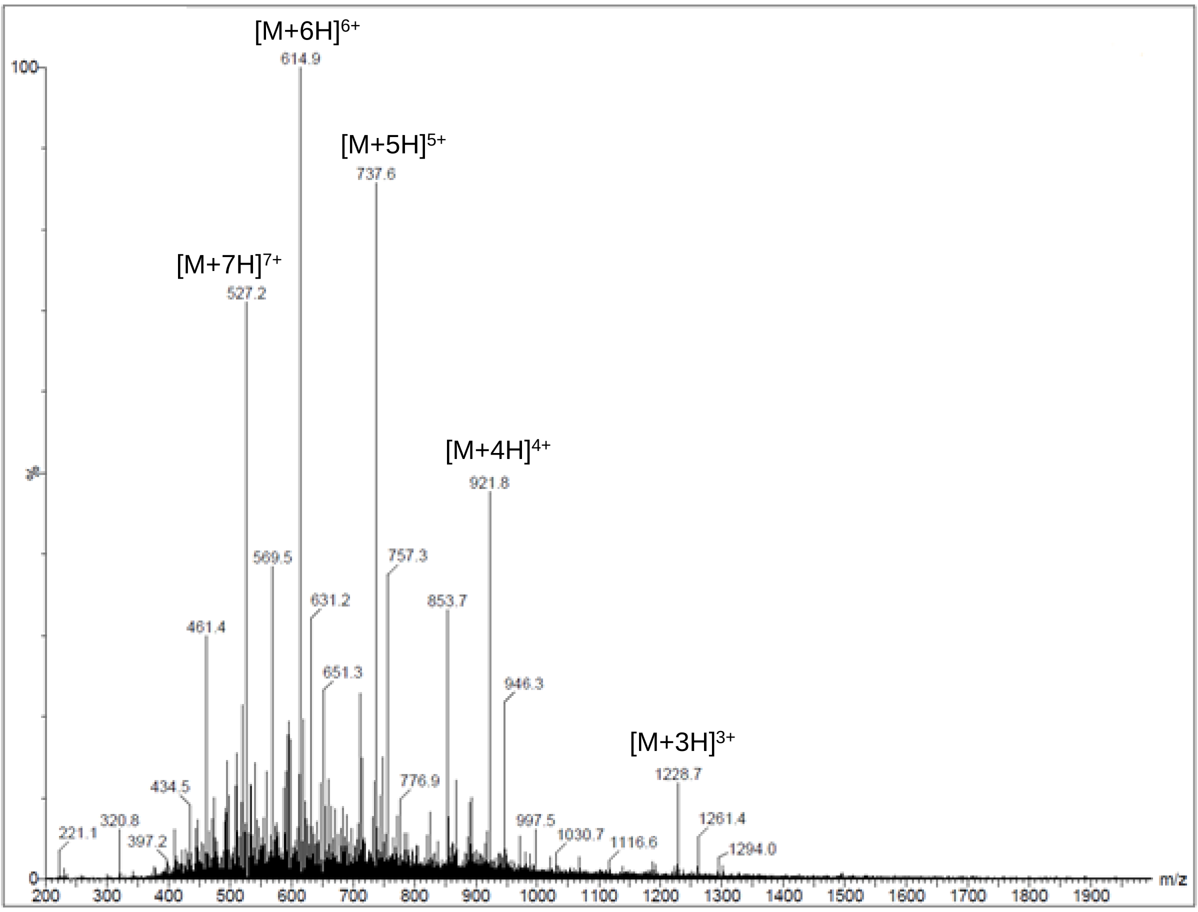

Figure S31. ESI-MS *m/z* for N$^{\alpha}$-Fmoc protected crude linear peptide **S26**; [$C_{170}H_{280}N_{48}O_{43}$] $[M+3H]^{3+}$ calculated: 1228.37, observed 1228.7; $[M+4H]^{4+}$ calculated: 921.53, observed 921.8; $[M+5H]^{5+}$ calculated: 737.42, observed 737.6; $[M+6H]^{6+}$ calculated: 614.69, observed 614.9; $[M+7H]^{7+}$ calculated: 527.02, observed 527.2. Mass calculated at 3682.12 Da.

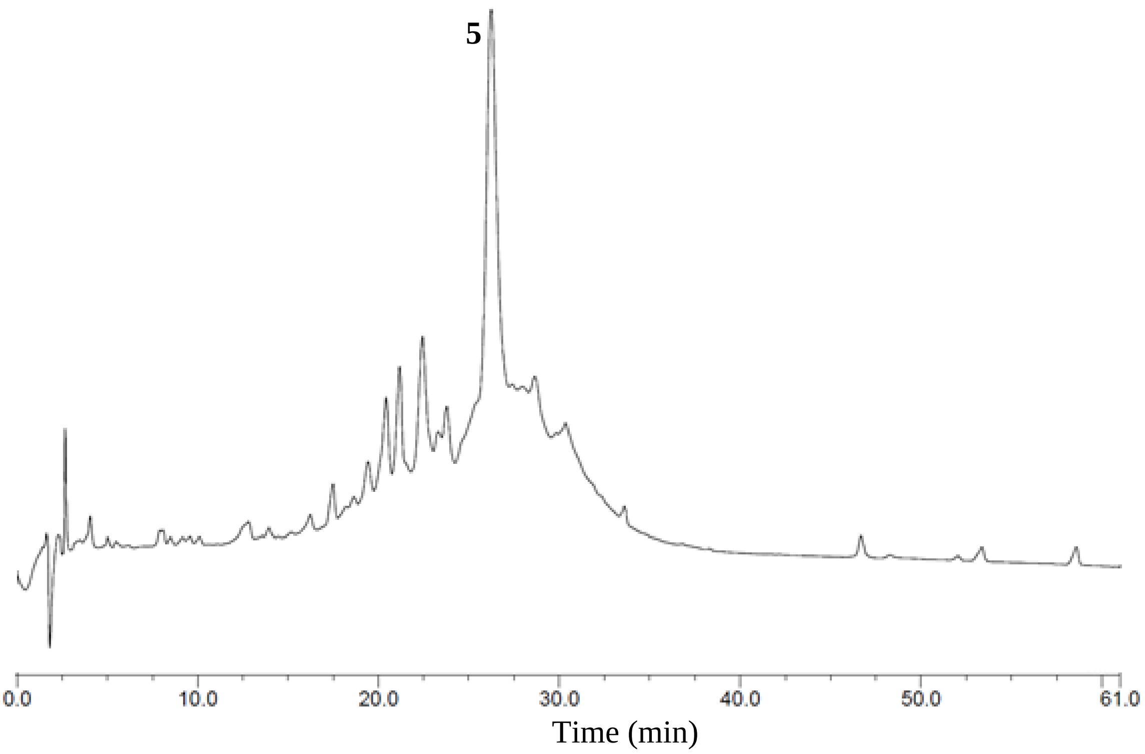

Figure S32. Analytical RP-HPLC chromatogram (214 nm) of crude peptide, **5**; t_R = 26.2 min, yield 37.8%. Chromatographic separations were performed on a Thermo Scientific Dionex Ultimate 3000 HPLC using a XTerra® MS C-18 column (5 μm; 4.6 × 150 mm) and a linear gradient of 5 – 65% B in 60 min at room temperature, *ca.* 1% B per min at a flow rate of 1.0 mL/min. Buffer A: H_2O containing 0.1% TFA (*v/v*); Buffer B: acetonitrile containing 0.1 % TFA (*v/v*).

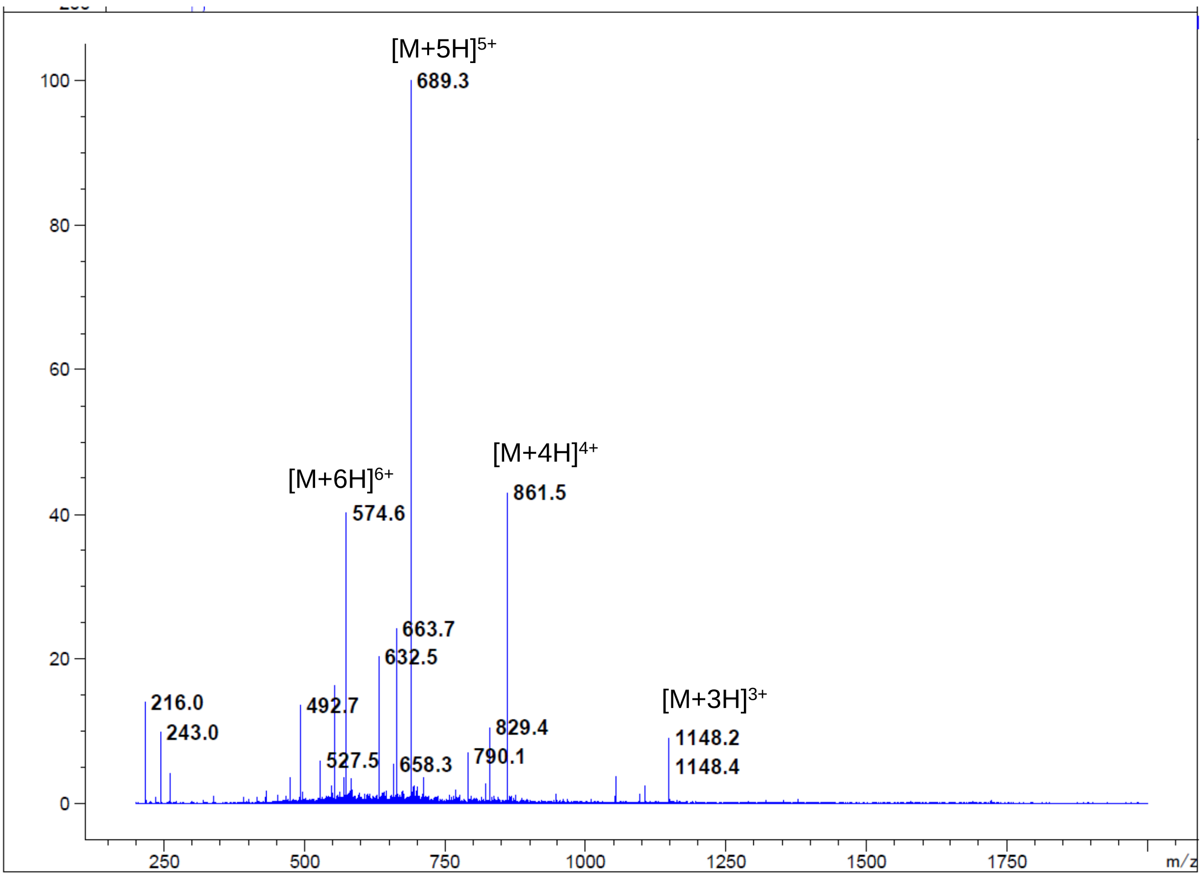

Figure S33. ESI-MS *m/z* for crude peptide, **5**; [$C_{155}H_{268}N_{48}O_{40}$] $[M+3H]^{3+}$ calculated: 1148.34, observed 1148.2; $[M+4H]^{4+}$ calculated: 861.51, observed 861.5; $[M+5H]^{5+}$ calculated: 689.41, observed 689.3; $[M+6H]^{6+}$ calculated: 574.67, observed 574.6; Mass calculated at 3442.04 Da.

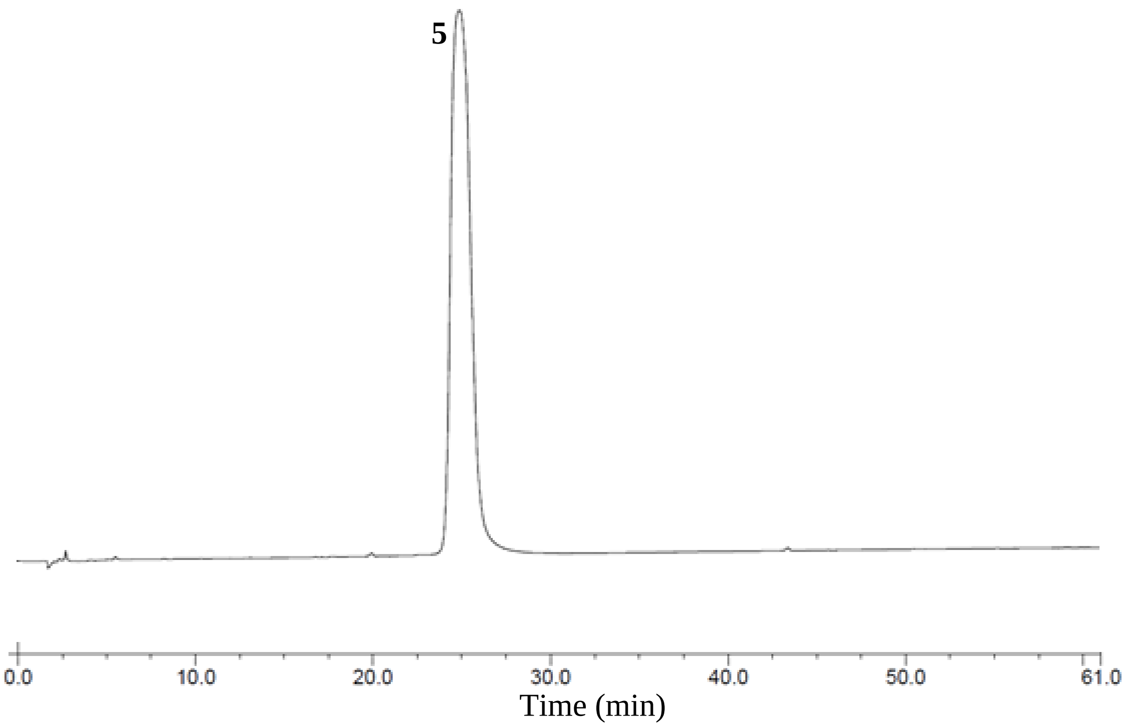

Figure S34. Analytical RP-HPLC chromatogram (214 nm) of purified peptide, **5**, R_t = 24.72 min. Chromatographic separations were performed on a Thermo Scientific Dionex Ultimate 3000 HPLC using a XTerra® MS C-18 column (5 μm; 4.6 × 150 mm) and a linear gradient of 5 – 65% B in 60 min at room temperature, *ca.* 1% B per min at a flow rate of 1.0 mL/min. Buffer A: H_2O containing 0.1% TFA (*v/v*); Buffer B: acetonitrile containing 0.1 % TFA (*v/v*).

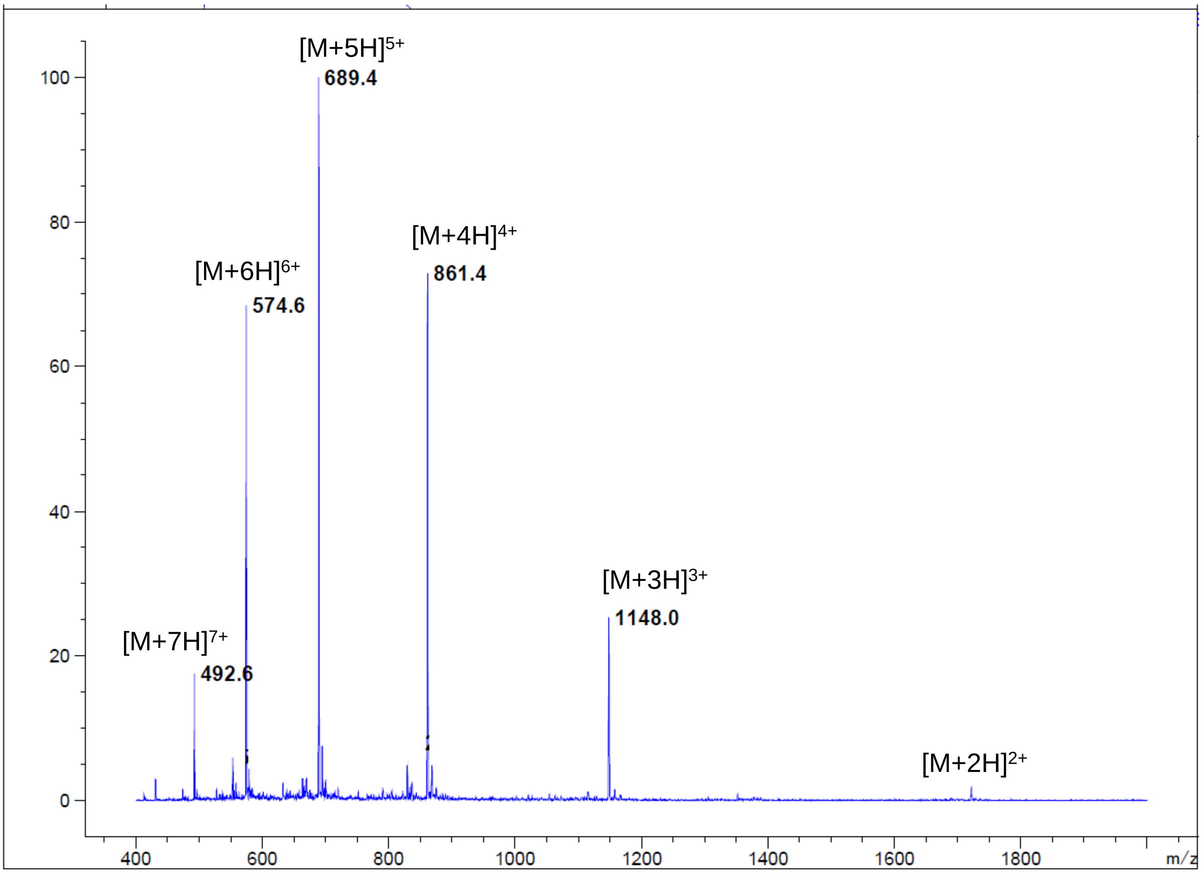

Figure S35. ESI-MS *m/z* for purified final compound, **5**; [$C_{155}H_{268}N_{48}O_{40}$] $[M+2H]^{2+}$ calculated: 1722.02, observed 1722.4; $[M+3H]^{3+}$ calculated: 1148.34, observed 1148.0; $[M+4H]^{4+}$ calculated: 861.51, observed 861.5; $[M+5H]^{5+}$ calculated: 689.41, observed 689.4; $[M+6H]^{6+}$ calculated: 574.67, observed 574.6; $[M+7H]^{7+}$ calculated: 492.72, observed 492.6; Mass calculated at 3442.04 Da.

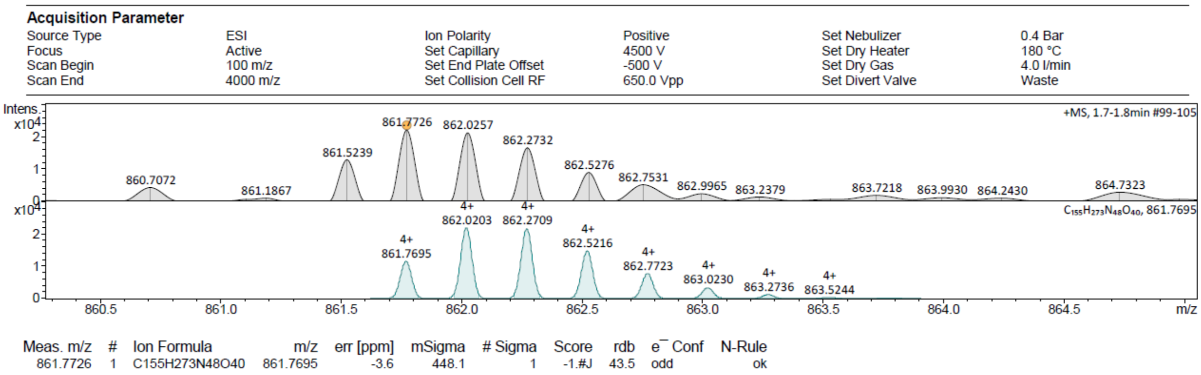

Figure S36. ESI-HRMS, formula analysis for $[C_{155}H_{268}N_{48}O_{40}+4H]^{4+}$ calculated:861.7695, observed 861.7726.

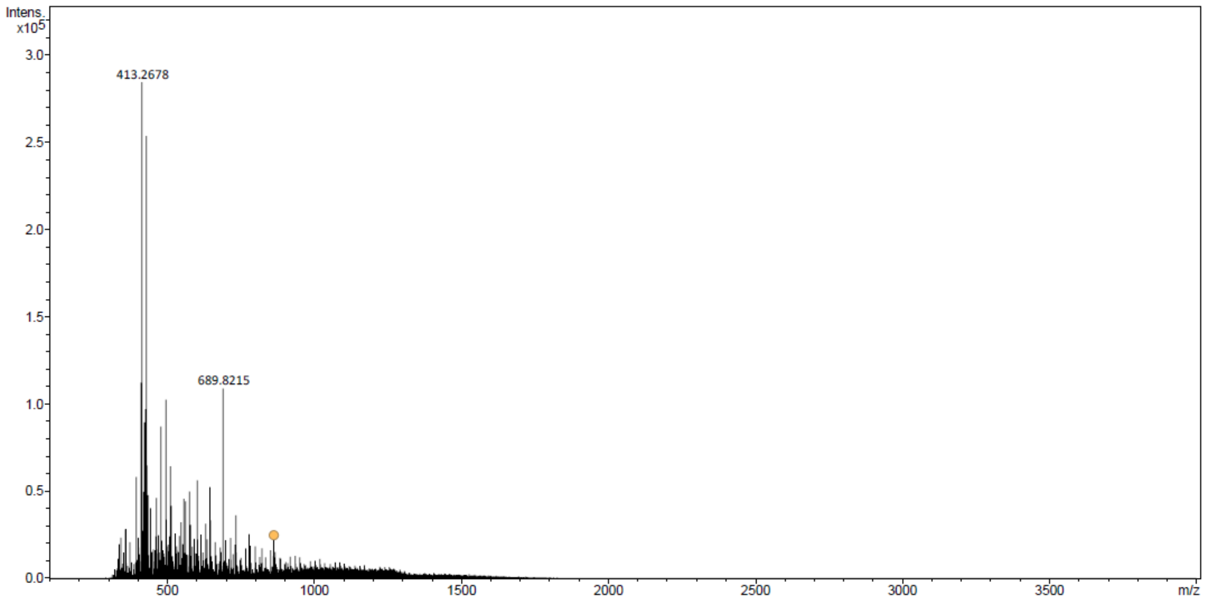

Figure S37. ESI-HRMS, *m/z* calculated for, 5 $[C_{155}H_{268}N_{48}O_{40}+4H]^{4+}$ 861.7695, observed 861.7726.

S.12 Synthesis of Lys[3] / Glu[7] lactam ring mimetic (6)

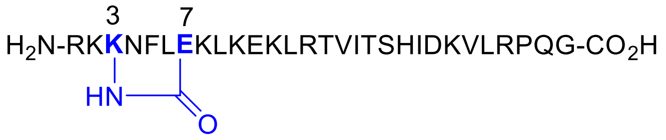

6

Method 1: Loading TentaGel® S NH_2 with HMPA linker

TentaGel® S NH_2 resin (600 mg, 0.15 mmol, 0.25 mmolg^{-1}) was pre-swollen in CH_2Cl_2 (5 mL, 30 min). CH_2Cl_2 drained, 4-(hydroxymethyl)phenoxyacetic acid (HMPA linker) (136.64 mg, 0.5 mmol, 5 equiv.) and 6-Cl-HOBt (127.18 mg, 0.5 mmol, 5 equiv.) dissolved in DMF (2.0 mL) followed by addition of DIC (117.43 µl, 0.5 mmol, 5 equiv.) added to the swollen resin. The reaction mixture was gently agitated at room temperature for 3 hours. The resin was filtered and washed with DMF (3 × 3 mL) after which a negative ninhydrin test confirmed successful coupling.

Method 2: Resin functionalisation for *C*-terminal acid peptides

The symmetrical anhydride of the N$^{\alpha}$-Fmoc protected *C*-terminal amino acid was prepared with Fmoc-Gly-OH (445.97 mg, 1.0 mmol, 10 equiv.) by reacting with DIC (117.43 µl, 0.5mmol, 5 equiv.) in CH_2Cl_2 for 10 mins at room temperature. The resulting symmetric anhydride (0.5 mmol, 5 equiv.) was manually esterified with the resin bound HMPA linker via acyl transfer, using a catalytic amount of DMAP (1.32 mg, 0.01 mmol, 0.1 equiv.) in DMF for 2 h at room temperature.

Method 3: General procedure for removal of N$^{\alpha}$-Fmoc protecting group

The peptidyl resin was treated with a solution of 30% piperidine, 5% formic acid in DMF (40 s, 10 mL, *v/v/v*) at 65 °C and resin washed by DMF (40s, 10 mL) removing any residual capping solution.

Method 4: General procedure for amino acid coupling using HATU

The freshly prepared amino acid (0.30 M, 20 equiv.) with coupling reagent HATU (0.28 M, 19.5 equiv.) was dissolved in DMF (10 mL). DIPEA (1358.7 µL, 6.0 mmol, 39 equiv.) was added to pre-activate the coupling solution 20 seconds prior to delivery to the on-resin peptide bearing free N$^{\alpha}$-amino group. The activated amino acid solution is fully delivered (~40 s, 10

mL) and the delivery line is washed by drawing a further DMF (20 s, 4 mL). The peptidyl resin is further washed with DMF, removing any residual amino coupling solution (40 s, 10 mL).

Method 5: Coupling procedure for Fmoc-His(Trt)-OH

Freshly prepared Fmoc-His(Trt)-OH (1.859 g, 20 equiv.) in coupling reagent PyAOP (1.525 g, 2.9 mmol, 19.5 equiv.) was dissolved in DMF (10 mL). *Sym*-collidine (779 µl, 39 equiv.) was added to pre-activate the coupling solution 20 seconds prior to delivery to the on-resin peptide bearing free N^{α}-amino group. The activated amino acid solution is fully delivered (~40 s, 10 mL) and the delivery line is washed by drawing a further DMF (20 s, 4 mL). The peptidyl resin is further washed with DMF, removing any residual amino coupling solution (40 s, 10 mL).

Method 6: General procedure for capping the free amino groups:

Fmoc-protected peptidyl resin was treated with a solution of 20% acetic anhydride in DMF (40 s, 10 mL, *v/v*) at 65 ^{o}C and resin washed thorough by DMF (80s, 20 mL) removing any residual capping solution.

Method 7: General procedure for removal of 4-Methyltrityl (Mtt) and 2-phenylisopropyl (*O*-2-PhiPr) orthogonal protecting groups

Peptidyl resin was treated with 1.0% TFA in CH_2Cl_2 (10 × 3 min, *v/v*) and agitated at room temperature. At the end of 10th treatment cycle the resin was thoroughly washed with CH_2Cl_2 (5 × 5 mL) and with DMF (3 × 5 mL).

Method 8: General procedure to form lactam bridging between residue Lys^{3} and Glu^{7}

Peptidyl resin was treated with PyAOP (391 mg, 5 equiv.) and DIPEA (130.64 µl, 5 equiv.) in DMF (5 mL) and agitated for 4 h at room temperature.

Method 9: TFA mediated resin cleavage and global deprotection

Peptidyl resin was treated with a mixture of TFA/H_2O/TIS (95:2.5:2.5, 10 mL, *v/v/v*) for 120 min. The filtrate was concentrated under a gentle stream of N_2 followed by the addition of cold diethyl ether to form a precipitate. The mixture was centrifuged, and the solution was carefully decanted off and discarded. The solid pellet was dissolved H_2O (20 mL) and lyophilised.

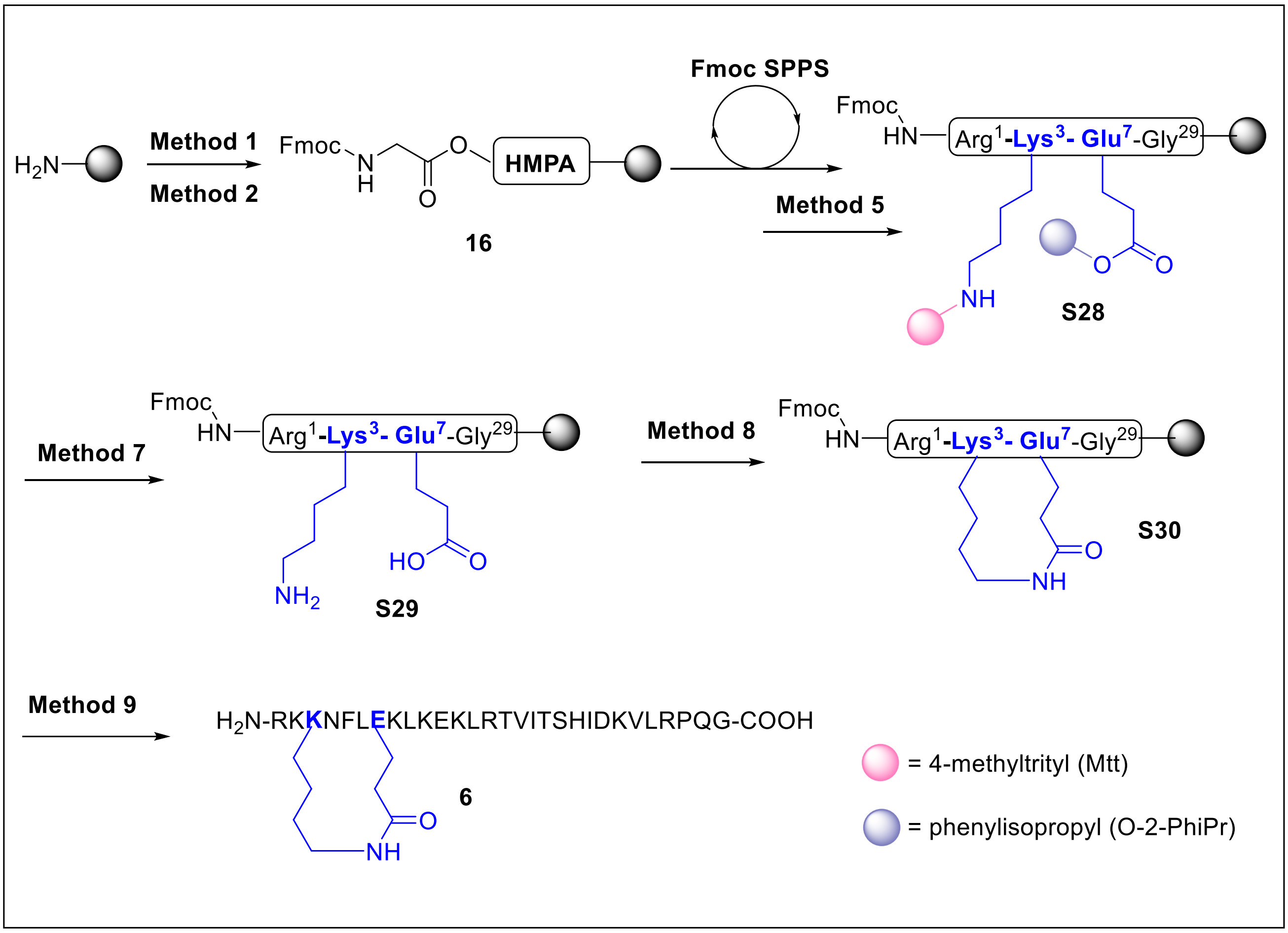

Scheme S11. Synthesis of analogues **6** using flow chemistry.

Fmoc-SPPS was used for the linear peptide synthesis employing TentaGel S NH_2 resin, functionalizing with HMPA linker using **Method 1**. Followed by symmetrical anhydride of the N^{α}-Fmoc protected *C*-terminal amino acid using **Method 2**. To NH_2-Gly-HMPA linker bound resin (0.15 mmol) was packed into the metal reactor for peptide synthesis under manual flow chemistry. The manual flow synthesis was initiated with pre-washing the resin with DMF (120 s, 30mL) and performing the initial removal of N^{α}-Fmoc protecting group using **Method 3**. The freshly made amino acids were appropriately coupled using **Method 4**, modified coupling conditions were used for histidine residue, His^{10} according to **Method 5**. Unreacted amino groups were capped using **Method 6**, followed by Fmoc-removal using **Method 3**. Once the linear cathelicidin-PY was synthesised, with the N^{α}-Fmoc protecting group still present the peptidyl resin was treated with acid sensitive cocktail to remove orthogonal protecting group of Lys3 and Glu7 using **Method 7**. Side-chain-to-side-chain lactam cyclisation was carried out according to **Method 8**. The resulting peptide was cleaved using **Method 9** to afford **6** as a white solid (373.4 mg, 67.7% yield, crude purity 43.8%). The crude peptide was

purified by semi-preparative RP-HPLC using a Dionex UltiMate® 3000 on a Phenomenex Gemini C_{18} column (10 x 150 mm, 5 mm) using a linear gradient of 5% to 65% over 60 min (*ca*. 1% B/min) with a flow rate of 4.5 mL/min. Fractions were collected and analysed by ESI-MS for compound identification. Fractions that were identified with the correct *m/z* were collected, combined and lyophilised to afford **6** as a white amorphous powder (33.97 mg, yield 9.1%, 98.8% purity).

ESI-MS: *m/z* calculated for $[C_{156}H_{270}N_{48}O_{40}+4H]^{4+}$ calculated: 865.5254 observed 865.5268. **RP-HPLC:** $\mathbf{t_R}$ = 29.2 min.

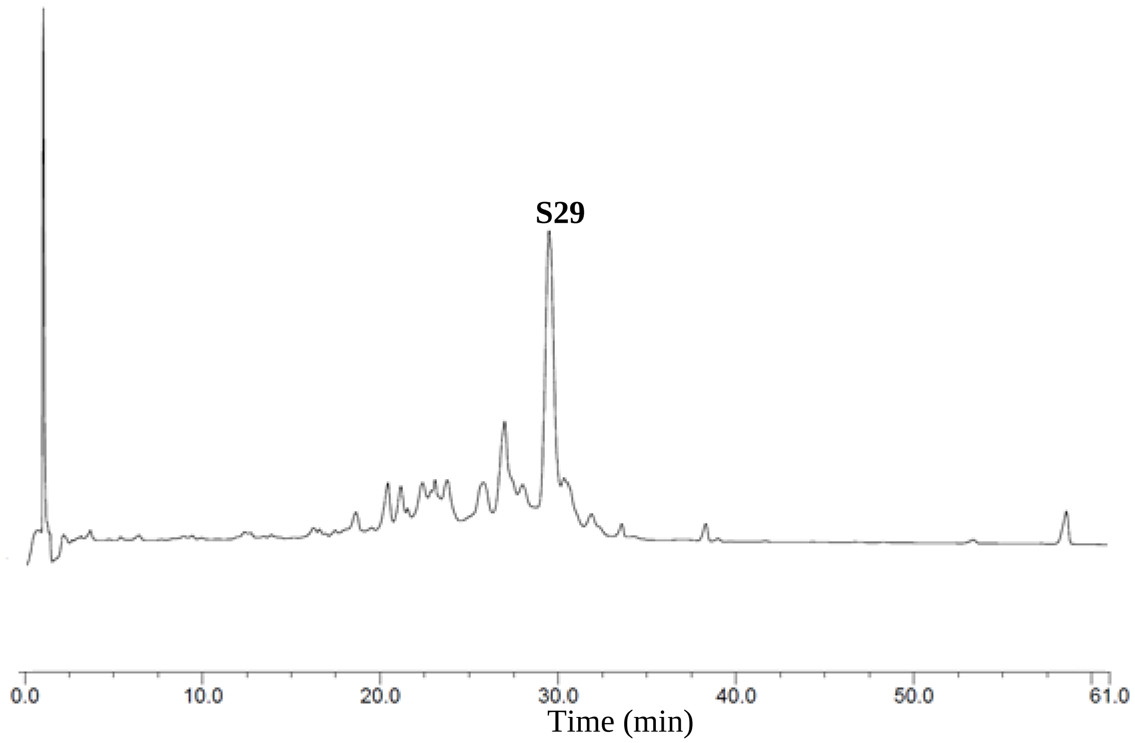

Figure S38. Analytical RP-HPLC chromatogram (214 nm) of crude linear peptide **S29**, t_R = 29.6 min, yield 14.2%. Chromatographic separations were performed on a Thermo Scientific Dionex Ultimate 3000 HPLC using a XTerra® MS C-18 column (5 μm; 4.6 × 150 mm) and a linear gradient of 5 – 65% B in 60 min at room temperature, *ca.* 1% B per min at a flow rate of 1.0 mL/min.uffer A: H_2O containing 0.1% TFA (*v/v*); Buffer B: acetonitrile containing 0.1 % TFA (*v/v*).

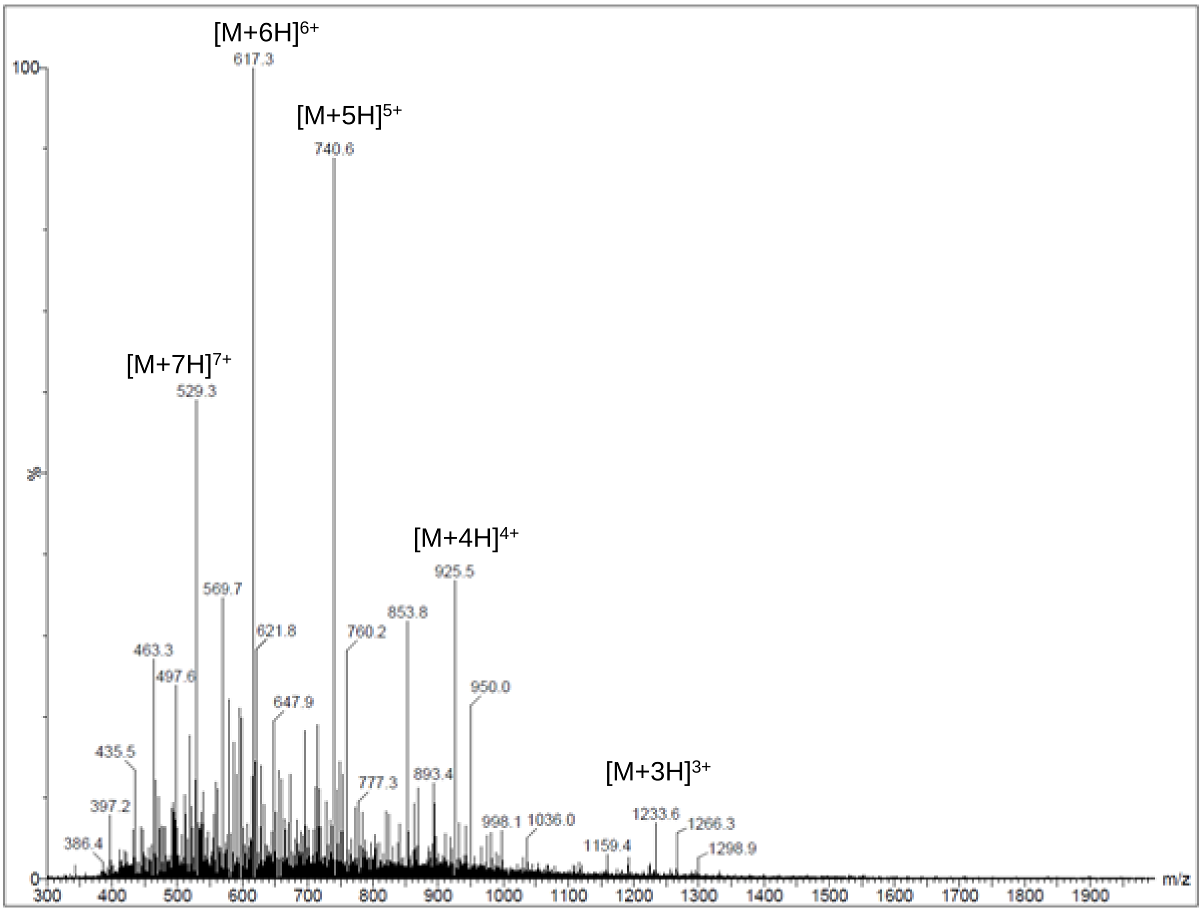

Figure S39. ESI-MS *m/z* for N$^{\alpha}$-Fmoc protected crude linear peptide **S29**; [$C_{171}H_{282}N_{48}O_{40}$] $[M+3H]^{3+}$ calculated: 1233.05, observed 1233.6; $[M+4H]^{4+}$ calculated: 925.04, observed 925.5; $[M+5H]^{5+}$ calculated: 740.23, observed 740.6; $[M+6H]^{6+}$ calculated: 617.02, observed 617.3; $[M+7H]^{7+}$ calculated 529.02:, observed 529.3. Mass calculated at 3696.14 Da.

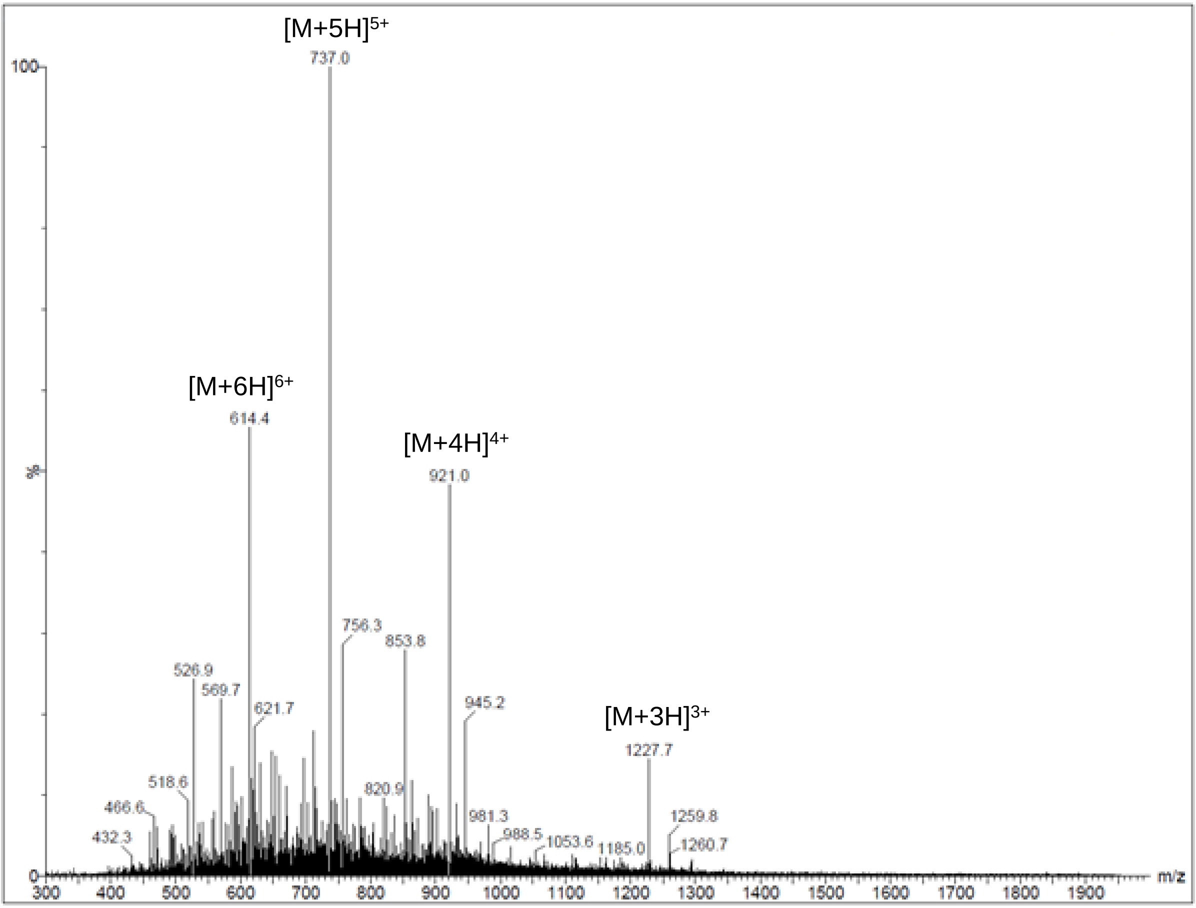

Figure S40. ESI-MS *m/z* for N$^{\alpha}$-Fmoc protected cyclised crude peptide, **S30**; [$C_{171}H_{280}N_{48}O_{42}$] $[M+3H]^{3+}$ calculated: 1227.04, observed 1227.7; $[M+4H]^{4+}$ calculated: 920.53, observed 921.0; $[M+5H]^{5+}$ calculated: 736.62, observed 737.0; $[M+6H]^{6+}$ calculated: 614.02, observed 614.4; Mass calculated at 3678.12 Da.

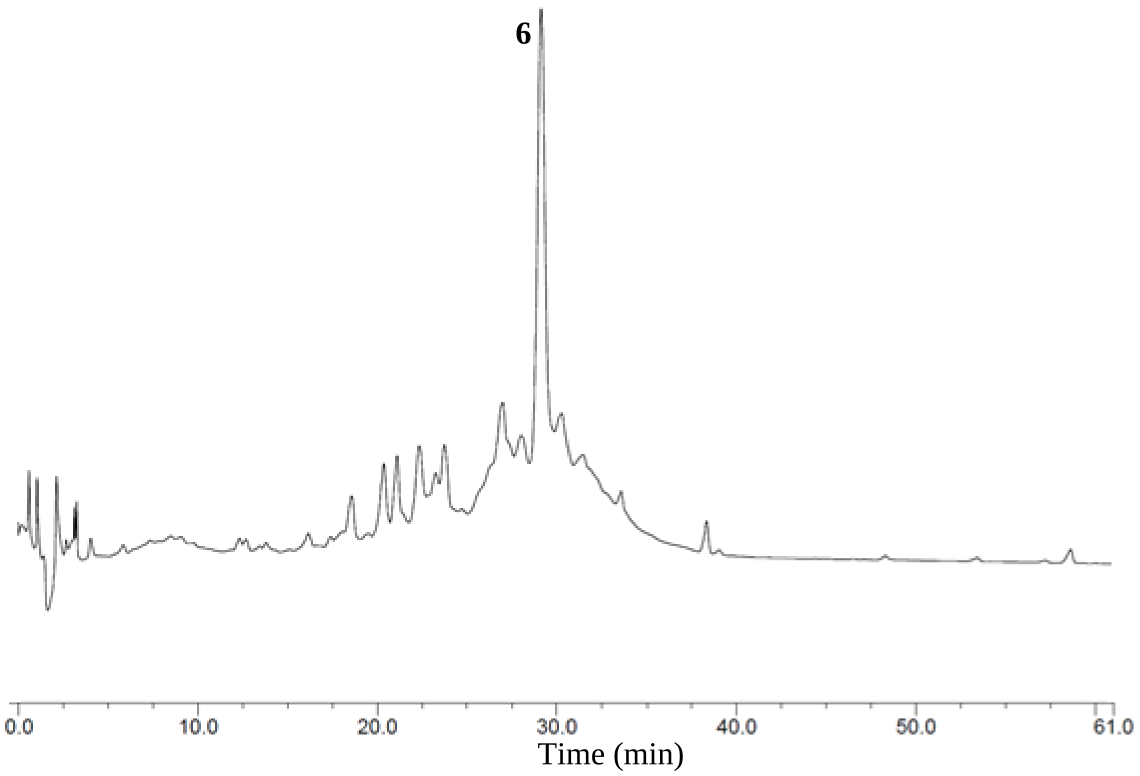

Figure S41. Analytical RP-HPLC chromatogram (214 nm) of crude peptide, **6**, t_R = 29.2 min. Chromatographic separations were performed on a Thermo Scientific Dionex Ultimate 3000 HPLC using a XTerra® MS C-18 column (5 µm; 4.6 × 150 mm) and a linear gradient of 5 – 65% B in 60 min at room temperature, *ca.* 1% B per min at a flow rate of 1.0 mL/min. Buffer A: H_2O containing 0.1% TFA (*v/v*); Buffer B: acetonitrile containing 0.1 % TFA (*v/v*).

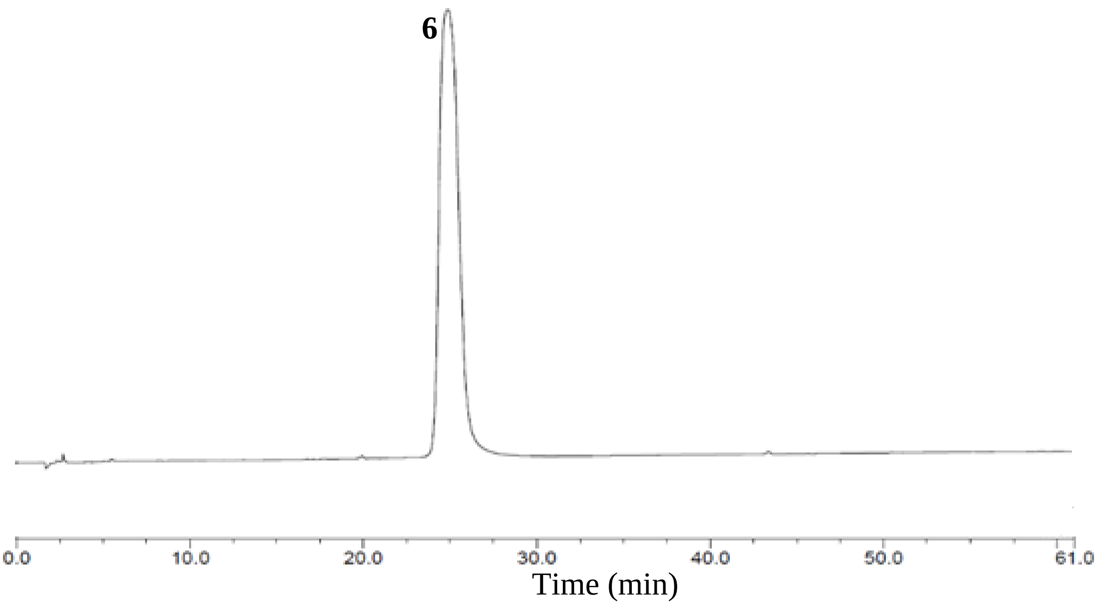

Figure S42. Analytical RP-HPLC chromatogram (214 nm) of purified peptide **6**, t_R = 24.88 min. Chromatographic separations were performed on a Thermo Scientific Dionex Ultimate 3000 HPLC using a XTerra® MS C-18 column (5 µm; 4.6 x 150 mm) and a linear gradient of 5 – 65% B in 60 min at room temperature, *ca.* 1% B per min at a flow rate of 1.0 mL/min. Buffer A: H_2O containing 0.1% TFA (*v/v*); Buffer B: acetonitrile containing 0.1 % TFA (*v/v*).

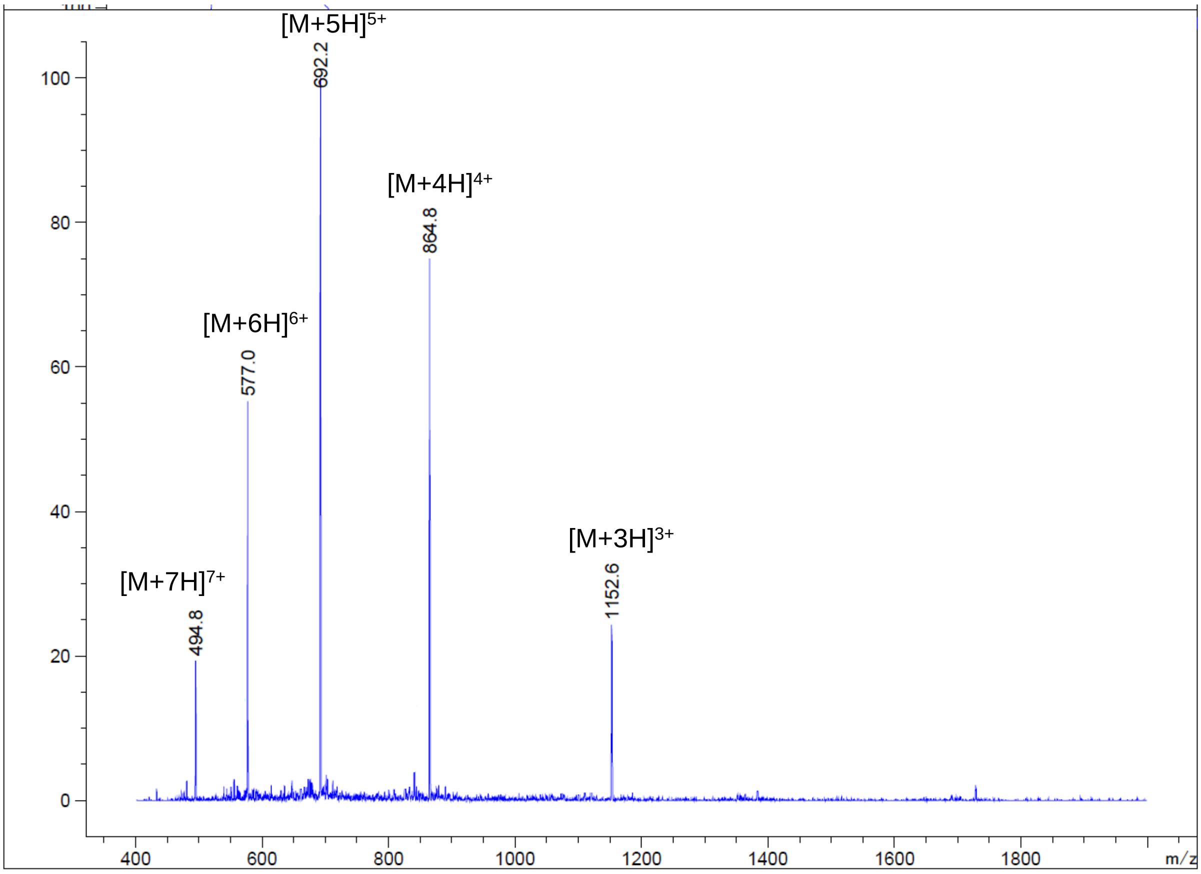

Figure S43. ESI-MS *m/z* for purified peptide, **6;** [$C_{156}H_{270}N_{48}O_{40}$] $[M+2H]^{2+}$ calculated:1729.03, observed 1729.3; $[M+3H]^{3+}$ calculated: 1153.02, observed 1152.6; $[M+4H]^{4+}$ calculated: 865.02, observed 864.8; $[M+5H]^{5+}$ calculated: 692.21, observed 692.2; $[M+6H]^{6+}$ calculated: 577.01, observed 577.0; $[M+7H]^{7+}$ calculated: 494.72, observed 494.8; Mass calculated at 3456.06 Da.

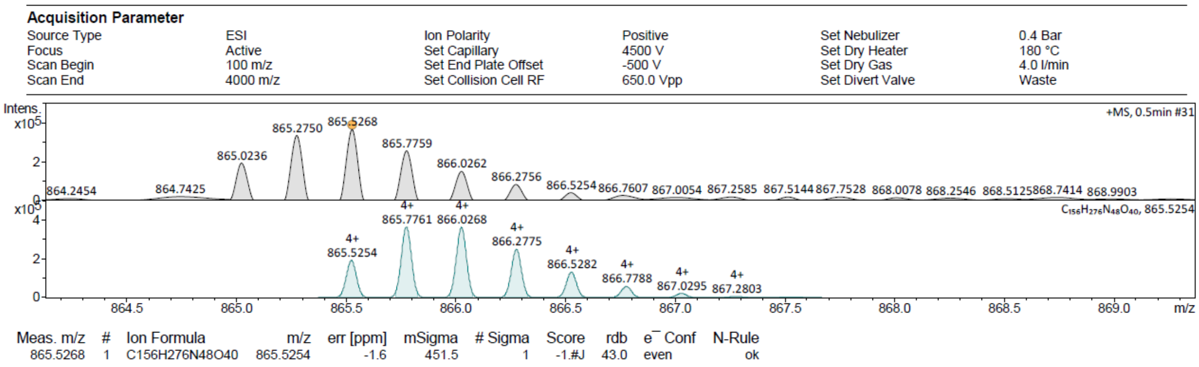

Figure S44. ESI-HRMS, formula analysis for $[C_{156}H_{270}N_{48}O_{40}+4H]^{4+}$ calculated: 865.5254 observed 865.5268.

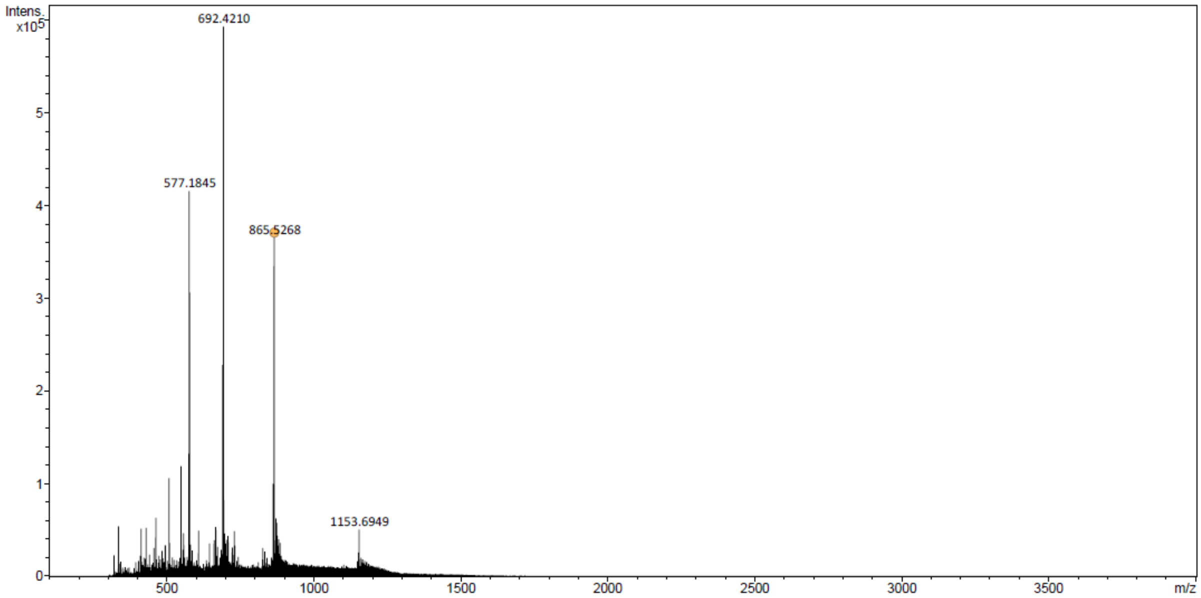

Figure S45. ESI-HRMS *m/z* calculated for, 6 $[C_{156}H_{270}N_{48}O_{40}+4H]^{4+}$ 865.5254 observed 865.5268.

S.13 Synthesis of Lys^3 / Asp^7 lactam ring mimetic (7)

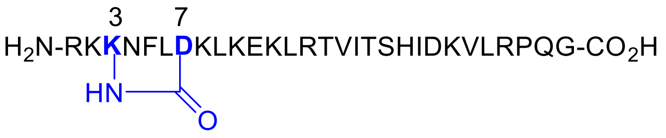

7

Method 1: Loading TentaGel® S NH_2 with HMPA linker

TentaGel® S NH_2 resin (600 mg, 0.15 mmol, 0.25 mmolg^{-1}) was pre-swollen in CH_2Cl_2 (5 mL, 30 min). CH_2Cl_2 drained, 4-(Hydroxymethyl)phenoxyacetic acid (HMPA linker) (136.64 mg, 0.5 mmol, 5 equiv.) and *6*-Cl-HOBt (127.18 mg, 0.5 mmol, 5 equiv.) dissolved in DMF (2.0 mL) followed by addition of DIC (117.43 µl, 0.5 mmol, 5 equiv.) added to the swollen resin. The reaction mixture was gently agitated at room temperature for 3 hours. The resin was filtered and washed with DMF (3 × 3 mL) after which a negative ninhydrin test confirmed successful coupling.

Method 2: Resin functionalisation for *C*-terminal acid peptides

The symmetrical anhydride of the N^{α}-Fmoc protected *C*-terminal amino acid was prepared with Fmoc-Gly-OH (445.97 mg, 1.0 mmol, 10 equiv.) by reacting with DIC (117.43 µl, 0.5mmol, 5 equiv.) in CH_2Cl_2 for 10 mins at room temperature. The resulting symmetric anhydride (0.5 mmol, 5 equiv.) was manually esterified with the resin bound HMPA linker via acyl transfer, using a catalytic amount of DMAP (1.32 mg, 0.01 mmol, 0.1 equiv.) in DMF for 2 h at room temperature.

Method 3: General procedure for removal of N^{α}-Fmoc protecting group

The peptidyl resin was treated with a solution of 30% piperidine, 5% formic acid in DMF (40 s, 10 mL, *v/v/v*) at 65 ^{o}C and resin washed by DMF (40s, 10 mL) removing any residual capping solution.

Method 4: General procedure for amino acid coupling using HATU

The freshly prepared amino acid (0.30 M, 20 equiv.) with coupling reagent HATU (0.28 M, 19.5 equiv.) was dissolved in DMF (10 mL). DIPEA (1358.69 µL, 6.0 mmol, 39 equiv.) was added to pre-activate the coupling solution 20 seconds prior to delivery to the on-resin peptide bearing free N^{α}-amino group. The activated amino acid solution is fully delivered (~40 s, 10

mL) and the delivery line is washed by drawing a further DMF (20 s, 4 mL). The peptidyl resin is further washed with DMF, removing any residual amino coupling solution (40 s, 10 mL).

Method 5: Coupling procedures for Fmoc-His(Trt)-OH

Freshly prepared Fmoc-His(Trt)-OH (1.859 g, 20 equiv.) and coupling reagent PyAOP (1.525 g, 2.9 mmol, 19.5 equiv.) were dissolved in DMF (10 mL). *Sym*-collidine (779 µl, 39 equiv.) was added to pre-activate the coupling solution 20 seconds prior to delivery to the on-resin peptide bearing free N^{α}-amino group. The activated amino acid solution is fully delivered (~40 s, 10 mL) and the delivery line is washed by drawing a further DMF (20 s, 4 mL). The peptidyl resin is further washed with DMF, removing any residual amino coupling solution (40 s, 10 mL).

Method 6: General procedure for capping the free amino groups:

Fmoc-protected peptidyl resin was treated with a solution of 20% acetic anhydride in DMF (40 s, 10 mL, *v/v*) at 65 ^{o}C and resin washed thorough by DMF (80s, 20 mL) removing any residual capping solution.

Method 7: General procedure for removal of 4-Methyltrityl (Mtt) and 2-phenylisopropyl (*O*-2-PhiPr) orthogonal protecting groups

Peptidyl resin was treated with 1.0% TFA in CH_2Cl_2 (10 × 3 min, *v/v*) and agitated at room temperature. At the end of 10th treatment cycle the resin was thoroughly washed with CH_2Cl_2 (5 × 5 mL) and with DMF (3 × 5 mL).

Method 8: General procedure to form lactam bridging between residue Lys^{3} and Asp^{7}

Peptidyl resin was treated with PyAOP (391 mg, 5 equiv.) and DIPEA (130.64 µl, 5 equiv.) in DMF (5 mL) and agitated for 4 h at room temperature.

Method 9: TFA mediated resin cleavage and global deprotection

Peptidyl resin was treated with a mixture of TFA/H_2O/TIS (95:2.5:2.5, 10 mL, *v/v/v*) for 120 min. The filtrate was concentrated under a gentle stream of N_2 followed by the addition of cold diethyl ether to form a precipitate. The mixture was centrifuged, and the solution was carefully decanted off and discarded. The solid pellet was dissolved H_2O (20 mL) and lyophilised.

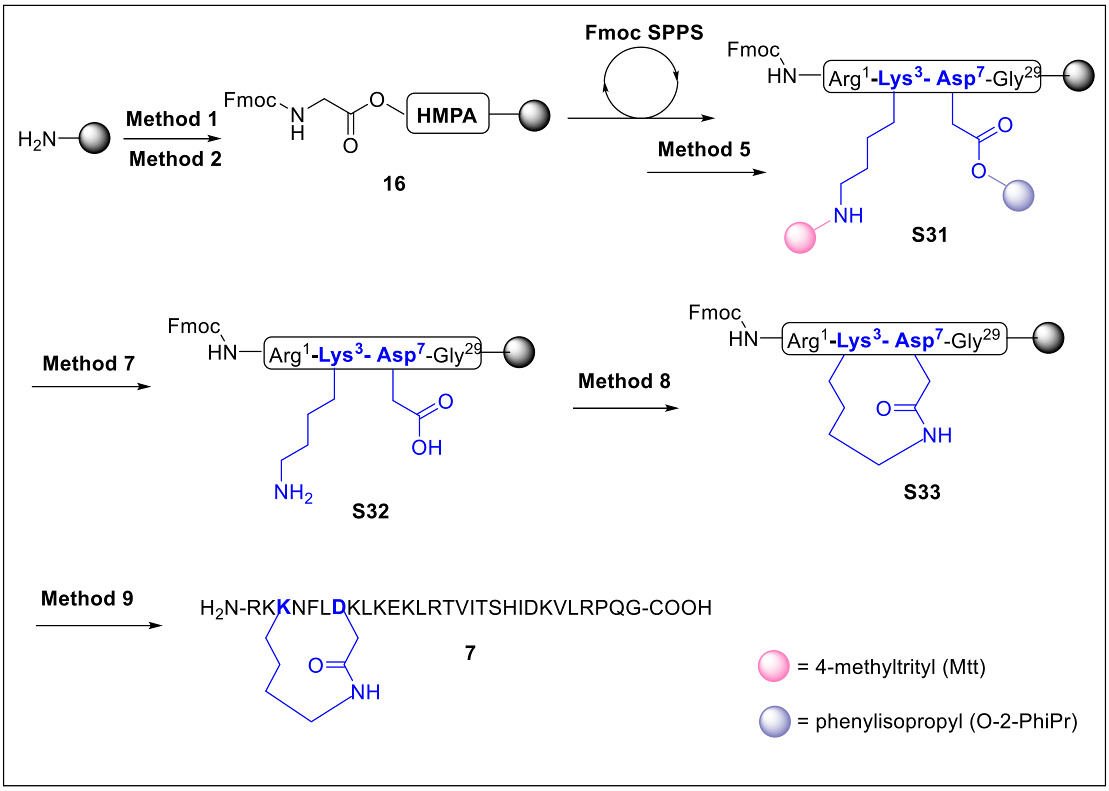

Scheme S12. Synthesis of analogues C7 using flow chemistry.

Fmoc-SPPS was used for the linear peptide synthesis employing TentaGel S NH_2 resin, functionalizing with HMPA linker using **Method 1**. Followed by symmetrical anhydride of the N^{α}-Fmoc protected *C*-terminal amino acid using **Method 2**. To NH_2-Gly-HMPA linker bound resin (0.15 mmol) was packed into the metal reactor for peptide synthesis under manual flow chemistry. The manual flow synthesis was initiated with pre-washing the resin with DMF (120 s, 30mL) and performing the initial removal of N^{α}-Fmoc protecting group using **Method 3**. The freshly made amino acids were appropriately coupled using **Method 4**, modified coupling conditions were used for the histidine residue, His^{10} according to **Method 5**. Unreacted amino groups were capped using **Method 6**, followed by Fmoc-removal using **Method 3**. Once the linear cathelicidin-PY was synthesised, with the N^{α}-Fmoc protecting group still present the peptidyl resin was treated with acid sensitive cocktail to remove orthogonal protecting group of Lys3 and Asp7 using **Method 7**. Side-chain-to-side-chain lactam cyclisation was carried out according to **Method 8**. The resulting peptide was cleaved using **Method 9** to afford **7** as a white solid (296.4 mg, yield 57%, crude purity 51%). The crude peptide was purified by semi-preparative RP-HPLC using a Dionex UltiMate® 3000 on a Phenomenex Gemini C_{18} column (10 x 150 mm, 5 mm) using a linear gradient of 5% to 65% over 60 min (*ca.* 1% B/min) with a flow rate of 4.5 mL/min. Fractions were collected and

analysed by ESI-MS for compound identification. Fractions that were identified with the correct *m/z* were collected, combined and lyophilised to afford **7** as a white amorphous powder (31.42 mg, yield 10.6%, 99.1% purity).

ESI-MS: *m/z* calculated for $[C_{155}H_{268}N_{48}O_{40}+4H]^{4+}$ calculated:861.7695, observed 861.7715. **RP-HPLC:** $\mathbf{t_R}$ = 23.5 min.

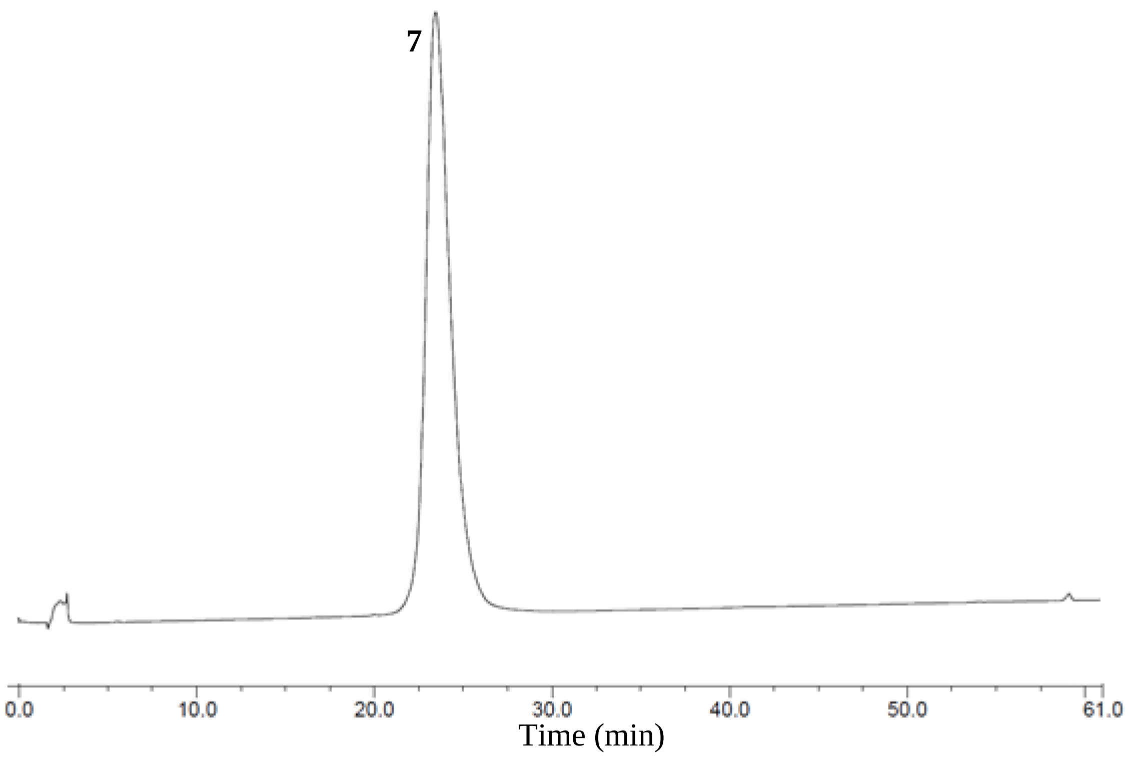

Figure S46. Analytical RP-HPLC chromatogram (214 nm) of purified peptide **7**, t_R = 23.5 min. Chromatographic separations were performed on a Thermo Scientific Dionex Ultimate 3000 HPLC using a XTerra® MS C-18 column (5 μm; 4.6 x 150 mm) and a linear gradient of 5 – 65% B in 60 min at room temperature, *ca.* 1% B per min at a flow rate of 1.0 mL/min. Buffer A: H_2O containing 0.1% TFA (*v/v*); Buffer B: acetonitrile containing 0.1 % TFA (*v/v*).

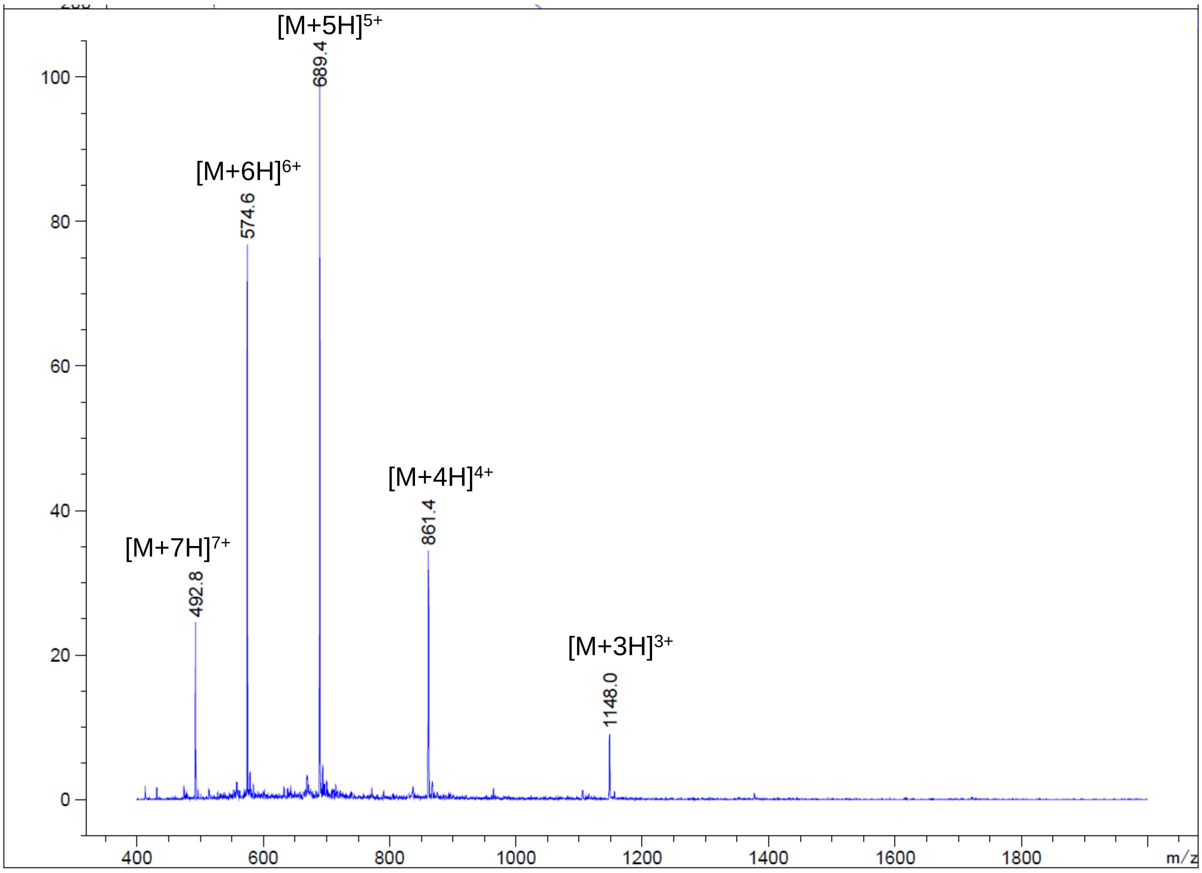

Figure S47. ESI-MS *m/z* for purified peptide **7**; [$C_{155}H_{268}N_{48}O_{40}$] $[M+3H]^{3+}$ calculated: 1148.34, observed 1148.0; $[M+4H]^{4+}$ calculated: 861.51, observed 861.4; $[M+5H]^{5+}$ calculated: 689.41, observed 689.4; $[M+6H]^{6+}$ calculated: 574.67, observed 574.6; $[M+7H]^{7+}$ calculated: 492.72, observed 492.8; Mass calculated at 3442.04 Da.

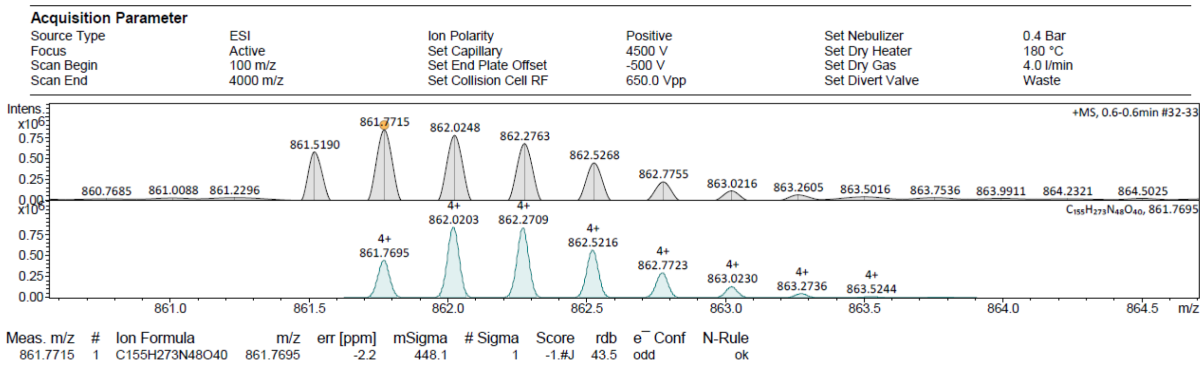

Figure S48. ESI-HRMS, formula analysis for $[C_{155}H_{268}N_{48}O_{40}+4H]^{4+}$ calculated:861.7695, observed 861.7715.

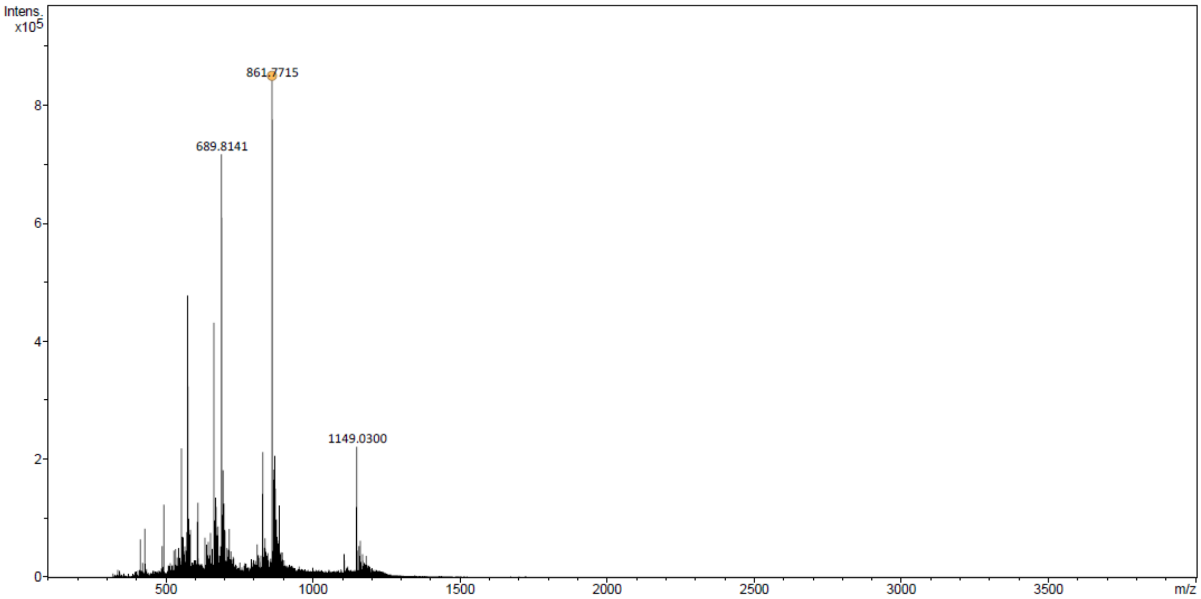

Figure S49. ESI-HRMS, m/z calculated for, **7**, $[C_{155}H_{268}N_{48}O_{40}+4H]^{4+}$ 861.7695, observed 861.7715.

S.13 Synthesis of Asp^{3} / X^{7} lactam ring mimetic (8)

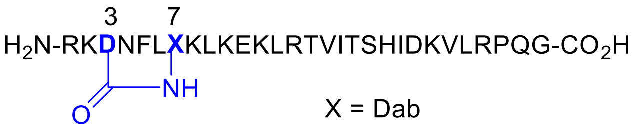

8

Method 1: Loading TentaGel® S NH_2 with HMPA linker

TentaGel® S NH_2 resin (600 mg, 0.15 mmol, 0.25 mmolg^{-1}) was pre-swollen in CH_2Cl_2 (5 mL, 30 min). CH_2Cl_2 drained, 4-(Hydroxymethyl)phenoxyacetic acid (HMPA linker) (136.64 mg, 0.5 mmol, 5 equiv.) and *6*-Cl-HOBt (127.18 mg, 0.5 mmol, 5 equiv.) dissolved in DMF (2.0 mL) followed by addition of DIC (117.43 µl, 0.5 mmol, 5 equiv.) added to the swollen resin. The reaction mixture was gently agitated at room temperature for 3 hours. The resin was filtered and washed with DMF (3 × 3 mL) after which a negative ninhydrin test confirmed successful coupling.

Method 2: Resin functionalisation for *C*-terminal acid peptides

The symmetrical anhydride of the N$^{\alpha}$-Fmoc protected *C*-terminal amino acid was prepared with Fmoc-Gly-OH (445.97 mg, 1.0 mmol, 10 equiv.) by reacting with DIC (117.43 µl, 0.5mmol, 5 equiv.) in CH_2Cl_2 for 10 mins at room temperature. The resulting symmetric anhydride (0.5 mmol, 5 equiv.) was manually esterified with the resin bound HMPA linker via acyl transfer, using a catalytic amount of DMAP (1.32 mg, 0.01 mmol, 0.1 equiv.) in DMF for 2 h at room temperature.

Method 3: General procedure for removal of N$^{\alpha}$-Fmoc protecting group

The peptidyl resin was treated with a solution of 30% piperidine, 5% formic acid in DMF (40 s, 10 mL, *v/v/v*) at 65 ^{o}C and resin washed by DMF (40s, 10 mL) removing any residual capping solution.

Method 4: General procedure for amino acid coupling using HATU

The freshly prepared amino acid (0.30 M, 20 equiv.) with coupling reagent HATU (0.28 M, 19.5 equiv.) was dissolved in DMF (10 mL). DIPEA (1358.69 mL, 6.0 mmol, 39 equiv.) was added to pre-activate the coupling solution 20 seconds prior to delivery to the on-resin peptide bearing free N$^{\alpha}$-amino group. The activated amino acid solution is fully delivered (~40 s, 10

mL) and the delivery line is washed by drawing a further DMF (20 s, 4 mL). The peptidyl resin is further washed with DMF, removing any residual amino coupling solution (40 s, 10 mL).

Method 5: Coupling procedures for Fmoc-His(Trt)-OH

Freshly prepared Fmoc-His(Trt)-OH (1.859 g, 20 equiv.) in coupling reagent PyAOP (1.525 g, 2.9 mmol, 19.5 equiv.) was dissolved in DMF (10 mL). *Sym*-collidine (779 μl, 39 equiv.) was added to pre-activate the coupling solution 20 seconds prior to delivery to the on-resin peptide bearing free N^{α}-amino group. The activated amino acid solution is fully delivered (~40 s, 10 mL) and the delivery line is washed by drawing a further DMF (20 s, 4 mL). The peptidyl resin is further washed with DMF, removing any residual amino coupling solution (40 s, 10 mL).

Method 6: General procedure for capping the free amino groups:

Fmoc-protected peptidyl resin was treated with a solution of 20% acetic anhydride in DMF (40 s, 10 mL, *v/v*) at 65 ^{o}C and resin washed thorough by DMF (80s, 20 mL) removing any residual capping solution.

Method 7: tert-butyloxycarbonyl (Boc) anhydride protection of *N*-terminal amine

N^{α}-Fmoc was replaced by Boc protecting group using Boc anhydride (163.69 mg, 5 equiv.) in DIPEA (78.39 μl, 3 equiv.) with DMF (2 mL) was added and gently agitated at room temperature for 1 h.

Method 8: Removal of 1-(4,4-dimethyl-2,6-dioxocyclohex-1-ylidene)ethyl (Dde) orthogonal protecting group

Peptidyl resin was treated with 2% hydrazine in DMF (3 × 3 min, *v/v*) and agitated at room temperature. After 3rd cycle of treatment the resin was thoroughly washed with DMF (3 × 5 mL).

Method 9: General procedure for removal of phenylisopropyl (*O*-2-PhiPr) orthogonal protecting groups

Peptidyl resin was treated with 1.0% TFA in CH_2Cl_2 (10 × 3 min, *v/v*) and agitated at room temperature. At the end of 10th treatment cycle the resin was thoroughly washed with CH_2Cl_2 (5 × 5 mL) and with DMF (3 × 5 mL).

Method 10: General procedure to form lactam bridging between residue Asp^3 and Dab^7

Peptidyl resin was treated with PyAOP (391 mg, 5 equiv.) and *sym*-collidine (98.68 µl, 5 equiv.) in DMF (5 mL) and agitated at room temperature for 24 h.

Method 11: TFA mediated resin cleavage and global deprotection

Peptidyl resin was treated with a mixture of TFA/H_2O/TIS (95:2.5:2.5, 10 mL, *v/v/v*) for 120 min. The filtrate was concentrated under a gentle stream of N_2 followed by the addition of cold diethyl ether to form a precipitate. The mixture was centrifuged, and the solution was carefully decanted off and discarded. The solid pellet was dissolved H_2O (20 mL) and lyophilised.

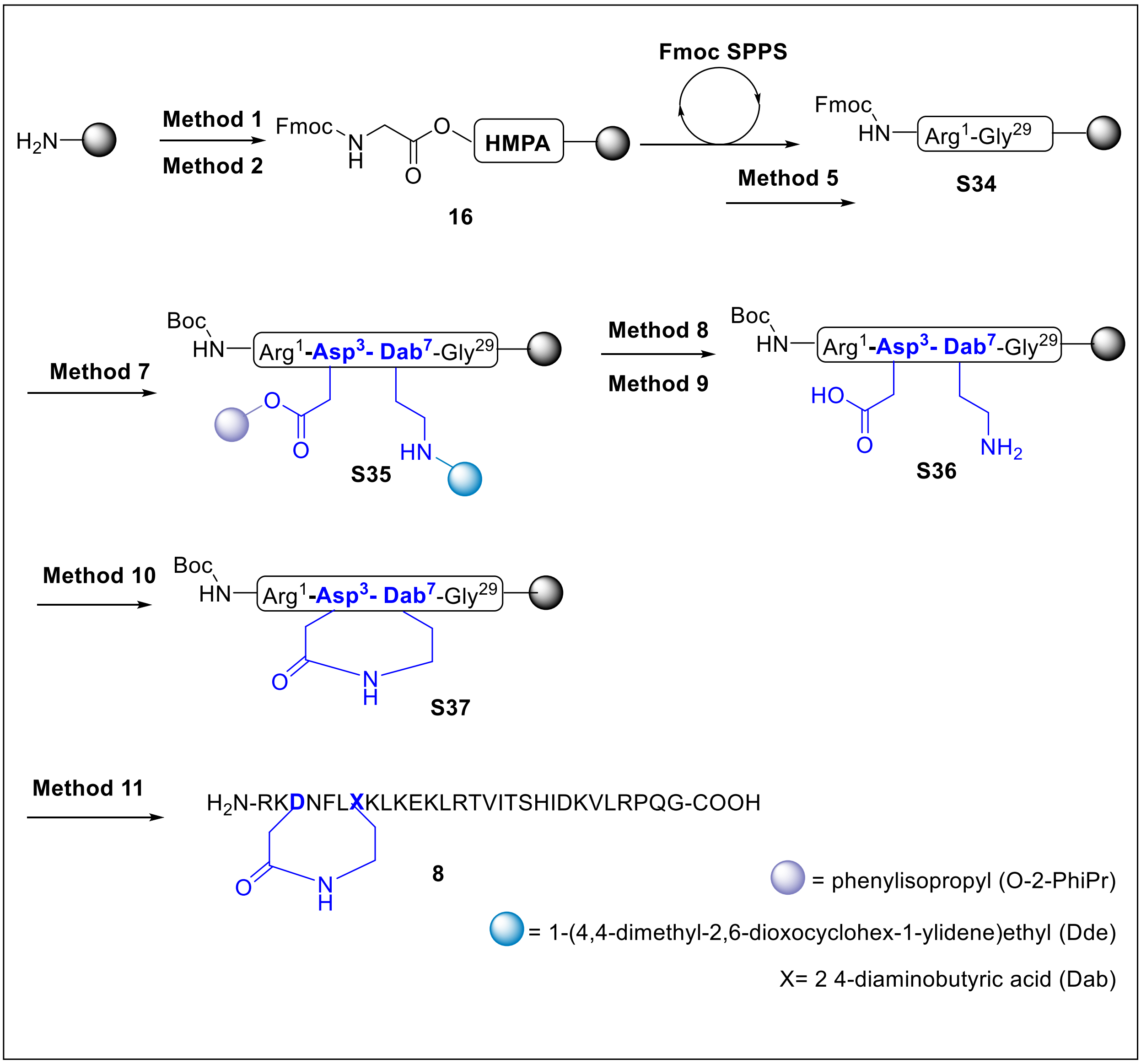

Scheme S13. Synthesis of analogues C8 using flow chemistry.

Fmoc-SPPS was used for the linear peptide synthesis employing TentaGel S NH_2 resin, functionalizing with HMPA linker using **Method 1**. Followed by symmetrical anhydride of the *N*$^{\alpha}$-Fmoc protected *C*-terminal amino acid using **Method 2** To NH_2-Gly-HMPA linker bound resin (0.15 mmol) was packed into the metal reactor for peptide synthesis under manual flow chemistry. The manual flow synthesis was initiated with pre-washing the resin with DMF (120 s, 30mL) and performing the initial removal of N$^{\alpha}$-Fmoc protecting group using **Method 3**. The freshly made amino acids were appropriately coupled using **Method 4**, modified coupling conditions were used for the histidine residue, His^{10} according to **Method 5**. Unreacted amino groups were capped using **Method 6**, followed by Fmoc-removal using **Method 3**. Once the linear cathelicidin-PY was synthesised, with the N$^{\alpha}$-Fmoc protecting group was exchanged for a N$^{\alpha}$-Boc protecting group using the **Method 7**. Orthogoal selective removal of Dde was carried out using **Method 8** and acid sensitive removal using **Method 9**. Following orthogonal protecting group removal, side-chain-to-side-chain lactam cyclisation was carried out according to **Method 10**. The resulting peptide was cleaved using **Method 11** to afford **8** as a white solid (186.92 mg, 36.5% yield, crude purity 48%). The crude peptide was purified by semi-preparative RP-HPLC using a Dionex UltiMate® 3000 on a Phenomenex Gemini C_{18} column (10 x 150 mm, 5 mm) using a linear gradient of 5% to 65% over 60 min (*ca*. 1% B/min) with a flow rate of 4.5 mL/min. Fractions were collected and analysed by ESI-MS for compound identification. Fractions that were identified with the correct *m/z* were collected, combined and lyophilised to afford **8** as a white amorphous powder (14.17 mg, yield 7.58 %, 99% purity).

ESI-MS: *m/z* calculated for $[C_{153}H_{264}N_{48}O_{40}+4H]^{4+}$ calculated:854.7617 observed 854.7637. **RP-HPLC: t_R** = 26.3 min.

8

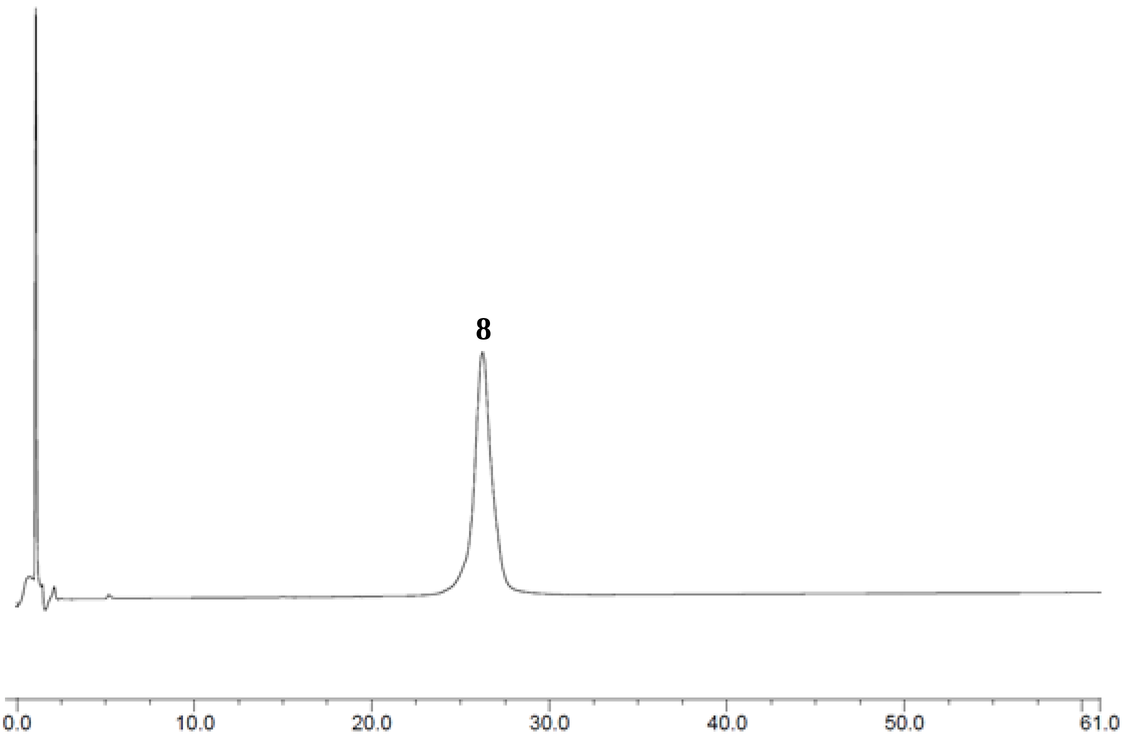

Time (min)

Figure S50. Analytical RP-HPLC chromatogram (214 nm) of purified peptide, **8**; t_R = 26.3 min. Chromatographic separations were performed on a Thermo Scientific Dionex Ultimate 3000 HPLC using a XTerra® MS C-18 column (5 μm; 4.6 x 150 mm) and a linear gradient of 5 – 65% B in 60 min at room temperature, *ca.* 1% B per min at a flow rate of 1.0 mL/min. Buffer A: H_2O containing 0.1% TFA (*v/v*); Buffer B: acetonitrile containing 0.1 % TFA (*v/v*).

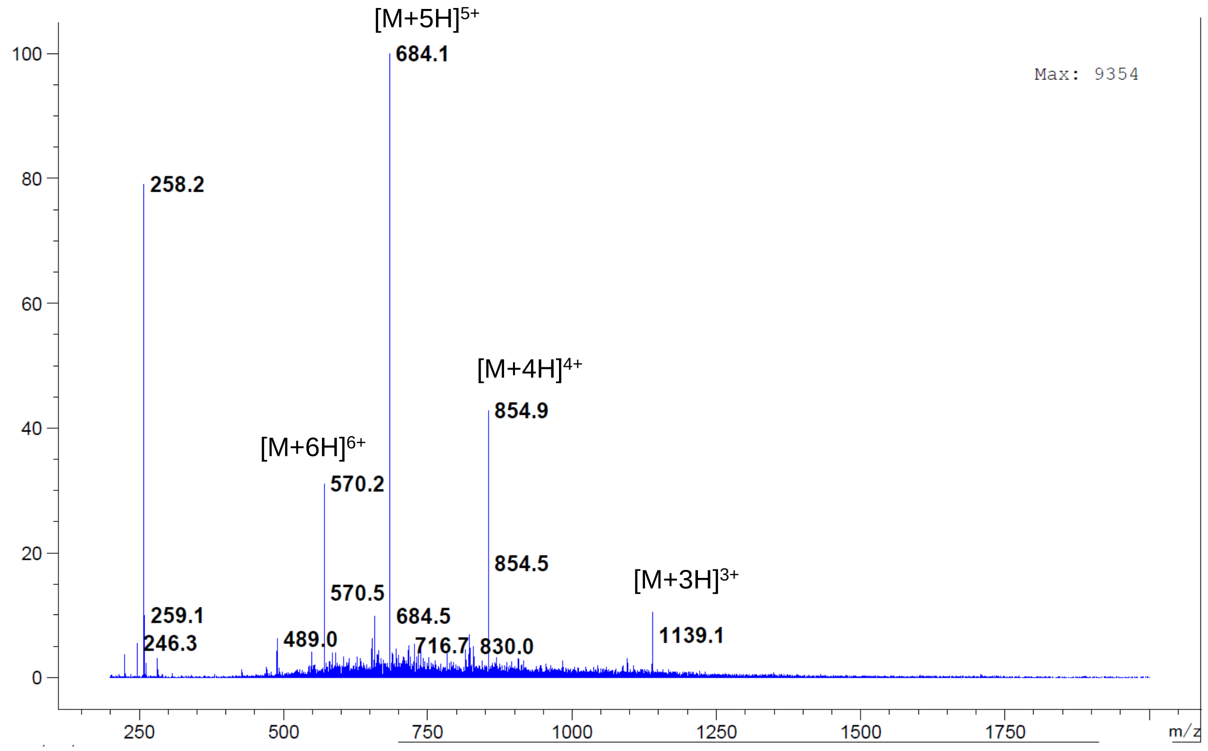

Figure S51. ESI-MS *m/z* for purified peptide **8**; [$C_{153}H_{264}N_{48}O_{40}$] $[M+3H]^{3+}$ calculated: 1139.00, observed 1139.1; $[M+4H]^{4+}$ calculated: 854.50, observed 854.9; $[M+5H]^{5+}$ calculated: 683.80, observed 684.1; $[M+6H]^{6+}$ calculated: 570.00, observed 570.2; Mass calculated at 3414.01 Da.

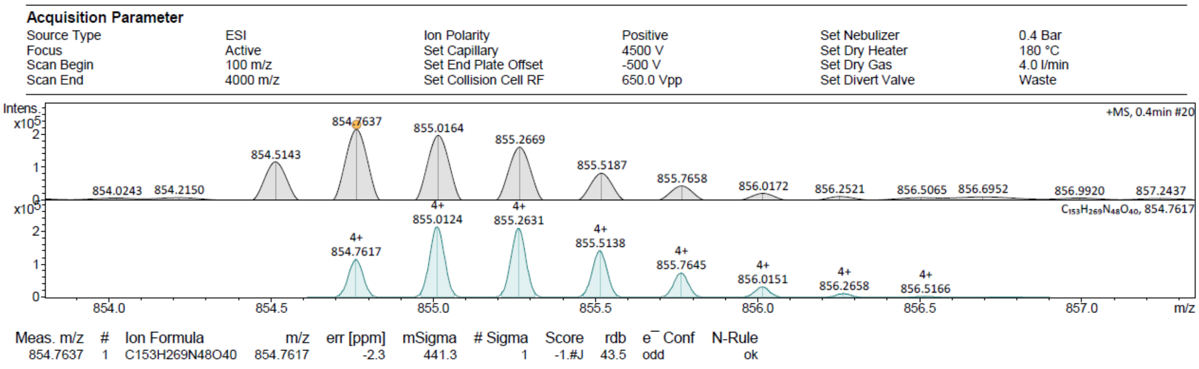

Figure S52. ESI-HRMS, formula analysis for $[C_{153}H_{264}N_{48}O_{40}+4H]^{4+}$ calculated:854.7617 observed 854.7637.

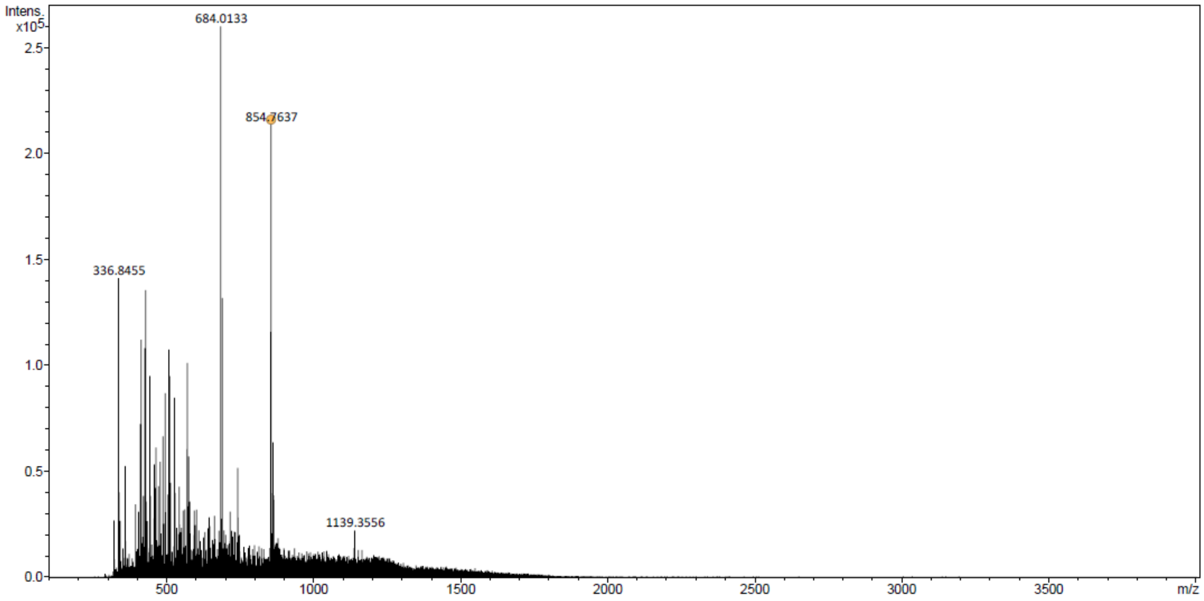

Figure S53. ESI-HRMS, m/z calculated for, **8**, $[C_{153}H_{264}N_{48}O_{40}+4H]^{4+}$ calculated:854.7617 observed 854.7637.

S.14 Synthesis of Glu^3 / X^7 lactam ring mimetic (9)

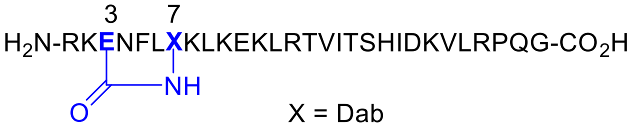

9

Fmoc-SPPS was used for the linear peptide synthesis employing TentaGel S NH_2 resin, functionalizing with HMPA linker using **Method 1**. Followed by symmetrical anhydride of the N^{α}-Fmoc protected *C*-terminal amino acid using **Method 2** To NH_2-Gly-HMPA linker bound resin (0.15 mmol) was packed into the metal reactor for peptide synthesis under manual flow chemistry. The manual flow synthesis was initiated with pre-washing the resin with DMF (120 s, 30mL) and performing the initial removal of N^{α}-Fmoc protecting group using **Method 3**. The freshly made amino acids were appropriately coupled using **Method 4**, modified coupling conditions were used for the histidine residue, His^{10} according to **Method 5**. Unreacted amino groups were capped using **Method 6**, followed by Fmoc-removal using **Method 3**. Once the linear cathelicidin-PY was synthesised, with the N^{α}-Fmoc protecting group was exchanged for a N^{α}-Boc protecting group using the **Method 7**. Orthogoal selective removal of Dde was carried out using **Method 8** and acid sensitive removal using **Method 9**. Following orthogonal protecting group removal, side-chain-to-side-chain lactam cyclisation was carried out according to **Method 10**. The resulting peptide was cleaved using **Method 11** to afford **9** as a white solid (191.2 mg, yield 37%, crude purity 51%). The crude peptide was purified by semi-preparative RP-HPLC using a Dionex UltiMate® 3000 on a Phenomenex Gemini C_{18} column (10 x 150 mm, 5 mm) using a linear gradient of 5% to 65% over 60 min (*ca*. 1% B/min) with a flow rate of 4.5 mL/min. Fractions were collected and analysed by ESI-MS for compound identification. Fractions that were identified with the correct *m/z* were collected, combined and lyophilised to afford **9** as a white amorphous powder (13.38 mg, yield 7%, 95.1% purity).

ESI-MS: *m/z* calculated for $[C_{154}H_{266}N_{48}O_{40}]^{3+}$ 1143.7; observed 1144.3. **RP-HPLC: t_R** = 22.5 min.

Method 1: Loading TentaGel® S NH_2 with HMPA linker

TentaGel® S NH_2 resin (600 mg, 0.15 mmol, 0.25 mmolg^{-1}) was pre-swollen in CH_2Cl_2 (5 mL, 30 min). CH_2Cl_2 drained, 4-(Hydroxymethyl)phenoxyacetic acid (HMPA linker) (136.64 mg,

0.5 mmol, 5 equiv.) and *6*-Cl-HOBt (127.18 mg, 0.5 mmol, 5 equiv.) dissolved in DMF (2.0 mL) followed by addition of DIC (117.43 μl, 0.5 mmol, 5 equiv.) added to the swollen resin. The reaction mixture was gently agitated at room temperature for 3 hours. The resin was filtered and washed with DMF (3 × 3 mL) after which a negative ninhydrin test confirmed successful coupling.

Method 2: Resin functionalisation for *C*-terminal acid peptides

The symmetrical anhydride of the N^{α}-Fmoc protected *C*-terminal amino acid was prepared with Fmoc-Gly-OH (445.97 mg, 1.0 mmol, 10 equiv.) by reacting with DIC (117.43 μl, 0.5mmol, 5 equiv.) in CH_2Cl_2 for 10 mins at room temperature. The resulting symmetric anhydride (0.5 mmol, 5 equiv.) was manually esterified with the resin bound HMPA linker via acyl transfer, using a catalytic amount of DMAP (1.32 mg, 0.01 mmol, 0.1 equiv.) in DMF for 2 h at room temperature.

Method 3: General procedure for removal of N^{α}-Fmoc protecting group

The peptidyl resin was treated with a solution of 30% piperidine, 5% formic acid in DMF (40 s, 10 mL, *v/v/v*) at 65 ^{o}C and resin washed by DMF (40s, 10 mL) removing any residual capping solution.

Method 4: General procedure for amino acid coupling using HATU

The freshly prepared amino acid (0.30 M, 20 equiv.) with coupling reagent HATU (0.28 M, 19.5 equiv.) was dissolved in DMF (10 mL). DIPEA (1358.69 mL, 6.0 mmol, 39 equiv.) was added to pre-activate the coupling solution 20 seconds prior to delivery to the on-resin peptide bearing free N^{α}-amino group. The activated amino acid solution is fully delivered (~40 s, 10 mL) and the delivery line is washed by drawing a further DMF (20 s, 4 mL). The peptidyl resin is further washed with DMF, removing any residual amino coupling solution (40 s, 10 mL).

Method 5: Coupling procedures for Fmoc-His(Trt)-OH

Freshly prepared Fmoc-His(Trt)-OH (1.859 g, 20 equiv.) in coupling reagent PyAOP (1.525 g, 2.9 mmol, 19.5 equiv.) was dissolved in DMF (10 mL). *Sym*-collidine (779 μl, 39 equiv.) was added to pre-activate the coupling solution 20 seconds prior to delivery to the on-resin peptide bearing free N^{α}-amino group. The activated amino acid solution is fully delivered (~40 s, 10

mL) and the delivery line is washed by drawing a further DMF (20 s, 4 mL). The peptidyl resin is further washed with DMF, removing any residual amino coupling solution (40 s, 10 mL).

Method 6: General procedure for capping the free amino groups:

Fmoc-protected peptidyl resin was treated with a solution of 20% acetic anhydride in DMF (40 s, 10 mL, *v/v*) at 65 ^{o}C and resin washed thorough by DMF (80s, 20 mL) removing any residual capping solution.

Method 7: tert-butyloxycarbonyl (Boc) anhydride protection of *N*-terminal amine

N^{α}-Fmoc was replaced by Boc protecting group using Boc anhydride (163.69 mg, 5 equiv.) in DIPEA (78.39 µl, 3 equiv.) with DMF (2 mL) was added and gently agitated at room temperature for 1 h.

Method 8: Removal of 1-(4,4-dimethyl-2,6-dioxocyclohex-1-ylidene)ethyl (Dde) orthogonal protecting group

Peptidyl resin was treated with 2% hydrazine in DMF (3 × 3 min, *v/v*) and agitated at room temperature. After 3rd cycle of treatment the resin was thoroughly washed with DMF (3 × 5 mL).

Method 9: General procedure for removal of phenylisopropyl (*O*-2-PhiPr) orthogonal protecting groups

Peptidyl resin was treated with 1.0% TFA in CH_2Cl_2 (10 × 3 min, *v/v*) and agitated at room temperature. At the end of 10th treatment cycle the resin was thoroughly washed with CH_2Cl_2 (5 × 5 mL) and with DMF (3 × 5 mL).

Method 10: General procedure to form lactam bridging between residue Glu^{3} and Dab^{7}

Peptidyl resin was treated with PyAOP (391 mg, 5 equiv.) and *sym*-collidine (98.68 µl, 5 equiv.) in DMF (5 mL) and agitated at room temperature for 24 h.

Method 11: TFA mediated resin cleavage and global deprotection

Peptidyl resin was treated with a mixture of TFA/H_2O/TIS (95:2.5:2.5, 10 mL, *v/v/v*) for 120 min. The filtrate was concentrated under a gentle stream of N_2 followed by the addition of cold

diethyl ether to form a precipitate. The mixture was centrifuged, and the solution was carefully decanted off and discarded. The solid pellet was dissolved H_2O (20 mL) and lyophilised.

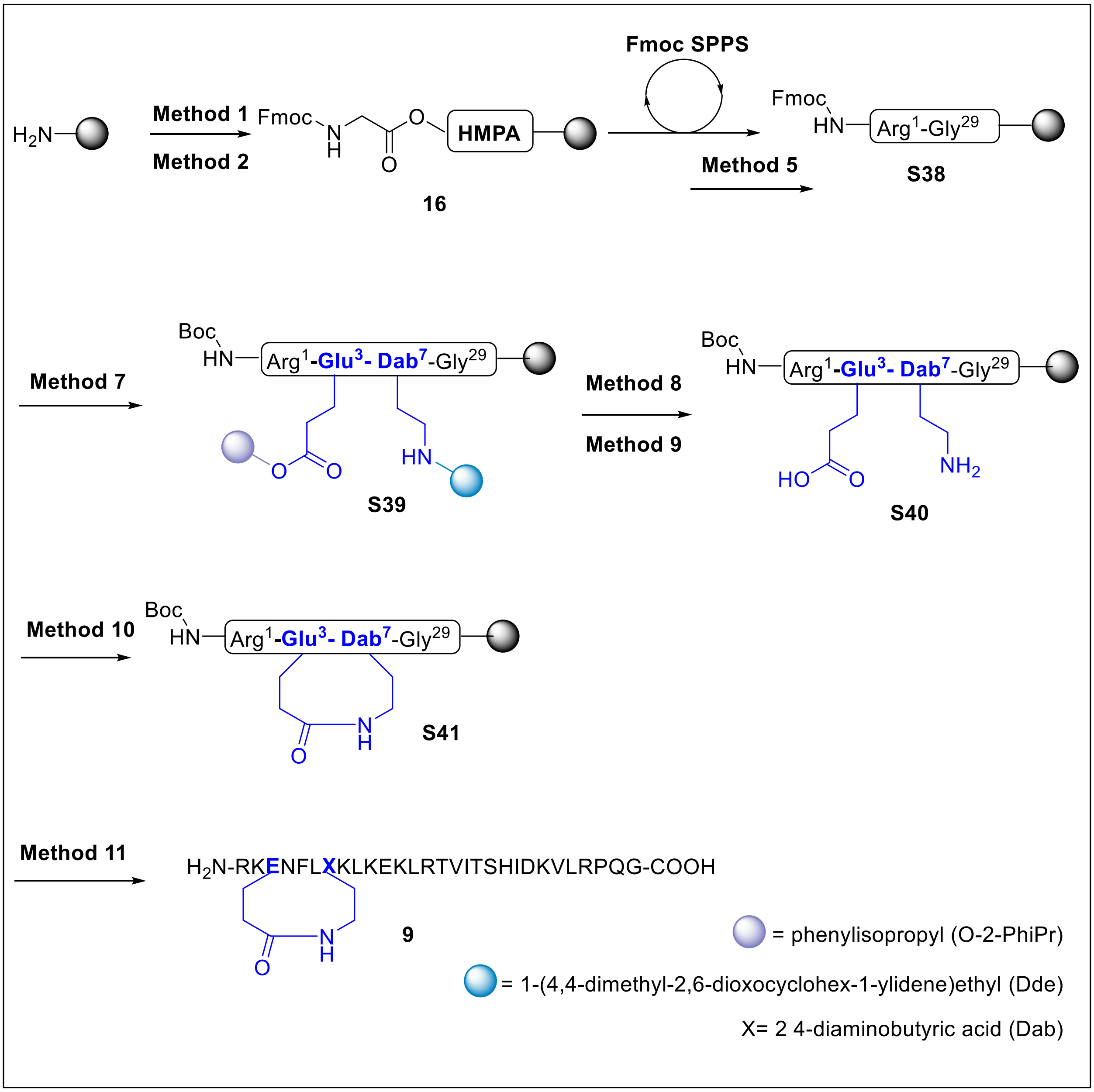

Scheme S14. Synthesis of analogues C9 using flow chemistry.

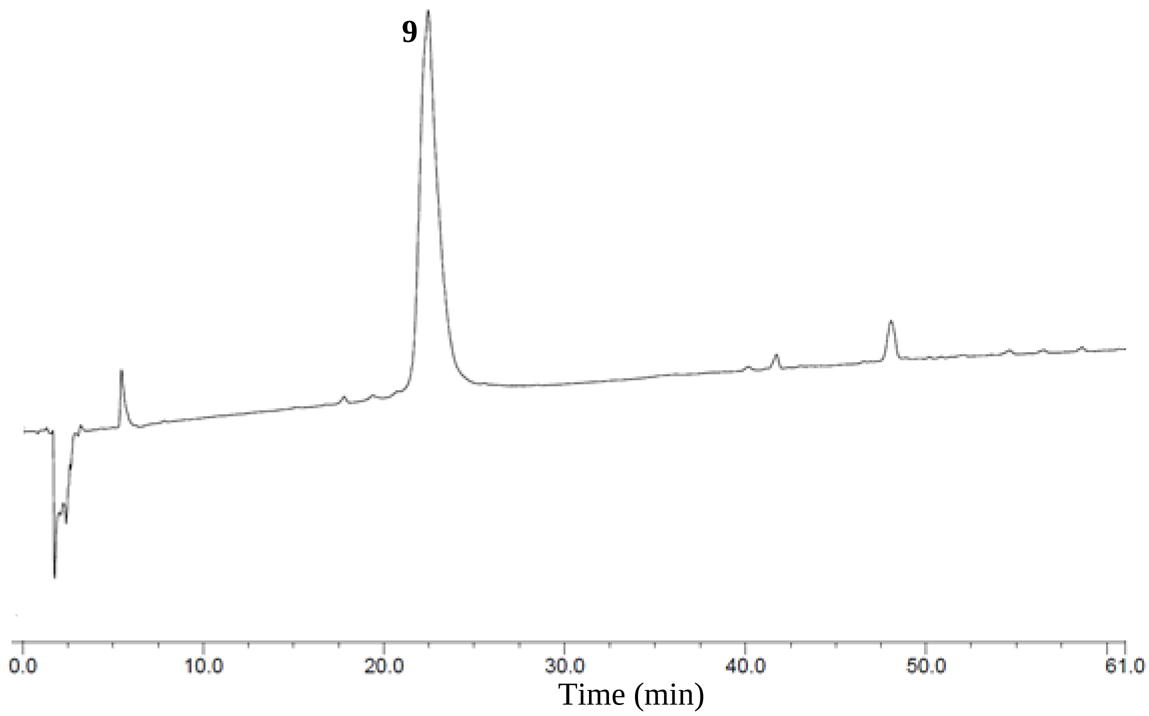

Figure S54. Analytical RP-HPLC chromatogram (214 nm) of purified peptide, **9**; t_R = 22.5 min. Chromatographic separations were performed on a Thermo Scientific Dionex Ultimate 3000 HPLC using a XTerra® MS C-18 column (5 μm; 4.6 x 150 mm) and a linear gradient of 5 – 65% B in 60 min at room temperature, *ca.* 1% B per min at a flow rate of 1.0 mL/min. Buffer A: H_2O containing 0.1% TFA (*v/v*); Buffer B: acetonitrile containing 0.1 % TFA (*v/v*).

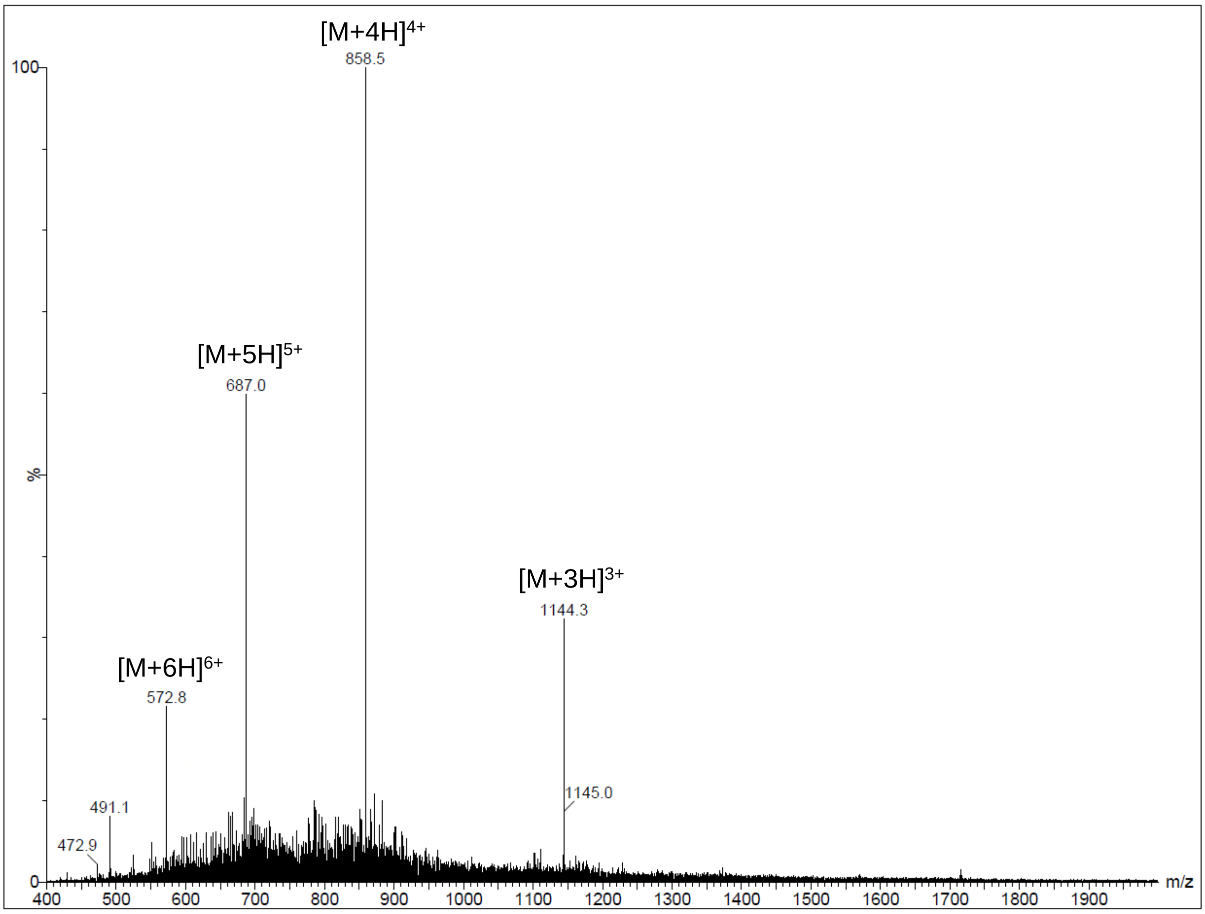

Figure S55. ESI-MS *m/z* for purified peptide, **9**; [$C_{154}H_{266}N_{48}O_{40}$] $[M+3H]^{3+}$ calculated: 1143.68, observed 1144.3; $[M+4H]^{4+}$ calculated: 858.01, observed 858.5; $[M+5H]^{5+}$ calculated: 686.61, observed 687; $[M+6H]^{6+}$ calculated: 572.34, observed 572.8; Mass calculated at 3428.03 Da.
